# Hormonal Contraception and HIV-1 Acquisition: Biological Mechanisms

**DOI:** 10.1210/er.2017-00103

**Published:** 2018-01-04

**Authors:** Janet P. Hapgood, Charu Kaushic, Zdenek Hel

**Affiliations:** 1 Department of Molecular and Cell Biology, University of Cape Town, Cape Town 7700, South Africa; 2 Institute of Infectious Disease and Molecular Medicine, University of Cape Town, Cape Town 7925, South Africa; 3 Department of Pathology and Molecular Medicine, McMaster University, Ontario L8S 4K1, Canada; 4 McMaster Immunology Research Centre, McMaster University, Hamilton, Ontario L8S 4K1, Canada; 5 Department of Pathology, University of Alabama at Birmingham, Birmingham, Alabama 35294; 6 Center for AIDS Research, University of Alabama at Birmingham, Birmingham, Alabama 35294

## Abstract

Access to effective affordable contraception is critical for individual and public health. A wide range of hormonal contraceptives (HCs), which differ in composition, concentration of the progestin component, frequency of dosage, and method of administration, is currently available globally. However, the options are rather limited in settings with restricted economic resources that frequently overlap with areas of high HIV-1 prevalence. The predominant contraceptive used in sub-Saharan Africa is the progestin-only three-monthly injectable depot medroxyprogesterone acetate. Determination of whether HCs affect HIV-1 acquisition has been hampered by behavioral differences potentially confounding clinical observational data. Meta-analysis of these studies shows a significant association between depot medroxyprogesterone acetate use and increased risk of HIV-1 acquisition, raising important concerns. No association was found for combined oral contraceptives containing levonorgestrel, nor for the two-monthly injectable contraceptive norethisterone enanthate, although data for norethisterone enanthate are limited. Susceptibility to HIV-1 and other sexually transmitted infections may, however, be dependent on the type of progestin present in the formulation. Several underlying biological mechanisms that may mediate the effect of HCs on HIV-1 and other sexually transmitted infection acquisition have been identified in clinical, animal, and *ex vivo* studies. A substantial gap exists in the translation of basic research into clinical practice and public health policy. To bridge this gap, we review the current knowledge of underlying mechanisms and biological effects of commonly used progestins. The review sheds light on issues critical for an informed choice of progestins for the identification of safe, effective, acceptable, and affordable contraceptive methods.

## Teaser

Review of clinical, animal, and *ex vivo* data on mechanisms whereby hormonal contraception may increase HIV-1 acquisition reveals evidence for several plausible biological mechanisms for medroxyprogesterone acetate.

**Essential Points**

- Medroxyprogesterone acetate (MPA) is an outlier amongst progestins, acting via the glucocorticoid receptor (GR) and exhibiting relatively potent glucocorticoid-like effects similar to cortisol, unlike norethisterone (NET) and levonorgestrel (LNG) and luteal phase progesterone
- MPA exerts potentially negative effects at concentrations found in serum of DMPA-IM users in animal and ex vivo models
- Current clinical, animal and ex vivo evidence supports a role for MPA in increasing the permeability of the female genital tract and promoting HIV-1 uptake
- There is strong evidence from clinical, mouse and ex vivo studies that MPA suppresses pDC and T cell function and suppresses select regulators of cellular and humoral systemic immunity
- Accumulated clinical and experimental data support the role of MPA in increasing the frequency of HIV-1 viral targets in the FGT and clinical and *ex vivo* evidence for increasing the levels of the CCR5 co-receptor for HIV-1 entry
- MPA exerts different effects compared to NET, LNG and luteal phase progesterone concentrations in some, but not all studies, suggesting that some of the potentially negative effects of MPA on HIV-1 acquisition are due to its glucocorticoid-like effects
- Together, the data provide a compelling case against the continuous use of DMPA-IM or DMPA-SQ in areas of high HIV-1 prevalence

Recent epidemiological evidence suggests that the intramuscularly injected progestin-only contraceptive depot medroxyprogesterone acetate (DMPA-IM) increases HIV-1 acquisition by 1.4-fold, unlike some other forms of hormonal contraception (HC). However, whether the results are affected by potential confounding factors remains unresolved. DMPA-IM is the major form of HC used in sub-Saharan Africa, which also has the highest worldwide HIV-1 prevalence, particularly in young women. Critical evaluations of combined clinical data together with animal and *ex vivo* data and the role of steroid receptors and progestin concentrations are required to understand the potential underlying biological mechanisms involved in the effects of HC on HIV-1 acquisition.

Access to safe contraception is a critical public health issue. Contraception provides direct benefits to women by providing control over their reproductive health and reducing the number of unintended pregnancies, as well as indirect benefits such as reducing the number of abortions, decreasing maternal and infant morbidity and mortality, and lowering the risk of vertical HIV-1 transmission. Inadequate access to effective contraceptive methods has severe consequences for both the individual and society. Depending on the region, up to 50% of unintended pregnancies in Africa end in abortion and the vast majority of abortions are unsafe (1, 2). Despite the indisputable overall benefit of contraception for public health, there is a growing concern that some forms of HC may increase HIV-1 and sexually transmitted infection (STI) acquisition in women. This is of particular concern in sub-Saharan Africa where women have limited choices for the types of contraceptive and are at high risk of HIV-1 acquisition. DMPA-IM is the most commonly used contraceptive in sub-Saharan Africa (3). The most recent systematic review of epidemiological studies suggests that new data increase the concern about a potential causal association between DMPA-IM usage and HIV-1 acquisition in women, whereas data investigating an association with several other forms of HC examined are limited or absent (4).

The biological mechanisms whereby HCs may modulate susceptibility to HIV-1 and other STIs in women are likely to be multifactorial and may include direct and/or indirect effects on several of the following mechanisms: (1) modulation of the structural integrity of the female genital tract (FGT); (2) modulation of the permeability and barrier defense properties of the epithelial layer in the FGT; (3) modulation of levels of soluble mediators and defense molecules secreted by the FGT (*e.g.*, cytokines, chemokines, antibodies, mucus, and antimicrobial and antiviral factors); (4) effects on the activity and frequency and HIV-1 coreceptor expression levels of circulation immune cells, FGT tissue-resident immune cells, and cells transmigrating into the FGT lumen [T cells, monocytes, neutrophils, and Langerhans cells (LCs)]; (5) effects on early innate immune responses in the FGT [plasmacytoid dendritic cells (pDCs), neutrophils, tissue macrophages, and natural killer cells]; (6) systemic effects on antigen presentation and induction of adaptive immune responses (T cells and B cells); (7) modulation of the replication of HIV-1 in the FGT; and (8) modulation of the composition and levels of vaginal microbiota and soluble factors produced by bacteria (5, 6). A review of available animal, *ex vivo*, and biochemical data on these mechanisms provides mechanistic insights into, and aids interpretation of, the epidemiological data. In this review, we discuss and summarize the evidence for the mechanisms mediated by commonly used progestins. In particular, we address the roles of progestin type and concentration on steroid receptor–mediated biological responses, on structure and function of the upper and lower reproductive tracts, on immune cells and their functions, on the soluble defense mechanisms in the FGT, on alterations in genital tract barrier function, and on the composition of the vaginal microbiota.

## Clinical and Public Health Perspectives on Contraception and HIV-1 Acquisition

Worldwide, the most prevalent forms of contraception are intrauterine devices (IUDs) and female sterilization, followed by combined oral contraceptives (COCs), male condoms, rhythm and withdrawal, injectables, and male sterilization (3). In contrast to other method mix patterns around the world, the most common form of contraception for those with low and middle incomes is injectables, followed by COCs. In 2015, >56 million women aged 15 to 49 who were married or in a union were estimated to use injectables worldwide (3). The number of women using injectables worldwide, including those not married or in a union, is likely to be substantially greater. In sub-Saharan Africa, ∼28% of women use some form of HC or non-HC, but a large unmet need (24%) remains (3). About 38% of all HC or non-HC users in sub-Saharan Africa (∼16.5 million women) use injectables (3). Injectable usage is particularly high in some countries such as South Africa where it accounted for nearly half (47%) of all contraceptive use in 2015 (3). The most common form of injectable contraception is the three-monthly intramuscular injection of 150 mg of DMPA (Depo-Provera or DMPA-IM), whereas norethisterone (NET) enanthate (NET-EN; also referred to as NET oenanthate or Nur-Isterate) at a two-monthly injection of 200 mg is used primarily in South Africa. Depo-Provera is being rolled out in India (7) and is increasingly being supplied in rural parts of sub-Saharan Africa through community-based distribution. In 2014, Pfizer announced the release of Sayana Press, a three-monthly, injectable, lower DMPA dose (104 mg), packaged in a single-use, subcutaneous, auto-disable syringe (Uniject) (8). Sayana Press, also known as DMPA-SQ or subcutaneous injectable contraceptive DMPA (DMPA-SC), is in the process for piloting or introduction in Angola, Bangladesh, Benin, Burkina Faso, Cameroon, Côte d’Ivoire, Democratic Republic of the Congo, Kenya, Laos, Madagascar, Malawi, Mozambique, Myanmar, Niger, Nigeria, Senegal, Uganda, and Zambia (9, 10). The simpler delivery mechanism facilitates the provision of this method by community health workers and potentially by self-injection, which could have important implications for reaching underserved populations with contraceptive services. Thus, it is possible that DMPA usage may increase worldwide, especially in economically developing countries.

Sub-Saharan Africa is also the region that bears the greatest burden of HIV-1 infections and AIDS. In 2015, there were ∼19 million people living with HIV in eastern and southern Africa (11). Globally there were ∼1.8 million new HIV infections in 2015, with 75.4% of these occurring in sub-Saharan Africa (12). Women account for more than half of new infections (11). Although the global annual HIV incidence has stayed relatively constant at ∼2.6 million per year since 2005, 74 countries have experienced increases in age-standardized rates of new HIV infections between 2005 and 2015, including several in sub-Saharan Africa, as well as in the Philippines, Cambodia, Mexico, and Russia (12). South Africa has the greatest worldwide HIV-1 prevalence with ∼7 million HIV-1–infected people in 2015 (13, 14), as well as the greatest number of new infections, with an estimated 529,000 new HIV infections in 2015 (12). An important global health success in the past 10 years has been the scale-up of antiretroviral therapy to prevent mother-to-child transmission, and the expansion of antiretroviral therapy, resulting in an increase in the number of people living with HIV from 28 million in 2000 to 39 million in 2015 (12).

Geographically, areas with the highest usage of injectable contraceptives overlap with those with the highest HIV-1 prevalence (15). A recent meta-analysis of epidemiological data, involving tens of thousands of women, suggests that DMPA-IM usage may be associated with a 40% (range, 23% to 59%, with 95% confidence) increase in the risk of HIV-1 acquisition (4). This estimate is consistent with 2015 meta-analyses (16, 17), enhancing concerns about the potential causal relationship between DMPA-IM use and HIV-1 acquisition risk. The results suggest that an increase in HIV-1 acquisition may depend on the specific type of HC because data for NET-EN, COCs, and levonorgestrel (LNG) implants collectively suggest no significantly increased risk of HIV-1 acquisition (4), although data are somewhat limited for NET-EN and very limited for implants. Insufficient data are available to assess the effects of other forms of contraception, including DMPA-SC, contraceptive patches, rings, or hormonal IUDs, on HIV-1 acquisition (4). It has been argued that the observed association with HIV-1 acquisition for DMPA-IM may be confounded by differential condom usage between women using DMPA vs women in the comparison groups, although recent data suggest this is unlikely to be the case (4, 16, 18–21).

In response to the accumulated epidemiological evidence and rising concerns, the World Health Organization has recently changed the medical eligibility criteria for injectable contraceptives, including DMPA-IM, DMPA-SC, and NET-EN, to category 2 (22) (indicating that the benefits of the method generally outweigh the theoretical or proven risks). The new guidance specifically emphasizes that women considering using these methods should be advised about the potential relationship with HIV risk. Furthermore, a multicenter, open-label, randomized clinical trial comparing HIV incidence and contraceptive benefits in women using DMPA-IM, an LNG implant, and copper IUDs (Cu-IUDs), conducted by the Evidence for Contraceptive Options and HIV Outcomes consortium (23), began in 2015, with results expected in 2019. Obtaining more insight into the effects of different progestins and the biological effects of HC on HIV-1 acquisition represents a global public health priority.

## Contraceptives: Actions, Types, and Serum Concentrations

Progestins are compounds with progestational activity, in particular the capability to induce a secretory endometrium to support gestation (24). The progestin component of contraceptives is designed to mimic the actions of progesterone, the endogenous progestogenic steroid hormone in all mammals. Progesterone plays a central role in reproduction to regulate establishment and maintenance of pregnancy, normal mammary gland development, menstrual cycle, and sexual behavior. During the follicular phase of the menstrual cycle, progesterone levels are relatively low whereas estrogen levels peak. Levels of progesterone, secreted by the corpus luteum, rise after ovulation to prepare the endometrium for egg implantation, transform estrogen-primed endometrium into secretory tissue, prevent ovulation by abolishing peak gonadotropin levels, inhibit follicular maturation, and thin the endometrium. Higher progesterone levels also reduce glycogen secretion and cause cervical mucus to become thick and viscous to avoid sperm penetration, fertilization, and implantation by multiple embryos. Progestins mimic all of these actions of progesterone to prevent pregnancy via these multiple mechanisms. Estrogen, alternatively, induces gonadotropin release and ovulation in addition to having many additional beneficial effects on brain and cardiovascular function, lipid profiles, and bone density (25, 26).

A wide range of HCs exists, including oral pills, injectables, implants, rings, patches, and IUDs (27). These vary in the type and dose of a progestogenic compound, as well as the method and frequency of administration. Progestins are either combined with estrogen in contraceptive formulations for better menstrual cycle control or used alone as progestin-only contraceptives. Progestins, all synthetic progestogenic steroids, used for HC in sub-Saharan Africa are MPA (which is structurally related to progesterone) and NET-EN, etonogestrel (ETG), and LNG (which are structurally related to testosterone) (24). Progestin-only injectable contraception is a highly effective, reversible contraceptive method with some negative transient effects on bone mineral density and potentially on weight gain (26, 28). DMPA-SC and DMPA-IM appear to be therapeutically equivalent regarding contraceptive efficacy, as well as side effects on bone density and weight gain (26, 29). Long-term highly efficient and reversible methods of contraception include ETG-releasing subdermal implants (Implanon) and vaginal rings (NuvaRing), and LNG-releasing implants (Jadelle, Sino-implant) and IUDs (Mirena, Skyla, Liletta) (27). In contrast to progestins, the estrogenic component of contraceptives, if present, does not vary as much in dose and composition, and usually contains low-dose ethinyl estradiol (EE) to minimize venous thrombosis and cardiovascular risk. Newer generation COCs contain natural compounds such as estradiol (E2) or estradiol valerate to minimize metabolic effects and decrease the thrombotic risk of formulations with EE (30). Most current COCs contain the progestins LNG, ETG, nomegestrol acetate, or drospirenone (27). Although several clinical studies on COCs and HIV-1 acquisition fail to mention the progestin or estrogenic components, most COCs used by women in these studies in the developing world contain LNG (16). Similarly, many studies do not discriminate between DMPA-IM and NET-EN and refer to them collectively as injectables. Of greater concern is that many research articles use the term “progesterone” to refer to progestins such as MPA. The concept that progestins have different properties compared with progesterone and each other and therefore cannot be considered as a single class of compounds is gaining recognition among different disciplines (24, 31, 32). Analytical recommendations for studies assessing the relationship between HC methods and the risk of HIV acquisition suggest disaggregation by specific method type and hormonal content (33).

A critical issue regarding the effects of HC on HIV-1 acquisition is the question of doses and concentrations, in particular for MPA. It is well known that high doses of ∼1000 mg per day of MPA used to treat patients who have cancer are strongly immunosuppressive, resulting in, among other effects, the inhibition of lymphocyte proliferation and activity (34, 35). Furthermore, MPA is widely used to facilitate infection of sexually transmitted diseases in mouse and monkey experimental models, including of simian immunodeficiency virus (SIV) in monkeys (36, 37). The mechanisms underlying this increased susceptibility are discussed in more detail in subsequent sections. The critical question is thus: if MPA is immunosuppressive at high doses in humans and increases the risk of viral infections in animal models, do these effects occur in humans at lower doses? Therefore, serum concentration and the relationship to biological responses that affect HIV-1 acquisition and pathogenesis become critical issues for MPA.

“Different concentrations of free progestins are likely to play a key role…including on HIV-1 acquisition.”

Serum concentrations of progestins vary widely for different types of contraception, both in terms of peak levels shortly after administration, and as a function of time before the next dose. The progestin concentration at a particular time in the serum of contraceptive users that is available to exert a physiological response on cells and tissues depends on many factors, including the type of progestin, dose, method of administration, metabolism, and pharmacokinetics (24, 25, 38, 39). Additionally, individual patient physiology, health status, and body type affect metabolism and pharmacokinetics. The relative affinity of the progestin for serum proteins that carry the steroids in the blood, as well as serum protein concentrations and the concentrations of competing endogenous steroids, will codetermine the amount of pharmacologically active “free” progestin (24, 25, 40). Progestin serum concentrations can also vary greatly between individuals on the same contraceptive (41–47). These different concentrations of free progestins are likely to play a key role in the determination of differential side-effects, including on HIV-1 acquisition.

Serum concentrations of progestins commonly used in HC are compared in Table 1 (41, 42, 46–73). Peak serum concentrations (*C*_max_) after injection are relatively high for the injectable contraceptives (DMPA-IM and NET-EN), as well as for LNG-containing COCs. Values vary widely between reports. For DMPA-IM, peak serum levels are reached within the first 20 days, with reported levels ranging from 3 to 100 nM (1.15 to 38.5 ng/mL), with an average of ∼21 nM (8 ng/mL) (46–59), after which the levels slowly decline to reach a plateau of ∼2.6 nM (1 ng/mL), which is maintained for about 3 months (60). About half of these studies, which span ∼40 years, report average *C*_max_ values between 6 and 12 nM (2.3 to 4.6 ng/mL) (46, 52, 54, 56, 58, 74) whereas the other half report average *C*_max_ values in the range 13 to 62 nM (5 to 24 ng/mL) (48, 50, 51, 53, 55, 59, 68). There is no trend showing that only older studies (20 to 40 years old) report higher *C*_max_ values or only more recent studies (up to 20 years old) report lower *C*_max_ values. It is not possible to deduce from these data (51, 75–77) whether differences in *C*_max_ values reported for DMPA-IM between studies are due to differences in detection methodology, number of injections, time of measurement after injection, or intrinsic factors of the study group such as interindividual differences in metabolism, lactation, body weight, size, or ethnicity. Furthermore, within one study, serum concentrations at both peak and plateau can vary between individuals by as much as 10-fold or 38-fold (45, 47). Given the wide range of reported peak serum levels for DMPA-IM, and the fact that peak serum levels for DMPA-IM and DMPA-SC have not been compared in parallel in the same study, it is difficult to assess how they vary for the two methods. Peak serum levels for DMPA-SC have been reported in the range 1.6 to 4.4 nM (62, 63), which appears to be lower than for DMPA-IM, but plateau concentrations (C30, see Table 1) and contraceptive efficacy appear to be similar (62, 61). MPA used therapeutically to treat breast and endometrial cancers by ingestion or intramuscular injection of ∼1000 mg daily results in serum concentrations of 26 to 2030 nM for oral administration (78) or 251 to 422 nM by intramuscular injection (79), resulting in endocrine disruption and immunosuppression (35, 80, 81). Lower oral doses of MPA result in serum concentrations of ∼15 to 120 nM for 100 mg (82) and ∼100 to 420 nM for 200 mg (83).

**Table 1.** **Systemic Concentrations of Progestins**

	***C*_max_ (nM)**	***C*_max_ (ng/mL)**	***C*_30 d_ (nM)**	***C*_30 d_ (ng/mL)**	***C*_1 y_ (nM)**	***C*_1 y_ (ng/mL)**	**Reference**
DMPA-IM, Depo-Provera*^a^*	21 (3–100)	8 (1.15–38.5)	2.6	1	—	—	(46–61)
DMPA-SC, Sayana Press*^b^*	3.3 (1.6–4.4)	1.3 (0.6–1.7)	2.1	0.8	—	—	(61–64)
LNG-IUD*^c^*	0.3–2.4	0.1–0.78	—	—	0.4–0.9	0.13–0.29	(41, 65)
LNG implant*^d^*	1–11	0.32–3.57					(66)
LNG COC*^e^*	28	9.1	—	—	—	—	(67)
NET/NET acetate*^f^*	10–50	4.1–20.6	0.6–10	0.24–4.1	—	—	(52, 68–70)
ETG/3-ketodesogestrel IUD*^g^*	3–4.6	0.9–1.4	—	—	—	—	(42)
ETG/3-ketodesogestrel implant*^h^*	2.5–2.8	0.78–0.87	—	—	0.8–1.2	0.25– 0.37	(71, 72)
ETG/3-ketodesogestrel COC*^i^*	5.5–7.5	1.7–2.3	—	—	—	—	(73)

The table summarizes approximate systemic (plasma) concentrations of progestins. High variability exists between individual users and between studies (see “Contraceptives: Actions, Types, and Serum Concentrations). Inconsistency between studies may reflect differences in experimental techniques and methods of determination of progestin concentration. *C*_max_ indicates the maximum concentration (typically reached within the first 20 days after administration). C_30 d_ and C_1 y_ indicate data obtained at ∼30 days and ∼1 year after administration (time points differ between studies). *C*_max_ concentrations of plasma MPA after DMPA-IM and DMPA-SC administration have not been compared in a head-to-head study, but they have been at 6 months to 2 years, where levels were measured just before the next injection (61).

^*a*^ Intramuscular injection of 150 mg of MPA every 3 months.

^*b*^ Subcutaneous injection of 104 mg of MPA every 3 months.

^*c*^ Mirena IUD, 52 mg of LNG; levels decrease to ∼0.4 to 0.9 nM at 1 year.

^*d*^ LNG implants: Norplant, 36 mg, 5 years; Jadelle, 75 mg, 3 years.

^*e*^ LNG COC, typically 50 to 150 µg of LNG per pill in combination with ethinylestradiol.

^*f*^ Intramuscular injection of 200 mg of NET-EN every 2 months.

^*g*^ NuvaRing (intravaginal ring), 11.7 mg of ETG.

^*h*^ Implanon, 68 mg of ETG; Nexplanon, 68 mg of ETG.

^*i*^ ETG COC, typically 150 µg of ETG per pill.

Regarding the immunosuppressive effects of MPA, the question is raised as to what the cut-off concentration is, below which general or selected and cell-specific immunosuppression no longer occurs, and whether this is the same for different individuals and different immunosuppressive responses. Regarding other progestins, LNG is widely used in intravaginal devices, implants, and COCs and in multipurpose prevention technologies in development (84), with serum concentrations ranging from 0.3 (0.1 ng/mL) to 28 nM (8.7 ng/mL). ETG, also known as 3-ketodesogestrel, is a metabolite of desogestrel and is also administered in intravaginal devices, implants, and COCs, with serum concentrations ranging from 0.8 nM (0.26 ng/mL) to 7.5 nM (2.4 ng/mL) (71, 72). These ranges for LNG and ETG depend on the type of HC and time after administration.

It is interesting to compare the different serum concentrations of progestins to those of endogenous progesterone. The concentration of endogenous progesterone in the serum of premenopausal women is low during the follicular phase (0.4 to 1.6 nM) (85) but rises to ∼16 to 51 nM during the luteal phase, and to ∼600 nM during pregnancy (25, 85–87). However, it is difficult to predict how responses would differ for equivalent serum concentrations of progesterone as compared with progestins because multiple factors such as binding to serum proteins, pharmacokinetics, affinities, potencies, and efficacies via steroid receptors will affect the responses, as discussed in subsequent sections.

## Intracellular Mechanisms of Action of Progestins

Central to understanding the effects of a particular progestin on the acquisition of HIV-1 and other STIs *in vivo* is an appreciation of what the steroid does inside different cell types in target tissues. Note that no information is available about tissue concentrations of progestins, which may be different from serum concentrations. Once the progestin reaches the cells at a particular concentration, it is predominantly the intracellular actions of the progestin, the concentration of steroid receptors, the concentration of competing steroids, the concentration of other signaling molecules, and the cell type that determines the biological response inside the cell (24, 25, 88).

**Figure 1.**
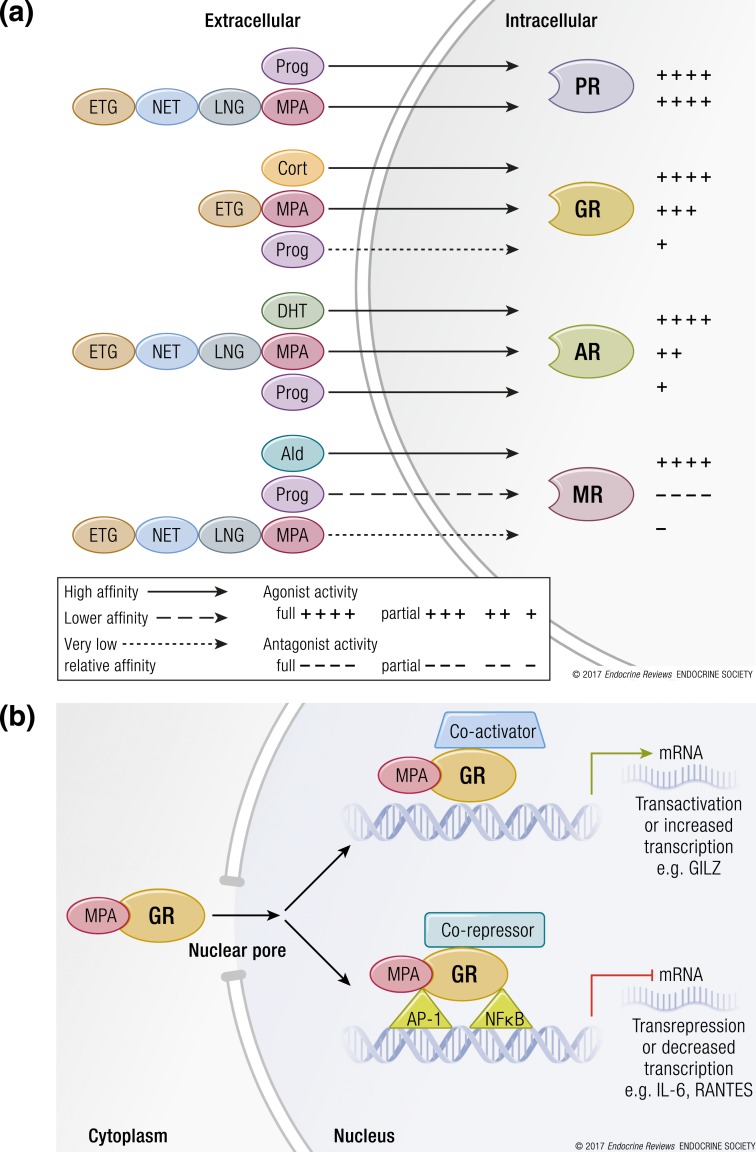
Schematic diagram illustrating steroid receptor selectivity of progestins and intracellular mechanism of regulation of transcription by steroid receptors. (a) Binding and transcriptional activity. Solid black lines indicate high-affinity binding similar to or greater than that of the endogenous cognate agonist ligands. These are progesterone (Prog) for the PR, cortisol (Cort) for the GR, dihydrotestosterone (DHT) for the AR, and aldosterone (Ald) for the MR. Stippled black line indicates lower affinity (∼10-fold lower), whereas dotted black lines indicate very low relative affinity (at least 100-fold lower) compared with the endogenous agonist ligands. Plus signs (+) indicate agonist or partial agonist activity, where four plus signs (++++) indicate full agonist activity similar to endogenous agonist ligands and fewer plus signs indicate relative partial agonist activity. Minus signs (−) indicate antagonist activity toward the endogenous agonist, where four minus signs (−−−−) indicate full antagonist activity and fewer minus signs indicate relative partial antagonist activity. Data sources include references listed in Tables 1 and 2 and references discussed in the text. Note that there is considerable uncertainly and/or inconsistency in some of the data as discussed in the text, especially for affinities and transcriptional activities of LNG and ETG for GR, AR, and MR. Additionally, steroid receptor activity can be gene and cell specific, and many of the activities in animal preclinical models may not represent activities in human tissues. (b) Mechanisms of transcriptional regulation by MPA acting via the GR. Other progestin agonists and partial agonists can both transactivate and transrepress genes via their cognate receptors using similar mechanisms. Green arrows indicate increase in transcription of target genes (transactivation by direct binding of receptor to DNA) whereas red stop lines indicate inhibition (transrepression by tethering of receptor to other transcription factors), both taking place in the nucleus. Chromatin effects are not indicated. Antagonist activity is not depicted but involves recruitment of corepressors instead of coactivators by DNA-bound receptors, due to the conformation induced by the antagonist bound to the receptor. Additional mechanisms of gene regulation may be involved. Ald, aldosterone; Cort, cortisol; DHT, dihydrotestosterone; Prog, progesterone. With data from Stanczyk *et al.* (24) and Africander *et al.* (25).

Progestins are designed to act similar to progesterone and to have similar progestogenic actions. Progestins and progesterone are steroids that act predominantly via changing gene expression after binding to and activating intracellular steroid receptors [Fig. 1(a) and 1(b)]. These receptors generally have minimal activity in the absence of the steroid, although some ligand-independent effects do occur (89, 90). Progestins have a high affinity for and are designed to act via the progesterone receptor (PR). The PR regulates gene expression via mechanisms common to all the steroid receptors. Other steroid receptors include the glucocorticoid (GC), androgen, mineralocorticoid, and estrogen receptors (GR, AR, MR, and ER, respectively). Steroid receptors can either stimulate or repress gene expression by increasing (transactivation) or decreasing (transrepression) the rate of transcription initiation [Fig. 1(b)]. The best described mechanism of transactivation occurs via direct binding of the activated steroid receptor to specific DNA sequences in the regulatory regions of target genes, whereas the best described mechanism of transrepression occurs via tethering of the steroid receptor to other transcription factors bound to those regulatory regions to change the rate of transcription initiation [Fig. 1(b)]. However, note that several other mechanisms have been described, including upregulation by tethering (91, 92). Mechanisms of transrepression have been more extensively studied for the GR than for the PR. This is largely due to the pharmacological relevance of GCs in the treatment of most inflammatory diseases. The immunosuppressive activity of GCs is largely due to their ability to repress transcription of genes that are activated by the proinflammatory transcription factors activator protein 1 and nuclear factor *κ*B (NF-*κ*B), by the tethering mechanism, as illustrated in Fig. 1(b). Regulation of transcription by steroid receptors is a complex process involving multiple proteins, including RNA polymerase and associated factors, cofactors, and chromatin modifying proteins, to form multiprotein complexes on the target genes. Some of these proteins can change the chromatin structure and reprogram the cell to express a particular repertoire of genes. Steroids are master regulators of total gene expression in cells because each cell has hundreds or even thousands of target genes for a particular steroid receptor. Notably, steroid responses are cell and gene specific and are also dependent on crosstalk with other signaling pathways (89).

**Figure 2.**
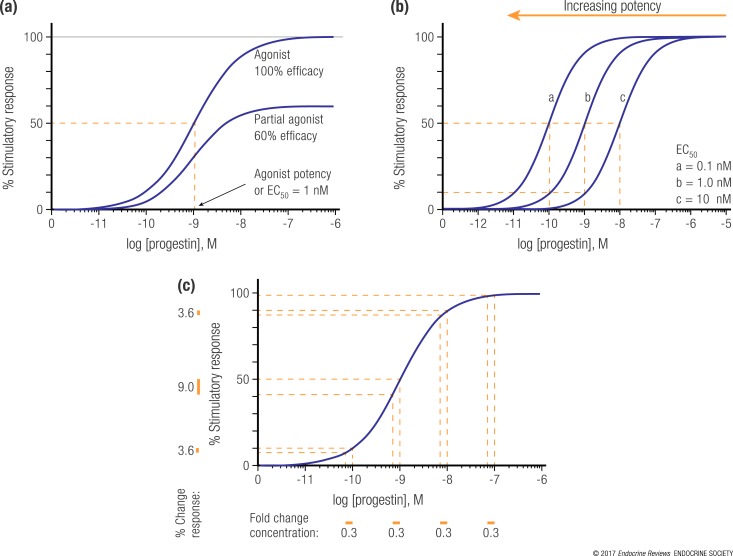
Typical sigmoidal dose–response curves for progestins acting via steroid receptors. (a) Curves depicting a stimulatory response (such as transactivation) for a full agonist (100% efficacy) and a partial agonist (60% efficacy). The potency, or the concentration of steroid for half-maximal response (EC_50_ value), of 1 nM is indicated for the full agonist by a dotted orange line. (b) Curves depict three separate stimulatory responses with potencies or EC_50_ values of 0.1, 1, or 10 nM (a, b, and c, respectively). An orange arrow illustrates how the curves shift to the left as the EC_50_ decreases and potency increases, which occurs with increased progestin steroid receptor concentration. (c) The curve shows a stimulatory response for an agonist with orange dotted lines to indicate how the percentage point response changes as the concentration of progestin changes by 0.3-fold, for different parts of the curve. (a–c) The slopes of the sigmoidal curves are set at 1, assuming one steroid ligand binding reversibly to one receptor, the absence of cooperative effects on ligand binding, and no spare receptors.

Surprisingly, we do not know the precise target genes responsible for the progestogenic actions of progesterone. However, what is known from microarray and RNA sequencing and chromatin immunoprecipitation sequencing analysis of all the genes affected by progestins in a single cell type is that progestins regulate hundreds of genes involved in multiple cellular functions in target cells (93–96). Furthermore, gene expression profiles in response to progestins vary for different cells, can be modulated by the presence of other hormones and transcription factors, and vary between progesterone and other progestins (93, 96–99). Given that progestins have progestogenic actions via the PR similar to progesterone, why might they exhibit different biological effects compared with progesterone, and to each other? Key to understanding this is an appreciation of the differential effects of progestins via other members of the steroid receptor family of proteins. Because the PR, AR, GR, and MR are structurally related in the steroid-binding site, several progestins bind to varying degrees to other members of the steroid receptor family of proteins besides the PR. These relative binding affinities of some progestins are summarized in Table 2 (24, 25, 32, 38, 88, 100–104) and illustrated in Fig. 1(a). The extent of this crosstalk varies because the different progestins have different relative binding affinities for the GR, MR, and AR. Although progestins do not appear to bind to the ER, NET has been shown to be aromatized in women to the potent estrogen EE (39, 105–108), which binds to the ER. All progestins exhibit some binding for the AR. MPA has a relatively high affinity for binding to the GR, whereas progesterone and ETG bind to the GR with much lower affinity. LNG and NET exhibit almost no GR binding. Progesterone has a relatively high affinity for the MR, whereas the affinity of LNG is uncertain and MPA, ETG, and NET exhibit very low affinity for MR binding. All the progestins have a high affinity for the PR, which is higher than that of progesterone. Thus, in terms of binding to different steroid receptors, MPA is clearly an outlier among this group of progestins, with a relatively high affinity for the GR, unlike progesterone and NET (equilibrium dissociation constants for inhibition, or *K*_i_ values, of 10.8, 270, and 215 nM, respectively) (103), whereas data for ETG and LNG are not widely available. The implications of this *K*_i_ or *K*_d_ value for MPA are that 15.6%, 31.6%, and 48.0% of GR will be occupied at 2, 5, and 10 nM MPA, respectively [*K*_i_ and *K*_d_ are equivalent. *K*_i_ is often used instead of *K*_d_ when the constant is determined using competitive binding assays. The equilibrium dissociation constant, or *K*_d_, is approximately the concentration of steroid at which 50% of receptors are occupied and is useful in determination of fractional occupancy, or the percentage of receptor bound by steroid. Fractional occupancy (Y) depends on *K*_d_ and steroid concentration according to the equation Y = [L]/(*K*_d_ + [L]), where [L] indicates ligand or steroid concentration. Note that as receptor concentration increases a greater response will occur for the same fractional occupancy, because the number or receptors occupied will determine the extent of the response (see Fig. 2c)]. The presence of different isoforms of steroid receptors, such as PR-A and PR-B, has the potential to discriminate further between biological effects of different progestins (99), although not much information is available regarding progestins.

**Table 2.** **Relative Binding Affinities for Steroid Receptors and Biological Activities of Progestogen**

**Progestogen**	**PR**		**AR**			**GR**		**MR**		**ER**		
	**RBA %**	**Progesto-genic**	**RBA %**	**Andro-genic**	**Antiandro-genic**	**RBA %**	**Gluco-corticoid**	**RBA %**	**Antimineralo-corticoid**	**RBA %**	**Estrogenic**	**Antiestro-genic**
Progesterone	100	+	80	±	(+)	5	±	6	+	—	−	+
MPA	65–98	++	151	±	−	74	+	0.13	±	—	−	+
LNG	23–96	++	58	+	−	1–7.5	−	17–75	±	—	−	+
NET/NET acetate	27–34	++	134	+	−	0.8	−	0.15	±	—	+	+
ETG/3-ketodesogestrel	150	++	20	(+)	−	14	±	<0.1	−	—	−	+

Relative binding affinities are expressed as a percentage relative to 100% for reference steroids, which were as follows: PR, progesterone; AR, dihydrotestosterone; GR, dexamethasone; MR, aldosterone, as discussed in (88). Relative binding affinity and cell model biological activity data for GR, MR, and AR for progesterone, MPA, and NET acetate are from (100–104). All other relative binding affinities and all other progestin biological activities in preclinical models were taken from references (32, 38). Biological activities determined in animals (mostly rats and rabbits) used a range of assays as reviewed in (88). Sources of data include references (24, 25). −, not effective; (+), weakly effective; +, effective; ++, strongly effective; ±, literature inconsistent.

Abbreviation: RBA, relative binding affinity.

Thus, MPA has the capability of exerting different biological effects compared with progesterone, LNG, and NET, in particular via the GR (Fig. 1). However, affinity is not proportional to biological activity. The affinity will determine what percentage of a receptor is occupied by a steroid, but the response depends on the conformation of the receptor that is induced by the steroid to enable it to interact more or less efficiently with other proteins involved in the intracellular pathway and on the target genes, as well as the receptor concentration. To investigate the type of response and the relationship between concentration and response elicited by a progestin in a cell, dose–response analysis is required.

## Biocharacter and Dose Response to Progestins

### Relevance to biological effects

A biological dose–response analysis reveals how a biological effect changes as the dose of a progestin changes (88). Some dose–response studies have used cells that express more than one receptor, confounding the results, or direct head-to-head comparisons between effects of progestins have not been made by performing experiments in the same model cell system in parallel (24, 25, 88). Other researchers have circumvented this problem using models expressing only a single receptor, or by use of steroid-specific antagonist or receptor knockdown, to show that a response is via a particular receptor, although these types of data have been published for only a few selected progestins (24, 25, 100–104). These data can give the most reliable measure of affinity, potency, efficacy, and biocharacter for a progestin via a specific steroid receptor or for a measured response. If a dose response is mediated by a steroid receptor, the curve will typically be sigmoidal-shaped when plotting a response to a steroid on the *y*-axis vs the log of the steroid on the *x*-axis (Fig. 2). This has been demonstrated for progestins regulating gene expression in cell models expressing only a single member of the steroid receptor family (100–103, 109–112). The shape of the curve and slope of the steep part of this curve are determined by the laws of mass action, usually resulting in a slope of unity (Hill slope) when a single steroid molecule binds to a single receptor molecule (113). The 50% effective concentration (EC_50_), or potency, is defined as the concentration of the steroid required for half of the maximal response. A change from 10% response to 90% response spans a range of concentrations on the *x*-axis of ∼100-fold (*e.g.*, say from 0.1 to 10 nM when the EC_50_ is 1 nM), corresponding to the steep part of the curve [indicated with orange brackets in Fig. 2(a)]. The maximum possible response is referred to pharmacologically as the efficacy, which is set at 100% for an agonist. The EC_50_ occurs at the steepest part of the curve and is the concentration around which the greatest change in response will be observed with changes in the steroid concentration (Fig. 2). Additionally, it is clear that if the dose of a steroid changes, but falls within the concentration range of the bottom or top plateau of the dose–response curve, no difference in response will be detected, unlike when the dose change falls within the steep part of the curve. A progestin is defined as relatively potent when a low concentration is required to induce a response, usually relative to a reference steroid agonist, such as the synthetic agonist R5020 for the PR or dexamethasone for the GR. For example, dose–response analysis has revealed that MPA has a potency ∼500-fold greater than progesterone for regulation of transcription via the NF-*κ*B transcription factor, with efficacies of 87% vs 30% for MPA vs progesterone, relative to dexamethasone via the GR, in a cell line model (100). The greatest change in response to MPA occurs with changes in MPA concentration of ∼1 nM, that is, the EC_50_ of MPA in this particular model system. In contrast, NET shows no response at concentrations <10 µM (100).

Whether a progestin is an agonist, a partial agonist, or an antagonist, known as its “biocharacter,” is a measure of the extent of the maximum response possible relative to a reference agonist. This can also be determined from dose–response curves, as illustrated in Fig. 2(a) for agonist and partial agonist. All progestins are agonists for the PR. Progestins can also be steroid receptor antagonists, that is, they can bind to steroid receptors other than the PR but induce a nonoptimal receptor conformation and hence result in no change in a response on their own, but they can block the effect of an agonist. Antagonist potency and efficacy are determined by dose–response analysis using a constant concentration of the agonist in the presence of varying concentrations of antagonist, as was used to show potent antagonist activity for progesterone acting via the MR (101). Thus high-affinity binding does not necessarily confer a high agonist or partial agonist response. An antagonist such as RU486 binds both the GR and PR with a relatively high affinity, but it is an antagonist for both receptors. Several progestins can act as partial or full agonists for receptors other than the PR. For example, both MPA and NET can act as agonists for the AR, with progesterone acting as a partial agonist (102), whereas MPA can act as a partial agonist or full agonist for the GR, depending on cell and gene context. In contrast, progesterone is a partial agonist for the GR with low potency, whereas NET has no GR agonist activity in cell line models (100). Because steroids can either increase or decrease responses such as gene expression, dose–response curves can either be stimulatory (as for transactivation), depicted in Fig. 2(a), or inhibitory (as for transrepression). MPA has been shown to be a relatively potent full agonist for transrepression and a partial agonist for transactivation via the GR, whereas progesterone is a relatively weak, partial agonist for both via the GR (100, 103, 114, 115). Biocharacters can also be cell specific and sometimes even gene specific, such as for many synthetic selective steroid modulators, similar to tamoxifen via the ER. Importantly, the EC_50_ can vary depending on the progestin receptor concentration inside the cell. Fig. 2(b) shows how potency could increase from 10 nM (curve c), to 1 nM (curve b), to 0.1 nM (curve a) just by increasing the receptor concentration in the same cell [Fig. 2(b), low receptor concentration for curve c and high receptor concentration for curve a]. The effects of changing receptor concentrations have been best demonstrated for the GR, and they have also been shown to switch the biocharacter of MPA from a partial agonist to a full agonist via the GR (89, 116, 117). EC_50_ also varies with the concentrations and interactions of proteins and other molecules that mediate the response downstream of steroid binding, as well as the affinity of the progestin for its receptor. Hence, progestin potency cannot be predicted and is steroid, cell, and even gene specific.

Dose responses to progestins can be measured in patients, animals, tissue, and cells in culture, and they can include any response of interest such as inhibition of ovulation, expression of specific genes and proteins, apoptosis, and HIV-1 replication. However, very few such analyses have been performed for different progestins (88). Nevertheless, additional clues regarding the steroid receptor activity of progestins have been obtained from preclinical animal studies that monitor typical but selected physiological effects associated with an endogenous steroid (24, 38). However, inconsistent terminology, such as using the term potency when referring to efficacy, has complicated interpretation of the contraception literature (88). Such preclinical data do not provide proof of direct effects via a specific receptor, although they give useful information of physiological relevance. For example, “estrogenic” activity for a particular progestin obtained in animal models does not prove a direct effect of the progestin via that receptor (*e.g.*, the ER) because effects can be indirect. Estrogenic effects could be due to the progestin acting via another receptor such as the PR to regulate expression of other genes that then affect estrogen synthesis or metabolism, or ER expression levels, which can subsequently affect estrogen responses. Additionally, full dose–response curves are seldom generated in preclinical studies, so the potency and efficacy are not known in most cases. Data from preclinical models are summarized in Table 2, whereas dose–response data from cell line models are discussed later. With the exception of estrogenic activity, the binding, cell line dose–response, and preclinical animal model data are remarkably consistent. For example, the preclinical animal data confirm the general androgenic activity of MPA, LNG, and NET and the GC activity of MPA but not NET and LNG, as well as the anti-mineralocorticoid activity of progesterone but not MPA or NET, without dose–response analysis to define biocharacter more precisely.

Despite the limited data as later discussed and shown in Tables 1 and 2, important insights have been obtained from the evidence to date. Potency, efficacy, and biocharacter are highly relevant to dosage, concentrations, and contraceptive efficacy of progestins, as well as side effects such as effects on intracellular actions that affect HIV-1 acquisition. Substantial data show that MPA is a relatively potent partial to full agonist via the GR, whereas progesterone is a very weak partial GR agonist, and NET exhibits almost no GR activity. The potency for progesterone is ∼100- to 800-fold lower than MPA via the GR, and its efficacy is ∼5% to 20%, depending on the promoter (100). Fewer data are available for ETG and LNG, but preclinical results to date suggest that ETG has weak GC activity and LNG has almost no GR activity. In contrast, all of the previous progestins have some androgenic activity. Progesterone has potent anti-mineralocorticoid activity, unlike MPA, ETG, and NET, whereas LNG has some anti-MR activity. Agonist activity of the different progestins via the PR, however, appears to be similar, although the progestins are usually more potent than progesterone (24, 118). Thus, it can be seen that none of the progestins acts exactly the same as progesterone by the previous criteria, whereas MPA, in particular, appears to be an outlier, with not only a relatively high affinity for the GR but also substantial GC-like activity at nanomolar concentrations. Such off-target differential actions of progestins via steroid receptors other than the PR are relevant if any of these other receptors could cause biological effects that would increase HIV-1 acquisition or pathogenesis, in response to the progestins, at concentrations found in women.

### Relevance of contraceptive doses to acquisition of HIV-1 and other STIs

Dose–response curves have not been determined for most progestins in clinical settings or in animals. Thus, thresholds for contraceptive efficacy are mostly poorly defined or undefined (40). It is apparent that the 150-mg dose of DMPA-IM is more than needed for a maximal response for inhibition of ovulation because a dose reduced by 31%, as in DMPA-SC, still inhibits ovulation (46). Thus, both of these doses would fall at 100% response of a dose–response curve (Fig. 2) if plotted for the inhibition of ovulation. It is tempting to speculate that lowering the dose of DMPA-IM by 31% to that of DMPA-SC, still adequate for contraceptive efficacy, will similarly reduce possible side-effects of higher doses (62, 119), but based on dose–response considerations this is unlikely. Lowering the dose by 31% is only likely to lower the theoretical response by at most about 9%, which occurs when the original dose is around the EC_50_ [Fig. 2(c)]. This is consistent with published dose–response data for gene expression where sufficient points have been used to define the top and bottom and slope of the curve with reasonable accuracy (100–103, 109–112). It is possible that the shape and slope of a dose–response curve may be different for other biological responses and for *in vivo* effects, where the maximal change in response with changes in dose around the EC_50_ could be >9% or <9%. However, additional data are required to investigate this issue. Substantial *ex vivo* data exist for MPA on the relationship between MPA concentrations and various responses relevant to immune function and HIV and STI acquisition, whereas limited data are available for other progestins and progesterone, as discussed later.

MPA was shown decades ago to bind to the GR with a higher affinity than cortisol, the endogenous GC in humans (120, 121), and to have relatively potent GC activity in repressing the proinflammatory interleukin (IL)-2 gene with a potency of ∼100 nM (114), with both studies having been done in primary human lymphocytes. As early as 2004, MPA was shown to repress the proinflammatory IL-8 gene via the GR in a cell line model at 2.6 nM, the plateau concentration of MPA in DMPA-IM users (104). Since then, more detailed dose–response analyses in various cell line and primary human cell models have revealed that MPA represses many cytokine and chemokine genes or promoters containing activator protein 1 or NF-*κ*B transcription factor binding sites via the tethering model (Fig. 1), with potencies (EC_50_s) usually ranging from 1 to 100 nM, in a gene- and cell-specific manner, acting as a partial to full GR agonist via the GR (100, 103, 115, 122, 123) (see previous “Relevance of biological effects” and Fig. 2). These ranges in potencies most likely reflect differences in GR levels and promoter- and cell-specific factors, as previously discussed in “Intracellular Mechanisms of Action of Progestins” and “Biocharacter and Dose Response to Progestins”. Of particular interest to acquisition of HIV-1 and other STIs, MPA has been shown to have a potency of ∼3 to 20 nM in changing expression of several immune function genes in cervical cell lines (109, 115), although for some cell line models the potency appears to be much lower (124). In primary peripheral blood mononuclear cells (PBMCs), potencies of ∼10 to 100 nM have been reported for changes in expression of immune function genes (112, 122, 123, 125, 126), with significant effects being observed on expression of some genes at 10 nM MPA (122, 123, 126). In some studies, dose responses of other progestins or progesterone have been compared with MPA, with results showing no effect by equimolar NET (103, 115, 123, 126) and little to no effect by equimolar progesterone (103, 112, 115, 122, 123, 126) on expression of selected genes. For example, no effect was observed on the anti-inflammatory GC-induced leucine zipper (GILZ) messenger RNA (mRNA) with 100 nM progesterone or NET, whereas MPA showed partial agonist activity with an increased mRNA even at 10 nM in an endocervical cell line (115). In the same study, MPA repressed proinflammatory IL-6, IL-8, and regulated on activation, normal T cell expressed and secreted (RANTES) mRNA at 10 to 100 nM, with NET showing no repression even at 10 µM. Similarly, progesterone showed no repression of IL-6 and IL-8 even at 10 µM, but ∼30% repression at 10 nM of RANTES mRNA, albeit less so than MPA (115). Similar results were obtained for MPA, NET, and progesterone on IL-6, IL-8, and GILZ mRNA levels in PBMCs (123) for repression of selected immunomodulatory genes by MPA vs progesterone in BCG-induced PBMCs (112), for MPA vs progesterone on several other proinflammatory cytokines such as interferon (IFN)-*γ* and tumor necrosis factor-alpha (TNF-*α*), in PBMCs and repression of pDCs and T cell function (122, 126). Limited studies on gene expression with LNG show that it has little effect on expression of immune function genes at equimolar concentrations compared with MPA (123, 126), whereas ETG has partial activity (126), consistent with its low potency and partial GR activity. Besides investigating effects on gene expression, different researchers have investigated effects of MPA on various functional properties of immune cells. Results in primary PBMCs show that MPA inhibits some immune cell functions with potencies of ∼0.5 to 1 nM (127) and others with potencies of ∼100 nM (126). MPA, unlike progesterone and NET, increases apoptosis in CD4^+^ T cells with a potency of ∼10 nM (128). Some limited studies have been performed with progestins and HIV in primary cells models, with MPA shown to increase HIV-1 infection in nonactivated PBMCs with a potency of ∼0.1 nM (129) and ∼100 nM for activated PBMCs, unlike equimolar progesterone (122). MPA has also been shown to increase HIV-1 transcytosis on normal epithelial cells with a potency of ∼1 nM, similar to progesterone (130).

Relationships between dose and biological mechanism, as well as effects on HIV and other infections, have also been investigated in mouse and monkey models, mainly for MPA. Some studies in animals have been performed by attempting to use MPA doses comparable to those in women on Depo-Provera (see later “Animal Studies Addressing the Effects of Hormonal Concentration on the Acquisition of Genital Pathogens”). Such studies in mice have shown that MPA impairs mouse defense mechanisms and development of virus-specific immunological memory at ∼100 nM, that is, around the upper limit of detected *C*_max_ serum levels in women on DMPA-IM (131, 132). A more recent study in mice, with measured serum levels of MPA of ∼4 to 7 nM, showed decreased genital barrier function and increased susceptibility to herpes simplex virus (HSV)-1 viral infection (133). Very few researchers have investigated dose–response analysis of progestins in animals where they describe a full dose–response curve. One such study in pigtail macaques (DMPA at 1.5 mg/kg) measured the relationship between vaginal epithelial thickness and dose response and found that the potency for this response was ∼2.6 nM, with an approximately sigmoidal-shaped curve, consistent with receptor theory (134). Another monkey study using a low DMPA dose designed to resemble human contraceptive use found that simian HIV (SHIV) infections in DMPA-treated pigtail macaques were 2.2-fold higher than those of controls (135). However, the authors commented that the study lacked sufficient power to determine whether this effect is significant. MPA serum levels in these monkeys varied from ∼10 ng/mL (about 26 nM) after one injection to ∼20 ng/mL after four injections (∼52 nM). More detailed discussion of the above effects of HC on immune function, susceptibility to infectionsm and their relevance to HIV-1 acquisition follows in subsequent sections.

Taken together, it can be seen from the previous that several studies in animals and *ex vivo* studies have shown that MPA, unlike equimolar progesterone (103, 112, 115, 122, 123, 126), can affect not only immune function (104, 109, 112, 114, 115, 122, 123, 125, 126, 131, 132) but also several other biological effects (128, 130, 133), including HIV-1 replication (122, 129), at concentrations within the range of serum concentrations in DMPA-IM and DMPA-SC users (average spanning ∼1 to 25 nM, and possibly higher for DMPA-IM). The sensitivity of different responses reported above varies in a gene- and cell-specific manner (100, 103, 115, 122, 123), but most responses have potencies (EC_50_s) in the range 10 to 100 nM for MPA, where investigated, with some potencies being even lower (127, 129, 130).

“Differences in anti-MR activity are likely to be substantial for progestins relative to progesterone.”

The consequence of the previous is that DMPA used in injectable contraception, when acting via the GR, is likely to cause effects on immune function and hence on susceptibility to HIV-1 and other STIs, unlike NET, LNG, and luteal phase progesterone concentrations. In target tissues and cells where the GR is the most abundant receptor, very different responses will likely be obtained for MPA as compared with NET and LNG and physiological concentrations of progesterone (excluding pregnancy). Off-target effects in these cells and tissues will most likely be mediated via the GR. GR ligands, or GCs, play a crucial role in most physiological functions, including in maintaining homeostasis, metabolic, bone density, central nervous system, hematopoietic, renal, reproductive, and immune function (89, 92, 136–140). GCs are potent regulators of most aspects of immune function, with predominantly, but not exclusively (92), immunosuppressive effects. GCs regulate all aspects of immune function and inflammation, including immune cell trafficking and differentiation, proliferation, cytokine and chemokine secretion, cytolytic activity, effector function, and antibody production in immune function cells (141). GCs suppress cellular immunity and the production of T helper (Th)1 cytokines, as well as stimulate humoral immunity by enhancing the secretion of Th2-type anti-inflammatory cytokines (89, 136–139, 142). An appropriate immune response relies on a delicate balance between appropriate activation of the immune system and prevention of chronic immune activation by feedback mechanisms. GCs, acting via the GR, are also implicated in playing an important role in HIV-1 pathogenesis and transcription of the HIV-1 promoter or long terminal repeat (LTR) (143).

**Figure 3.**
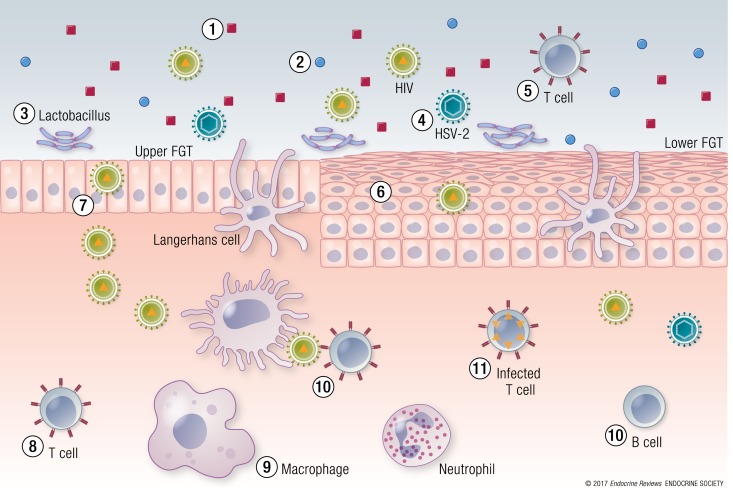
Established biological mechanisms of MPA potentially affecting acquisition of HIV-1 in the FGT. The figure depicts the mechanisms of MPA action for which experimental evidence has been obtained (Tables 3–5). Multiple effects of MPA are consistent with actions via steroid receptors that regulate multiple genes and biological functions. (1) Decreased levels of soluble protective mediators on the mucosal surface, including antimicrobial and antiviral factors and antibodies. (2) Increased secretion of inflammatory mediators that may recruit and activate HIV-1 target cells and immune function cells involved in defense. Decreased expression of some mediators that activate immune function, causing immunosuppression. (3) Changes in the composition of vaginal microbiota and production of soluble factors by bacteria. (4) Increased infection with other viruses such as HSV-2. (5) Increased frequency of HIV-1 target cells in vaginal lumen, including CCR5^+^CD4^+^ T cells. (6) Barrier integrity and epithelial protective function. Decreased expression of proteins involved in barrier integrity, increased barrier permeability. (7) Transcytosis. Increased uptake and transcytosis of HIV-1 via single-layered columnar epithelium of the endocervix and upper FGT. (8) Increased frequency of tissue-resident target cells and elevated CCR5^+^ expression on CD4^+^ T cells. Increased frequency of macrophages and decreased frequency of regulatory T cells in endometrium. (9) Innate immune response. Decreased early innate immune responses in FGT mediated by pDCs. (10) Adaptive immune response. Decreased antigen presentation by professional antigen-presenting cells and decreased induction and maintenance of T and B cell responses. (11) Increased proliferation of HIV-1 in target T cells.

Given the ubiquitous expression of the GR in almost all cells in the body, in contrast to the AR, MR, and PR, MPA could exert GC-like effects on most tissues and cells. Additionally, because steroids are master regulators of hundreds of genes involving multiple cellular processes, taken together with the previous, it is likely that progestins acting via the GR will exert effects on multiple cellular and physiological processes, in multiple cell and tissue types, at concentrations in the range of those found in DMPA users, the extent of which will depend on receptor and target cell MPA concentrations. Because several progestins exhibit androgenic activity, albeit to varying degrees, small differences in progestin actions via the AR are likely to occur. Because most progestins do not bind to the MR, unlike progesterone, which is an MR antagonist, differences in anti-MR activity are likely to be substantial for progestins relative to progesterone. In subsequent sections, we discuss in more depth the present evidence and mechanisms of biological effects of MPA, NET, LNG, and ETG in animal and *ex vivo* models, in the context of susceptibility to HIV-1 and other STIs (Fig. 3; Tables 3, 4, and 5) (36, 37, 109, 112, 115, 122, 125–127, 129–133, 135, 144–174).

**Table 3.** **Summary of *Ex Vivo* Data**

**Reported Effect**	**Progestin**	**Concentration**	**Model**	**Reference**	**Note**
↑ HIV-1 proliferation in PBMCs	MPA	>0.01 nM	Human PBMCs	(129)	
↓ Cytokine production by PBMCs and activated T cells	MPA	>10 nM	Human PBMCs	(112, 122, 125, 126)	NET and LNG did not exert any effect.
	ETG	>1000 nM		(126)	
↓ Levels of CD40 and CD80 on DCs	MPA	>0.31 nM	Human primary DCs	(127)	Consistent with animal and clinical studies ➀.
↓ Induction of T cell proliferation by DCs	LNG	>62 nM		(144)	
↓ Production of IFN-*α* and TNF-*α* by pDCs	MPA	>10 nM	Human PBMCs	(126)	Consistent with a clinical study (145) ➀. NET and LNG did not exert any effect.
	ETG	>1000 nM		(126)	
↑ Surface levels of CCR5 and CXCR4 on T cells	MPA	1000 nM	Human PBMCs	(122)	Direct effect of MPA may contribute to increased frequency of CCR5^+^ T cells in DMPA-using women ➁.
↑ Uptake and transcytosis of HIV-1 by epithelial cells	MPA	1 nM	Human primary genital epithelial cells	(130)	Consistent with a clinical and an animal study ➂.
↑ IL-12 and ↓ IL-10 production by epithelial cells	MPA	>1.5 nM	Human ectocervical epithelial cell line	(109)	Consistent with increased inflammation observed in some clinical studies ➃.
↑ IL-8 and RANTES		1 nM	Primary epithelial cells	(146)	Consistent with some clinical studies ➄.
↓ IL-6	MPA	1–25 nM	Human endocervical epithelial cell line and primary cells	(115, 130)	Consistent with decreased inflammation observed in some clinical studies ➅. No effect with NET (115).
↓ IL-8					

The numbered circles in the last columns of Tables 3, 4 and 5 indicate consistency in mechanisms between *ex vivo*, clinical and animal studies. Each number in a circle indicates a particular effect consistent across the Tables.

**Table 4.** **Summary of Data from Clinical Studies**

**Reported Effect**	**Progestin/Form of HC**	**Reference**	**Note**
↑ Surface levels of CCR5 on peripheral T cells	DMPA-IM	(147)	May reflect increased T cell activation or a direct effect of MPA on the levels of surface CCR5 ➁.
	LNG-IUD	(147)	
↑ Frequency of CCR5^+^ T cells in FGT mucosa	DMPA-IM	(148)	May reflect infiltration of activated T cells or a direct effect of MPA on the levels of surface CCR5, as shown for injectables (150) ➁. LNG-IUD users exhibited decreased frequency of CCR5 T cells in the endometrium and cervix (151). One study showed increased macrophages and decreased regulatory T cells in endometrium (152).
	COC	(149)	
	DMPA-IM or NET-EN	(150)	
↓ Production of IFN-*α* and TNF-*α* by circulating pDCs	DMPA-IM	(153)	Consistent with *ex vivo* and animal studies (126, 154) ➀.
	ETG (NuvaRing)		
↓ Thickness of vaginal epithelium	DMPA-IM	(155)	Significant epithelial thinning was not observed in most studies in humans (148, 156–158).
↑ Genital mucosal permeability	DMPA-IM	(133)	Consistent with an animal model study ➂.
↓ Genital expression of DSG1*α*			Permeability effect consistent with *ex vivo* study (130). Consistent with a clinical study ➂.
↓ Cervicovaginal levels of HBD2, SLPI, or other antiviral factors	DMPA-IM	(159–161)	Some reports show no effect for SLPI (159, 160, 162–164) or HBD2 (163) for DMPA-IM.
	COC or LNG-IUD	(165)	
↑ Cervicovaginal levels of select proinflammatory cytokines and chemokines	DMPA-IM	(159, 160, 162, 166)	Consistent with increased expression of IL-12 by epithelial cells ➃. Increased IL-8 and RANTES consistent with *ex vivo* study for MPA (130) ➄. Some studies show broadly proinflammatory effects (162, 163) for DMPA-IM.
	NET-EN	(162)	
	COC	(160)	
	LNG-IUD	(166)	
↓ Cervicovaginal levels of select cytokines and chemokines	DMPA-IM or NET-EN	(167)	Decreased IL-6 consistent with *ex vivo* studies for MPA (115, 130) ➅. Decreased RANTES consistent with *ex vivo* study for MPA (115) ➅. Some studies show a broadly immunosuppressive effect for injectables (167).
	DMPA-IM	(153)	

The numbered circles in the last columns of Tables 3, 4 and 5 indicate consistency in mechanisms between *ex vivo*, clinical and animal studies. Each number in a circle indicates a particular effect consistent across the Tables.

**Table 5.** **Summary of Data From Animal Studies**

**Reported Effect**	**Progestin**	**Organism/Model**	**Reference**	**Note**
↑ Risk of SIV and SHIV infection following vaginal exposure	DMPA	Rhesus macaque	(37, 168, 169)	
↑ SIV and SHIV plasma levels in the acute phase of infection	DMPA	Rhesus macaque	(37, 168)	
↑ Viral genetic complexity and frequency of CXCR4-using variants	DMPA	Rhesus macaque	(37)	
↓ Protective effect of prior immunization against SIV	DMPA	Rhesus macaque	(168, 169)	
↓ Thickness of vaginal epithelium	DMPA	Rhesus macaque	(170)	Epithelial thinning is less profound or undetectable in humans.
	DMPA	Pigtail macaque	(135, 171)	
	DMPA	Mouse	(172)	
	LNG	Pigtail macaque	(173)	
↓ Control of *Mycobacterium tuberculosis* infection	DMPA	Mouse	(131)	Consistent with the direct effect of MPA on immune cells ➀.
↓ Antigen-specific cytokine production				
↑ Susceptibility to HSV-1 and HSV-2	DMPA	Mouse	(36, 132, 133)	Consistent with the direct effect of MPA on immune cells ➀.
↓ Virus-specific immune response				
↑ Genital mucosal permeability and invasion of endogenous vaginal microbiota	DMPA	Mouse	(133)	Consistent with a clinical study ➂.
↓ Genital expression of DSG1*α*	LNG			
↑ Susceptibility to *C. trachomatis*	LNG	Mouse	(144)	Consistent with the direct effect of MPA on immune cells ➀.
↓ Activation and function of DCs and T cells				
↑ *α*_4_*β*_7_ on CD4^+^ cells in endocervix	DMPA	Rhesus macaque	(174)	

The numbered circles in the last columns of Tables 3, 4 and 5 indicate consistency in mechanisms between *ex vivo*, clinical and animal studies. Each number in a circle indicates a particular effect consistent across the Tables.

## The FGT: Susceptibility to HIV-1 and Other STIs

### Effects of endogenous ovarian hormones

The structure of the FGT and effects of estrogen and progesterone on FGT physiology have previously been comprehensively reviewed (175–179). We provide a brief overview with a focus on the issues particularly relevant to hormones, contraceptives, and susceptibility to infections.

The uterine endometrium in women of reproductive age is composed of the stratum functionalis (functional layer), the upper two-thirds of which undergo the cyclical changes of proliferation and degeneration during the menstrual cycle. The stratum basalis (basal layer) is not shed during menstruation and serves as the source of stem cells required for tissue rebuilding (175). The endometrial tissue has three main cellular components: the luminal epithelium and the epithelial glands that invaginate from the uterine lumen consisting of a single monolayer of columnar epithelium, the stromal tissue made up of fibroblasts, and, finally, the blood vessels that are lined by the endothelial tissues (180). All three structural components are exquisitely regulated by the two main female reproductive steroid hormones: estrogen and progesterone. Prior to puberty, the endometrial tissue is quiescent, lined by a thin epithelium and glands, a dense stroma, and thin blood vessels. Following menarche (initiation of the menstrual cycle), the cyclical pattern of hormones establishes a monthly cycle of endometrial tissue proliferation and differentiation, followed by tissue destruction and rebuilding, that is only interrupted by pregnancy. After menopause, the endometrium undergoes a gradual atrophy, ultimately resulting in a thin endometrium with flat epithelium and cystic glands (175).

The endometrium undergoes dramatic changes on a cyclical basis during a woman’s reproductive years, primarily in response to estrogen and progesterone (181). In a normal menstrual cycle, the endometrium undergoes proliferation in response to increasing levels of estrogen during the proliferative phase of the cycle. During this time, the endometrial thickness increases by >10-fold due to extensive proliferation of glands, stroma, and blood vessels. The active proliferation of the endometrial epithelium results in the glands growing and becoming tortuous (182). After ovulation, the secretion of progesterone leads to arrest of glandular proliferation and change to complex secretory activity. The most notable change during the secretory phase is in the blood vessels, with the arterioles undergoing proliferation and extensive coiling to form the spiral arteries in preparation for potential embryo implantation (183, 184). In the absence of implantation, the corpus luteum wanes and there is withdrawal of progesterone and estrogens, resulting in stromal breakdown, disruption of blood vessels, and shedding of the stratum functionalis.

The endometrial body in women, also known as the fundus, narrows at the inferior end forming the cervical canal, which projects a short distance into the vagina. The part of the cervix superior to the vagina is called the endocervix whereas the inferior part, facing the vagina, is called the ectocervix (178, 179). The endometrium is connected to the endocervix via the constricted internal orifice called the internal os and the ectocervix is connected to vagina via the external os. The cervix has a unique morphology in that the two parts of the cervix, the endocervix and the ectocervix, are lined by different types of mucosal epithelia. Whereas the endocervix is lined by a monolayer of columnar epithelium, a well-differentiated squamous epithelium lines the ectocervix. The columnar epithelium is tall, mucin secreting, and invaginates into the underlying stroma to form “glandular-like” structures. The squamous epithelium is stratified into three layers: (1) the basal cells, (2) the intermediate cells, and (3) the superficial cells. It undergoes continuous renewal through the processes of proliferation, differentiation, and maturation (178). Differences have been noted in the response of the three layers of cells to female sex hormones. Estrogen primarily induces proliferation and maturation of all three layers and desquamation, whereas progesterone causes thickening of the intermediate layer. ERs are expressed at similar levels and in all three layers of cells throughout the menstrual cycle (185, 186). PRs are expressed in intermediate cells during the secretory phase and pregnancy (186) and in endocervical columnar epithelial cells during the proliferative phase (187). When the progesterone-to-estrogen ratio is high, such as during pregnancy or the secretory phase of the menstrual cycle, the intermediate layer predominates. When the estrogen-to-progesterone ratio is higher, such as during the proliferative phase, superficial cells are predominant. In the absence of hormones, the squamous epithelium stays undifferentiated (188).

The area of the cervix where the columnar epithelium transitions to stratified squamous is known as the “squamo-columnar transition zone” (179). In adult reproductive-aged women, this squamo-columnar junction is usually inside the cervical canal. Under certain physiological or pathological conditions, the squamo-columnar zone moves toward the external os, exposing the endocervical columnar epithelium to the ectocervical and vaginal environment. This phenomenon is called cervical ectopy (189). The position of the squamo-columnar junction is important because abnormal precancerous squamous tissue is most prevalent in this area. Moreover, cervical ectopy has been correlated with increased susceptibility to some STIs in a number of studies (190–193).

The vagina is a muscular tube that extends from the cervix to the external genitalia. The vaginal wall is composed of the mucosal lining composed of the stratified epithelium, similar to the ectocervix, the subepithelial stroma, and the smooth muscle layer. The vaginal epithelium is very responsive to estrogen levels (194). Studies that have measured the thickness of the vaginal epithelium show a maximum of 28.1 ± 0.6 layers present in women during the follicular phase of the menstrual cycle and decrease to 26 ± 0.7 layers during the luteal phase (195). Nonovulating women had a mean of 23 ± 1.4 layers compared with 28.8 ± 0.7 layers in ovulating women (195). The vaginal epithelium is covered with a mucus layer and plays an important barrier function. A lower pH is important for protection against pathogens. Sex hormone receptors are expressed throughout the vaginal tissue and are particularly high in the epithelium (194). ER levels are downregulated after menopause (196). PR levels are highest when estrogen levels are high (186). Stromal cells also express ERs that are important for vaginal cell proliferation, whereas ER expression on both stroma and epithelium is important for differentiation and maturation of the stratified squamous epithelium (194). Some researchers have suggested that the superficial layers of the squamous epithelium are the equivalent of “stratum corneum” found in the epidermis consisting of terminally differentiated, keratinized enucleated, dead and flattened cells (197).

Following menopause, atrophy occurs in vaginal mucosa, primarily as a result of the loss of estrogen. The atrophic vaginal wall is thinner with few intermediate and superficial cells. The lower estrogen levels also lead to lower glycogen levels, changes in microflora, and increased pH (198).

### Modulation of immune environment by ovarian hormones

Tightly regulated production of estrogen and progesterone in the menstrual cycle exerts a dramatic effect on immune processes in the female reproductive tract and other mucosal tissues, as reviewed elsewhere (5, 199–202). The luteal phase of the ovarian cycle is associated with decreased production of antibodies, reduced cell-mediated cytotoxic activity, and decreased antigen presentation capacity of professional antigen-presenting cells (5). The state of partial immune suppression is observed in tissues of the upper (ovaries, fallopian tubes, and uterus) and lower FGT, composed of the endocervix, ectocervix, and vagina. Interestingly, pregnant or postpartum women with high progesterone levels have been shown to be more susceptible to HIV-1 (203–205), although whether this association is confounded by behavioral factors is unclear. It is thought that the evolutionary purpose of progesterone-mediated immune regulation is the reduction of the potentially harmful immune response to an implanting embryo. Importantly, however, note that progesterone can simultaneously exert stimulatory and suppressive effects, depending on the target cell population, activation state, and immune milieu, and that an inflammatory process is typically accompanied by an anti-inflammatory regulatory reaction. In general, progesterone shifts the focus of the immune system from a Th1 response (CD4^+^ Th1 cells primarily support CD8^+^ T cell–mediated cytotoxic mechanisms) to a Th2 response (CD4^+^ Th2 cells primarily support the production of antibodies by B cells). Estrogen exerts either a stimulatory or suppressive effect depending on cell type, environment, and hormone concentration (5).

### Mechanisms of HIV-1 acquisition in the FGT

To enter the target cell, HIV-1 utilizes the CD4 receptor and one of two chemokine coreceptors, either C-C chemokine receptor type 5 (CCR5) or C-X-C chemokine receptor type 4 (CXCR4). HIV-1 virions that use the CCR5 coreceptor are referred to as R5-tropic and virions using the CXCR4 coreceptor as X4-tropic (206). R5-tropic viruses are more common in the initial phase of infection; X4-tropic viruses are typically associated with an advanced disease state characterized by rapid T cell depletion, immune system collapse, and opportunistic infections that mark the onset of AIDS (207). Infection with HIV-1 occurs by the transfer of blood, semen, vaginal fluid, pre-ejaculate, or breast milk. In blood and body fluids, HIV is present in the form of free virus particles and infected immune cells. Both X4 and R5 HIV variants are present in body fluids that are passed during sexual intercourse. Interestingly, a selection process leads to a predominant transmission of the R5-tropic virus (208); however, the underlying mechanisms of the selection process are unclear. A recent study suggested that cervicovaginal inflammation may affect which HIV-1 variant infects the FGT (209). Typically, only a single or a very limited number of virions are transmitted from the donor, giving rise to the transmitted/founder population that rapidly disseminates throughout the body (210). The primary cell types that support HIV-1 proliferation in HIV-1–infected individuals are CD4^+^ T cells (Th cells) and macrophages. However, HIV can enter other nontarget cells such as CD8^+^ T cells, B cells, and epithelial cells using noncanonical receptors, although whether any viral replication can occur in these cells is unclear. As with all lentiviruses, HIV-1 replicates most effectively in cells that are actively proliferating. HIV-1 preferentially replicates in Th cells (CD3^+^CD4^+^) that express the markers of the memory T cell subset (CCR5^+^) and proliferation (*e.g.*, Ki67^+^) (211, 212). However, depletion of CD4^+^ T cells in SIV-infected macaques leads to increased viremia due to a massive infection of macrophages, microglia, and possibly other cell types, indicating that CD4^+^ T cells are not necessarily required for SIV proliferation (213).

The FGT provides multiple layers of protection against incoming pathogens, including circulating, tissue-resident, and transmigrating immune cells, the mucosal epithelial barrier, and secreted molecules with antibacterial and antiviral functions (5, 214). Additional protective responses are activated following the migration of pathogen across the mucosal barrier, including rapid innate immune responses, induction of adaptive responses and immunological memory, and accelerated reactivation of memory T and B cells following reinfection. The low probability of HIV-1 acquisition via heterosexual intercourse (estimated at 1:200 to 1:2000) is a testimony to the effectiveness of genital barrier function (214).

Evaluation of the effect of different forms of HC on HIV-1 acquisition is hindered by the fact that, despite decades of intensive research, our understanding of mechanisms of mucosal transmission of HIV-1 is rather limited (214, 215). It is unclear whether most heterosexual transmission events occur via free virus, cell-associated virus (donor cells that are either productively infected or carry membrane-associated virus or recipient cells that are present in FGT lumen and re-enter the mucosa following infection), specific capture by dendritic processes of LCs protruding from the epithelial layer to the vaginal lumen, transmigration of virus between epithelial junctions of columnar epithelium of endocervix and upper FGT, transcytosis through columnar epithelial cells, transport of sperm-associated virus to the upper FGT, or transmission via the microabrasions and tears in vaginal epithelium. All of these mechanisms have been described and are likely to occur *in vivo* (5, 214–219). Furthermore, it is unclear whether HIV-1 is preferentially transmitted via the multilayer stratified squamous epithelium of the ectocervix and vagina or single-layer columnar epithelium of the endocervix and endometrium. It has been argued that most heterosexual transmissions occur through the pluristratified epithelial layer of the vaginal and ectocervical mucosa. The combined surfaces of the vaginal wall and ectocervix exceeds by 15-fold the surface area of the endocervix. In support of this argument, HIV-1 infection occurs in female macaques after surgical removal of the uterus (220–222) and in women who lack a uterus at birth (220, 221). However, a recent study in macaques exposed to an SIV-based dual reporter system demonstrated that the virus rapidly disseminates across the entire lower and upper FGT, including the vagina, ectocervix and endocervix, ovaries, and local draining lymph nodes, within 48 hours of exposure (223). The mechanisms of HIV-1 transmission have been reviewed previously and are not discussed in detail in this review (5, 214–218).

## Progestins and Hypoestrogenism

Different forms of HC may have different effects on HIV-1 acquisition due to indirect effects on endogenous levels of estradiol. A comparison between mean circulating estradiol levels for different forms of HC reveals that DMPA-IM users are typically hypoestrogenic (155, 156, 224), with estradiol levels similar to those seen in postmenopausal women and lower than for any other form of progestin-only HC, including NET-EN (Fig. 3) (225). Women on HC have lower estrogen levels than do those occurring in the luteal phase, with a rank order of estrogen levels of Mirena > Implanon = Jadelle > NET-EN > DMPA (225). Hypoestrogenism may increase HIV-1 acquisition via effects on vaginal tract integrity, FGT microbiota (*e.g.*, a lactobacilli-dominated vaginal microbiome), and immune function in the FGT (225, 226). The effects of estrogen are complex and concentration-dependent (5, 227). Estrogen is generally shown to have protective effects for viral infection in mammalian FGTs (133, 170 225, 228–234). Collectively, these studies suggest that estrogen may provide a substantial protective effect, both in preventing the acquisition of HIV-1 and other STDs in postmenopausal women, as well as by vaginal administration to women on DMPA (225).

Smith *et al.* (229) have demonstrated that estrogen applied systemically in the form of subcutaneous implants protects against the intravaginal challenge of ovariectomized female rhesus macaques with highly pathogenic SIV_MAC251_. The block appears to be at the mucosal tissue level because rechallenging the animals by submucosal injection resulted in a productive infection. In a subsequent study, the authors showed that topical treatment with an intravaginal cream containing estrogen protects animals against intravaginal challenge (232). Topical application of estrogen resulted in a minimal increase in serum estriol or luteinizing hormone levels. In one study, 6 of 6 control animals but none of 6 estrogen-treated animals became infected; in the second study, only 1 of 12 of estrogen-treated animals, compared with 6 of 8 untreated controls, became infected. These studies suggest that vaginally applied estrogen may represent an inexpensive and safe method of HIV-1 prevention. However, note that most studies addressing the effect of estrogen were performed in ovariectomized macaques, not in intact cycling animals; this may have an important implication on the physiology of FGT (229, 232).

Potential mechanisms of estrogen-induced resistance of genital mucosal tissue to HIV-1 infection include increased thickness of the vaginal epithelium resulting in a barrier blocking the access of HIV-1 to parabasal and subepithelial target cells (5, 229, 232, 235), change in vaginal pH (229, 236–238), change in the production of chemokines and secretion of antiviral factors, and alteration of the type, frequency, and distribution of epithelial immune cells (5, 145, 239). An inverse correlation between epithelial layer thickness and susceptibility to SIV in estrogen- and progesterone-treated macaques was observed (228, 229, 232). One study suggested that estrogen decreases the frequency of LCs in the vaginal epithelial and stromal tissue (145); however, this observation has not been supported by other studies and needs to be re-evaluated (153). Estrogen treatment decreases cervicovaginal pH, making the environment hostile to the virus, and stimulates the growth of protective H_2_O_2_-producing lactobacilli (5, 155, 229, 240–243). However, because all of these studies were performed either in SIV models or *ex vivo*, it remains to be determined whether estrogen has similar effects in human vaginal epithelium *in vivo*.

“DMPA has been shown in a number of rodent studies to decrease the epithelial thickness significantly, consequently affecting the susceptibility to viruses and bacteria.”

## Animal Studies Addressing the Effect of HC on the Acquisition of Genital Pathogens

Studies in nonhuman primates indicate that treatment with DMPA enhances the risk of SIV acquisition following vaginal exposure to the virus and significant increases viral levels in the acute phase of infection (37, 168, 169). DMPA was shown to enhance the genetic complexity of the replicating virus, increase the replication of the CXCR4-dependent subtypes, and slow the induction of cellular immune response (37). DMPA also reduces the protective effect of prior immunization with attenuated virus (168, 169). DMPA is routinely used in nonhuman primate (primarily rhesus and pigtail macaque) studies to ensure effective SIV acquisition following vaginal exposure. The combination of a high challenge dose with DMPA treatment typically results in 100% infection rate whereas in the absence of DMPA the infectivity is lower. In contrast, DMPA does not affect the course of SIV infection following injection of SIV under the genital mucosal barrier or following intravenous challenge (229, 244).

To correctly interpret the studies in nonhuman primates and assess their relevance for HIV-1 acquisition in humans, it is important to consider the differences between humans and macaques with respect to the characteristic features of the menstrual cycle, physiology of the genital tract, and the relative effect of endogenous and exogenous hormones on the vaginal and ectocervical epithelium. Some primates undergo cycling associated with seasonal mating; this can be altered in captivity. As discussed later, rhesus and pigtail macaques respond to treatment with DMPA or LNG by significant thinning of vaginal epithelium (135, 170, 171, 173). However, the change in epithelial thickness and number of epithelial levels is less profound or undetectable in humans (148, 155–158). Macaques intravaginally exposed to SIV are less susceptible to infection when in the follicular compared with the luteal ovarian phase (245, 246). This susceptibility pattern reflects the cycling of epithelial thickness and degree of keratinization that are highest at the peak of the follicular phase around ovulation and lowest during menses (247). Because female macaques are more likely to have intercourse during ovulation, increased thickness and keratinization of the epithelium represents a physiological mechanism preventing the traumatic effect of intercourse and infection with pathogens introduced during the intercourse.

In most studies, the dose of MPA per kilogram of body weight used in macaques is higher than the dose delivered by DMPA-IM in humans. In earlier papers, 30 mg (∼4 to 5 mg/kg body weight) of MPA was used in macaques compared with 150 mg (∼2 mg/kg) in DMPA-IM in humans; therefore, an ∼2- to 2.5-fold higher dose per kilogram of body weight was used in macaques. The reported *C*_max_ of MPA in rhesus macaques 14 days following injection of 30 mg of DMPA is ∼5.5 ng/mL or 15 nM (170). This is similar to the reported average *C*_max_ values in humans using DMPA-IM (see “Contraceptives: Actions, Types, and Serum Concentrations” previously). In rhesus macaques, MPA concentrations decrease to ∼1 ng/mL (2.6 nM) by day 50, a concentration that is comparable to humans on DMPA-IM (Table 1). Further complicating the interpretation of experimental data obtained in animal models is the possibility that substantial differences may exist between humans and nonhuman primates in the pharmacokinetics and pharmacodynamics of MPA and other hormones (134, 171, 248). Further studies are required to establish this, given the difficulty in comparing nonequivalent doses between humans and monkeys in nonparallel studies (248), the large degree of variation in reported MPA serum concentrations between studies and individuals on DMPA-IM in humans, as well as the variation in apparent half-life values for the latter due to different sampling times for the establishment of *C*_max_ (46, 74). Using similar doses per kilogram of body weight in monkeys and humans may not result in comparable serum levels because metabolism may vary between species (248). Two studies have investigated the effects of equivalent doses in monkeys compared with women. Radzio *et al.* (134) have defined a 3-mg DMPA monthly dose (0.3 to 0.4 mg/kg) in pigtail macaques that suppresses ovulation and mimics other biological effects seen in women on DMPA-IM, and results in similar *C*_max_ serum concentrations of ∼3 ng/mL (8 nM). In this model, the authors found a correlation between plasma MPA concentration, epithelial thickness, and CD3^+^ T cell density in vaginal tissue but little or no effect on plasma viremia following vaginal SHIV infection. In another study in pigtail macaques, Butler *et al.* (135) applied 1.5 mg DMPA/kg monthly, resulting in peak MPA serum levels from ∼8 ng/mL (20 nM) after one injection to ∼15 ng/mL (39 nM) after four injections, both within the reported wide range of DMPA-IM *C*_max_ values in women. DMPA-treated macaques became infected after a median of 5.5 intravaginal challenges with SHIV, compared with 9 exposures needed in controls.

Several studies in small animal models have also attempted to mimic MPA levels comparable to those observed in women using DMPA-IM. In one study, mice were injected weekly with 1 mg of MPA, resulting in serum concentrations at the upper limit of detected *C*_max_ levels of MPA in women (∼100 nM) (131). At this concentration, MPA impaired the control of *Mycobacterium tuberculosis* and inhibited antigen-specific cytokine production (131), raising the important question as to whether HC affects control of *M. tuberculosis* in women. Importantly, MPA radically enhances the susceptibility of mice to HSV-1 and HSV-2 and is commonly used to facilitate the infection in this model of sexually transmitted disease (36). Treatment of mice with 2 mg of DMPA changes the susceptibility to genital HSV-2 infection and inhibits local innate and adaptive immune responses (36). Injection of mice with 1 to 4 mg DMPA prior to HSV-1 infection results in impaired function of DCs, T cells, and reduction of virus-specific immunological memory (132). A recent elegant study in mice injected with 1 mg of DMPA demonstrated reduced genital expression of the desmosomal cadherin desmoglein-1*α* (DSG1*α*), increased genital mucosal permeability, and increased susceptibility to HSV-1 viral infection (133). A DMPA-mediated increase in mucosal permeability promoted tissue inflammation most likely by facilitating the invasion of endogenous vaginal microbiota. A similar effect was observed with LNG (133). In a murine model of intranasal *Chlamydia trachomatis* infection, LNG suppresses DC activation and function, inhibits the formation of T cell immunity, and increases susceptibility to the pathogen (144).

In both nonhuman primate and rodent models, DMPA has a profound effect on vaginal histology. DMPA and Norplant implant (LNG) lead to significant thinning of vaginal epithelium in rhesus macaques within 30 to 60 days of the start of treatment, but the effect is rapidly reversed following treatment withdrawal (170). Studies done in pigtailed macaques show a similar thinning of vaginal epithelium following DMPA and COC (LNG/EE) treatment (173). However, studies in untreated nonhuman primates show that there may be changes to vaginal epithelium thickness even during the normal menstrual cycle, with 30% thinning of epithelium in pigtail macaques during the secretory phase in pigtailed macaques (249). In rodents, the phenomenon of vaginal epithelium thickness being regulated by sex hormones is well documented, with estrogen leading to significant thickening and progesterone causing thinning of vaginal epithelium (202, 233). DMPA has been shown in a number of rodent studies to decrease the epithelial thickness significantly, consequently affecting the susceptibility to viruses and bacteria (202, 172, 250).

## Effects of HC on the Acquisition of Other Sexually Transmitted Pathogens

The World Health Organization estimates that every year there are 357 million new infections with primarily one of the four bacterial STIs, that is, chlamydia, gonorrhea, syphilis, and trichomoniasis (251, 252). In addition to these, 24 million new HSV-2 infections occur annually (253). Although most of the STI burden is borne by developing countries, bacterial STIs represent a major problem in developed countries as well. Women typically have more asymptomatic infections compared with men and suffer disproportionately more severe consequences from bacterial STIs, including pelvic inflammatory disease, infertility, and ectopic pregnancy. Furthermore, STIs have been shown to increase the risk of HIV acquisition (254, 255). Whether the use of HC increases the risk of STI acquisition has very important public health implications given that almost 200 million women use various forms of HC worldwide (256, 257); however, a comprehensive examination of this topic is beyond the scope of the current review. In this section we focus primarily on evidence regarding the effect of hormones and HCs on STIs that have been shown to increase the risk of HIV acquisition, because increase in these coinfections can indirectly enhance HIV risk.

A number of clinical studies have examined the relationship between the use of HC and acquisition of STIs, with no clear etiological association. Two systematic reviews (258, 259) examined published studies on this topic and concluded that overall there is a positive correlation between oral contraceptive and chlamydial infection, but not with gonorrhea, HSV-2, trichomoniasis, syphilis, and human papillomavirus (HPV). Both reviews cited limitations in the amount and quality of evidence from the studies. Among the major drawbacks cited were small sample size, inadequate adjustment for confounding factors, and questionable relevance of studies conducted in high-risk subgroups to the general population. Most of these studies on this topic examine the association with COCs, DMPA-IM, and IUD, with few studies on NET-EN and other forms of progestin-only methods and none on newer combined hormonal methods, including patches, rings, and injectables.

Whereas the prospective and cross-sectional studies considered in systematic reviews did not find strong evidence linking HCs with STI acquisition, other studies have shown a link between HC use and conditions such as cervical ectopy, which is associated with acquisition of chlamydia, HPV, HIV, and CMV [reviewed in Venkatesh and Cu-Uvin (260)]. Similarly, although HCs have not been shown to directly increase HPV infection, a known cause of cervical cancer, recent results from the EPIC Cohort suggest hormonal factors as risk factors for cervical carcinogenesis (261). In this cohort, the number of full-term pregnancies and long-term use of oral contraceptives was associated with increased risk of developing cervical intraepithelial neoplasia grade 3 and invasive cervical cancer. Another recent prospective study conducted in Rakai, Uganda, in HIV-1– and HSV-2–negative women showed an adjusted hazard ratio of 2.26 for HSV-2 acquisition in women using DMPA-IM compared with women not on HC (262). However, the study power was low and the authors suggested a larger study with more frequent follow-ups.

“More studies are needed to confirm which of these mechanisms are key for an HC-related increase in susceptibility to STIs in women.”

The underlying biological mechanism of how HCs could facilitate the acquisition of STIs is still incompletely understood and is an active area of investigation. A number of reviews have examined the underlying mechanisms of hormonal influence on STIs (263) and individual infections, including chlamydia (86). Many of these studies have examined the effects of the reproductive cycle and exogenous hormones, including DMPA-IM on STIs, and including HSV-2, chlamydia, and gonorrhea in animal models, primarily rodents. No studies in nonhuman primate models have examined the effect of hormones on STIs, other than SIV (264, 265). Rodent data indicate that overall estradiol is protective whereas progesterone enhances primary infection to HSV-2 (233). In mice, DMPA was also shown to increase susceptibility to HSV-2 and decrease immune responses (36, 172), whereas estradiol treatment was recently shown to enhance protection by augmenting antiviral Th1 responses (226). Chlamydia infection may be enhanced by both progesterone and estradiol, depending on the animal model used and location of primary infection (lower or upper genital tract) (86), whereas gonorrhea infection is exacerbated by estradiol in experimental models (266). A recent systematic review reported that bacterial vaginosis (BV) was reduced and vaginal candidiasis was not increased in DMPA-IM (and most likely NET-EN) users, but COCs (progestin component not recorded) may predispose for vaginal candidiasis (267).

Combined information from animal studies and *ex vivo* and *in vitro* studies suggests a number of possible mechanisms of hormone/HC effect, including (1) increased local inflammation and chemokine production resulting in recruitment of target cells to the genital tract, (2) increased mucosal permeability promoting inflammation, (3) increased cervical ectopy, (4) thinning of vaginal epithelium and reduced secretion of protective factors, and (5) a hypoestrogenic effect resulting in decreased colonization with lactobacilli, resulting in changes in pH and increased colonization by BV-associated bacteria. More studies are needed to confirm which of these mechanisms are key for an HC-related increase in susceptibility to STIs in women.

## Contraceptives, Immune Function, and HIV-1 Acquisition in the FGT

Multiple lines of evidence from clinical, animal, and *in vitro* studies indicate that HCs affect the function of the immune system, both systemically and locally, by altering the ability of the FGT to protect against invading pathogens (Tables 3–5). Several studies have reported that MPA is immunosuppressive *in vivo* and *ex vivo*, as outlined later.

Immunosuppression is broadly defined as a reduction in the capacity of the immune system to react to a challenge, for example, by an invading pathogen. Deliberately induced immunosuppression is used to prevent a rejection of an organ transplant or to ameliorate the effect of an autoimmune disease. Nondeliberate immunosuppression is associated with aging, malnutrition, cancer, and several chronic infections, including HIV-1/AIDS. Immunosuppression affects both innate and adaptive immune systems. The innate immune system is an evolutionarily older type of defense that protects the host in a nonspecific manner. In contrast, the adaptive immune system, mediated primarily by T cells and antibody-producing B cells, is evolutionarily younger and provides antigen-specific immunological memory after the initial response to a pathogen, leading to an enhanced response to subsequent encounters. The professional antigen-presenting cells, such as DCs and macrophages, play a critical role in connecting the innate immune system with the adaptive responses. As outlined later, MPA was reported to regulate several functions of both the innate and adaptive immune systems.

### Effects on the function of DCs and pDCs

DCs are professional antigen-presenting cells that are indispensable for the initiation of adaptive immune response. DCs uptake and degrade the antigen and present short peptides to CD4^+^ and CD8^+^ T cells to induce antigen-specific immune responses. Studies in animal models demonstrated that DMPA-IM suppresses DC function, reduces expansion of virus-specific T cells, and impairs the development of virus-specific immunologic memory (132). Vicetti Miguel *et al.* (132) reported that DMPA administered to female mice prior to mucosal tissue infection with HSV-1 suppressed DC activation and surface levels of costimulatory molecules CD40 and CD80. This inhibition of DC activation subsequently impaired expansion of virus-specific T cells and the development of HSV-1–specific immunologic memory. In a subsequent study, the group demonstrated that MPA significant reduced CD40 and CD80 expression on human primary DCs and reduced their ability to induce T cell proliferation at a concentration as low as 0.31 nM (127).

In addition to its effect on conventional DCs, MPA also suppresses the function of pDCs. pDCs are a key immune population that function as sentinel cells with the ability to recognize early viral and bacterial infections. pDCs orchestrate antiviral response by the release of soluble factors such as IFN-*α* (268–270). IFN-*α–*producing pDCs were shown to accumulate in the genital lamina propria early in SIV infection and are likely to contribute to the early immune response to the virus in monkeys (270). *In vitro*, MPA reduces the production of IFN-*α* in response to Toll-like receptor (TLR) 7 and 9 ligands at MPA concentrations of ∼10 nM corresponding to the *C*_max_ observed in the serum of DMPA-IM users (122, 126). *In vivo*, the use of DMPA-IM and NuvaRing (releasing ETG) is associated with a decreased function of circulating pDCs in HIV-1–uninfected women (153). The latter study shows that the function of pDCs is altered by exposure to systemic concentrations of MPA *in vivo* in DMPA-IM users. These data, consistent with a study in mice (154) and further supported by decreased levels of IFN-*α* in plasma and cervicovaginal lavage of DMPA-IM users (153), demonstrate the *in vivo* immunosuppressive effect of DMPA-IM on this critical innate immune cell population. Suppression of pDC function may tip the balance between the proliferation of a founder viral population and early immune control during the acute phase of infection in favor of the transmitted virus.

Less information is available on the potential effect of other progestins on the function of DCs and pDCs. Quispe Calla *et al.* (127) reported that LNG inhibits the activation of human DCs, lowers the expression of CD40, CD80, and CD86, and reduces their capacity to promote T cell proliferation at concentrations of ≥62 nM. Additionally, treatment of mice with LNG reduces DC function, reduces the recruitment of CD4^+^ and CD8^+^ T cells in lungs, and delays the clearance *C. trachomatis* following intranasal infection (144). The observed decreased function of pDCs in women using NuvaRing raises a concern that should be addressed in future studies (153). Head-to-head comparison of the effect of various progestins on the function of human pDCs *ex vivo* demonstrated that progesterone, LNG, and NET do not substantially inhibit pDCs *ex vivo* whereas ETG exerts a partial suppressive effect at 1 µM concentration (126). The lack of an effect with progesterone, LNG, and NET and lower potency of ETG compared with MPA is consistent with their lower relative affinities for the GR (Table 2), as well as the lower relative potencies of progesterone and NET compared with MPA for repression of gene expression via the GR (100, 103, 115).

Collectively, a substantial degree of coherence exists between the clinical, animal, and *ex vivo* studies indicating an immunosuppressive effect of MPA on the function of DCs and pDCs, populations that are critical to pathogen recognition, antigen presentation, and induction of T cell responses. The effect is systemic and occurs at concentrations equivalent to or lower than the pharmacological levels of MPA following the administration of DMPA-IM or DMPA-SC. Whether the potential effect of LNG and ETG on DCs has any clinical implications remains to be established.

### Effects on intraepithelial LCs

HC may modulate the susceptibility to SIV and HIV-1 by altering the function, frequency, and availability of target cells in the vaginal epithelium and stroma. Of particular interest is the population of LCs, a specialized type of DC that resides in the skin and is abundant in the epithelial layer of the vaginal and ectocervical mucosa of the female lower reproductive tract. Dendritic processes from LCs extend to the lumen of the vagina to sample antigens and bacteria. Several reports employing isolated human LCs and explant models of HIV-1 infection suggest that LCs acquire the virus via CD4, CCR5, and Langerin-dependent binding (214, 216, 271). LCs in the epidermis express Langerin, a specific C-type lectin receptor involved in HIV capture and destruction. Recently, the specific mechanism leading to this HIV restriction was discovered. Nevertheless, LCs can be infected and the way HIV escapes this restriction needs to be investigated (272, 273). Although only a few LCs may become productively infected, they are equipped to transport the membrane-associated virus to CD4^+^ T cells and macrophages in the lamina propria and draining lymph nodes (Trojan Horse theory) (214, 216, 271). It has been reported that exogenous estrogen decreases (145) whereas progesterone increases (239) the frequency of LCs in the human vaginal epithelial and stromal tissue. In contrast, other studies using S100 as a marker for vaginal LCs have not detected any difference between DMPA-IM users and controls (156–158). In a recent study involving a detailed analysis of immune cell frequencies and epithelial location of relevant cell populations in users of DMPA-IM, NuvaRing, and COC and in control volunteers, the frequency of intraepithelial LCs was significant decreased in both COC and NuvaRing users compared with either DMPA-IM users or controls (153). However, no significant difference in LC density or localization was detected between DMPA-IM users and controls, consistent with several previous studies (156–158, 274). Overall the studies are inconsistent. As it has been previously suggested that subsets of vaginal LCs differ in their expression of S100 and CD207 markers (271), differences in experimental approaches and population markers may account for the differences between studies. No study has effectively addressed the potential effect of HC on the functional properties of LCs such as their ability to bind HIV-1 virions and transfer them to draining lymph nodes. Thus, a substantial research gap exists in this area of investigation.

“The presence of a GR agonist results in a translocation of GR-VPR complex into the nucleus and efficient proviral integration.”

### Effects on the function, frequency, and infectivity of T cells

Multiple studies have reported that HC affects the function, frequency, and state of activation of T cells, both systemically and locally in the FGT. Additionally, HC was shown to exert a direct effect on the rate of HIV-1 proliferation inside the infected T cell and on the expression of surface receptors facilitating HIV-1 entry. T cells are broadly divided into CD8^+^ cytotoxic T cells with the ability to recognize and kill infected target cells and CD4^+^ Th cells that promote the function of CD8^+^ T cells, B cells, and other components of the immune system. CD4^+^ Th cells are further divided into Th1 cells that focus the immune response primarily to cytotoxic responses, Th2 cells that promote humoral responses, and Th17 cells that play a critical role in maintaining mucosal permeability. To communicate and direct the immune system, T cells produce a plethora of cytokines and chemokines. MPA was shown to suppress the production of several key T cell–derived regulators of cellular and humoral immunity involved in the induction of mouse and human immune response to invading pathogens including IFN-*γ*, IL-2, IL-4, IL-6, IL-8, IL-12, macrophage inflammatory protein (MIP)-1*α*, and TNF-*α* (112, 122, 125, 126, 131). DMPA suppresses *Mycobacterium bovis* bacillus Calmette-Guérin–induced cytokine production in human PBMCs and inhibits the function of cytotoxic T cells in DMPA-IM users (112, 131, 132, 154, 275). In these studies, short-term (24-hour) coincubation of T cells with MPA resulted in a significant inhibition of T cell function at MPA concentrations equal to or higher than the *C*_max_ in DMPA-IM users (≥10 nM or 3.8 ng/mL). However, prolonged exposure to progestins may enhance the effect. MPA impairs effector function of CD8^+^ T cells interacting with HSV-1 latently infected neurons, including their ability to produce IFN-*γ* and TNF-*α*, in 4- to 5-day *ex vivo* trigeminal ganglia cultures at concentrations as low as 0.1 nM (275). Additionally, long-term exposure to progestins *in vivo* may enhance their effect by altering the development of lymphoid cell lineages and homeostasis of memory cell populations. All of these factors may affect the functional capacity of T cells to respond to the antigenic challenge (5).

Ex vivo studies with freshly prepared, untouched T cells obtained from blood or genital mucosal tissue provide an opportunity for head-to-head comparison between different types of progestins that may be instrumental for informed decisions regarding prioritization of HC candidates for women at risk for HIV-1 and other infections. Although MPA is immunosuppressive for selected regulators at concentrations from 10 nM to 100 nM, LNG and NET do not inhibit the production of these cytokines by activated T cells at a concentration as high as 1 µM, whereas progesterone results in variable levels of repression but with a potency at least 100-fold less than MPA (122, 123, 126, 131, 153). These data thus suggest that LNG and NET do not inhibit immune cell function at systemic concentrations observed in HC users (122, 125, 126). ETG suppresses the function of T cells at concentrations ≥ 1 µM. This concentration is above the observed levels in plasma; however, local concentration in ETG of HC users may be substantially higher than the concentration in plasma. As previously discussed, NET and LNG have relatively low affinity for the GR and do not appear to mediate transrepression of the selected genes involved in immune regulation. ETG binds GR with intermediate affinity (120, 276). Because the GR regulates transcription of a variety of genes involved in immunity and inflammation (136–139, 142, 277, 278), the differential affinity for and activity of progestins via the GR (MPA >> ETG > LNG ≈ NET) is consistent with the hypothesis that progestins exert their suppressive effect on T cell function primarily via the GR (24, 25, 103, 122, 125, 128, 143). Interestingly, patients who are infected with HIV-1 display GC hypersensitivity associated with reduced T cell function, possibly due to the specific binding of the HIV-1 accessory protein Vpr to the GR coactivating motif (128, 143, 279, 280). Thus, the sensitivity of T cells to the effect of GR-binding progestins may be significantly enhanced in the context of HIV-1 infection (128).

Importantly, MPA directly increases the rate of viral replication of HIV-1 in human PBMCs, CD4^+^ T cells, and CD14^+^ monocytes (122, 129). The effect on viral proliferation is observed at concentrations as low as 0.1 nM (129). The observed increase in HIV-1 replication may be caused by several possible mechanisms, including increased retention of HIV-1 coreceptors on the surface of CD4^+^ T cells, inhibition of production of antiviral factors, and a direct effect on HIV-1 transcription. Because most proliferation probably takes place in CD4^+^ T cells, MPA likely affects CD4^+^ T cells directly, although the contribution of an indirect effect cannot be excluded. It is possible that the enhancement of HIV-1 proliferation may be mediated by direct binding of an activated GR-MPA complex to a GC response element half-site located within the HIV-1 LTR. However, although putative GR binding sites have been located on the HIV-1 LTR, GC agonists have been shown to both activate and repress the viral LTR (281–288) [reviewed in Hapgood and Tomasicchio (143)]. Importantly, activation of the GR releases unstimulated PBMCs from an early block in HIV-1 replication via a mechanism requiring Vpr (289). The presence of a GR agonist results in a translocation of GR-VPR complex into the nucleus and efficient proviral integration. This mechanism may be particularly important in the early stage of infection.

HC may also affect the expression of surface coreceptors CCR5 and CXCR4 that, in concert with CD4, facilitate the entry of HIV-1 into the target cell. The use of COCs was shown to be associated with increased activation and elevated expression of HIV-1 coreceptor CCR5 on intraepithelial endocervical CD4^+^ T lymphocytes; the specific progestin used in this study was not specified (149). Sciaranghella *et al.* (147) have demonstrated that LNG and DMPA-IM use was associated with increased peripheral blood T cell CCR5 expression. Chandra *et al.* (148) have demonstrated that 12 weeks following a 150-mg intramuscular injection of DMPA-IM in women volunteers, the numbers of CD45, CD3, CD8, HLA-DR, and CCR5-bearing immune cells in vaginal tissues were significantly increased compared with the follicular or luteal phases of untreated cycles. This study found no significant differences in immune cell populations between the follicular and luteal phases of the hormonal cycle. A trend toward increased CD3^+^ T cell density in DMPA-IM users compared with controls was detected in another recent study (153). Furthermore, increased levels of progesterone in pregnancy directly correlate with increased CCR5 expression on T cells in peripheral blood and genital tissue (290). However, others report that exogenous progesterone decreases CCR5 levels and increases CXCR4 levels in PBMCs from HIV-1–uninfected women (291). It is known that estrogen and progesterone affect the recruitment of inflammatory T cells and macrophages through downregulation of intracellular adhesion molecule-1, E-selectins, and vascular cell adhesion molecule-1 (292). The effect of progestin on T cell recruitment may, therefore, be either direct or indirect via the modulation of estrogen levels. A recent study of a cohort of women in South Africa demonstrated that the use of injectable progestin-only contraceptive was associated with a 3.9-fold increase in the frequency of cervical CD4^+^CCR5^+^-activated T cells and an increased expression of CCR5 on CD4^+^ cervical cells (150). A similar increase was found in the luteal phase in women not using HC, indicating that the ovarian cycle exerts a similar effect on the frequency of HIV-1 target cells (150). *Ex vivo* analysis demonstrated that 1 µM MPA can prevent the downregulation of CXCR4 and CCR5 on activated T cells, suggesting that this mechanism may contribute to the observations *in vivo*, although the effects of lower MPA concentrations were not reported (122). Because activated CCR5^+^ T cells are more prone to infection and support a higher rate of viral proliferation than do resting cells (212), increased frequency of these target cells in the cervix may accelerate viral dissemination after exposure to HIV-1 in the FGT. In contrast to the previous studies, Mitchell *et al.* (293) found a decrease in the frequency of CD3^+^ and CD3^+^CCR5^+^ vaginal T cells in long-term DMPA-IM users. A recent study by Smith-McCune *et al.* (152) identified increased frequency of activated T cells in the endometrium of DMPA-IM users; however, no increase in CCR5 expression on CD4^+^ T cells was observed. Interestingly, these same authors also identified an increased density of endometrial macrophages and a decreased density of endometrial regulatory T cells in DMPA-IM users. Furthermore, Goode *et al.* (174) have shown that rhesus macaques treated with DMPA for 5 weeks exhibited higher expression of integrin *α*_4_*β*_7_, the gut-homing receptor that is expressed on cells with increased susceptibility to HIV-1, on CD4^+^ T cells in the endocervix compared with animals treated with estrogen. This may be particularly important in light of the observation that administration of an anti-*α*_4_*β*_7_ monoclonal antibody prior to and during acute infection protects rhesus macaques from intravaginal transmission of SIV (294). Whether this occurs in humans and whether other forms of HC affect the frequency of activated CCR5^+^ T cells or the level of expression of *α*_4_*β*_7_ on T cells in the FGT in animal and human models are currently unknown.

A subset of T cells that may be particularly relevant for HIV-1 infection are Th17 cells. Th17 cells have been shown to be preferentially infected following SIV vaginal transmission (295) and are depleted in organized lymphoid tissue early in infection (296). As these cells accumulate at the site with weakened barrier function, they may represent the primary target CCR5^+^CD4^+^ T cell population (295). Xu *et al.* (297) have shown that progesterone regulates the expression of Th17-related transcription factor retinoic acid–related orphan receptor *γ*t (ROR*γ*t) in murine vaginal gonococcal infection. Estradiol enhances vaginal antiviral immunity by induction of Th17 responses. Whether HC affects the frequency and function of Th17 cells in genital tissues is currently unresolved (226).

In summary, although a substantial coherence exists between *ex vivo* experiments using purified human cell populations, animal studies, and clinical observations, the potential effect of HC on T cells is likely multifactorial and individual components are difficult to discern. For example, the immunosuppressive effect of MPA on the functional response of T cells most likely synergizes *in vivo* with its effect on the capacity of DCs to present antigen and costimulate T cell response by direct cell-to-cell interaction, including costimulation of T cells via CD40, CD80, and CD86. The direct effect of progestins on T cells appears to be mediated primarily via the GR and is less pronounced or undetectable with progestins exhibiting low GR affinity.

“Several clinical studies show a correlation between increased FGT inflammation and increased HIV-1 acquisition.”

### Effects on soluble antimicrobial factors

Accumulated evidence indicates that DMPA reduces the FGT levels of several antimicrobial factors with HIV-1 inhibitory activity. Human *β*-defensins (HBDs) 1, 2, and 3 as well as secretory leukocyte protease inhibitor (SLPI) are essential components of an epithelial cell–derived antimicrobial peptide complex that functions to keep the natural microbial flora in a steady-state (298, 299). HBDs inactivate HIV-1 before and following transepithelial migration (300–302). Mucosal expression of HBD2 and HBD3 is increased in exposed seronegative individuals (303), and HBDs were shown to promote epithelial cell proliferation and wound healing (304–306). A large prospective study of 4531 volunteers in Uganda and Zimbabwe demonstrated decreased cervicovaginal levels of HBD2 and higher RANTES in DMPA-IM users and an association between low levels of these factors and HIV-1 seroconversion (159). Lower cervical levels of HBD2 in DMPA-IM users were confirmed in a study involving 832 women (160). In an unrelated study, use of a COC (progestin not specified) or an LNG IUD was associated with decreased expression of HBD1 and HBD2 (165). Decreased SLPI expression was also observed in endometrial biopsies of new DMPA-IM users (161). In contrast, no effect of DMPA-IM (159, 160, 162–164) or NET-EN on SLPI levels in FGT was observed in a cross-sectional study in Kenya and South Africa (162). Francis *et al.* (163) have observed increased levels of HBD4 but, in contrast to other studies (159–161, 165), they observed a nonsignificant trend toward increased levels of HBD2 and HBD3 in cervicovaginal lavages of women using DMPA or COC.

Progesterone and progestins also exert a strong immunosuppressive effect on the production and transepithelial transport of immunoglobulin (Ig)G and IgA, as reviewed elsewhere (5, 163, 307). DMPA-IM treatment decreases specific IgG and IgA responses in mice following intravaginal or intranasal immunization with attenuated HSV-2 and causes failure to develop protective responses (36, 172, 310). Progestin-containing contraceptives have also been reported to decrease the levels of IgG and IgA in humans (309, 311, 312).

Collectively, based on the documented contribution of HBDs and SLPI to anti–HIV-1 activity in the FGT, it is feasible that reduced mucosal levels of these factors may facilitate HIV-1 transmission. Reduction of mucosal production of specific IgG and IgA may contribute to infection in pre-exposed individuals. A substantial research gap remains in our understanding of the effect of progestins other than MPA and whether the reduced production of mucosal antiviral factors is a direct effect of progestins or an indirect effect of hypoestrogenism associated, in particular, with DMPA-IM use. The inconsistencies in reported data regarding antimocrobial factors could be due to a number of confounding factors, similar to those resulting in inconsistencies in reported clinical effects on inflammation and levels of soluble immune modulators, as discussed in detail in the next section.

### Effects on inflammation and levels of soluble immune modulators

Several clinical studies show a correlation between increased FGT inflammation and increased HIV-1 acquisition (313, 314). Because several reports have suggested an increased level of FGT inflammation in women using DMPA-IM and other HCs, this may represent an indirect mechanism increasing the probability of HIV acquisition. However, the effect of HC on the levels of proinflammatory and anti-inflammatory cytokines and chemokines in the FGT is a subject of substantial controversy.

Selected soluble modulators have been shown to be regulated directly by MPA in genital tract cell models *ex vivo*, both in a proinflammatory and an anti-inflammatory manner, at concentrations spanning the peak and plateau levels in DMPA-IM users. Using an *in vitro* ectocervical cell culture model, MPA was shown to increase the expression of the proinflammatory cytokine IL-12, whereas the expression of IL-10, a cytokine that is generally regarded as anti-inflammatory, was suppressed in a dose-dependent manner via a mechanism involving GR (109). A study on primary human endometrial and endocervical epithelial cells exposed to HIV-1 *ex vivo* reported a broad upregulation of chemokines but not proinflammatory cytokines, with increased production of IL-10, monocyte chemoattractant protein 1 (MCP-1), IL-8, RANTES, and MIP-1*β*, and chemokine (C-X-C motif) ligand (CXCL)10 and decreased production of IL-6 and TNF-*α* in the presence of MPA (1 nM) (130). Most of these genes were not similarly regulated by 100 nM progesterone. MPA was also shown to increase IL-6 levels in a human vaginal cell line at ∼37 nM, whereas higher concentrations increased expression of other proinflammatory cytokines such as tumor necrosis factor (TNF), IL-8, and RANTES (124). In other studies, MPA at 4 to 25 nM, but not progesterone or progestins that have no GR activity such as NET or LNG, was shown to suppress the production of selected proinflammatory cytokines IL-6, IL-8, and RANTES and increase the expression of the anti-inflammatory GILZ gene in a human endocervical cell line (115). Ectocervical, endocervical, and vaginal cell lines exhibited different patterns of regulation of selected immunomodulatory genes in response to receptor-saturating doses of MPA, NET, and progesterone *ex vivo* (315). Collectively, these studies suggest that MPA exerts direct stimulatory and inhibitory effects on inflammation via the regulation of expression of selected genes in genital epithelia in a cell-specific manner.

Some clinical studies have reported no association between injectable contraceptive usage (76% on DMPA-IM) and broad changes in inflammation in the FGT (150), whereas others have found that general immunosuppressive effects correlated with DMPA-IM usage (167). Yet other studies have reported a correlation between DMPA-IM usage and a selective but not a broad proinflammatory effect (152, 159), unlike other studies reporting a predominantly and broad proinflammatory effect (162, 163). Specifically, DMPA-IM use has been associated with increased levels in vaginal fluids of selected proinflammatory cytokines in the FGT, including IL-6, IL-8, RANTES, MIP-1*α*, MIP-1*β*, and CXCL10 (162). In the same study, NET-EN users displayed significantly higher IL-6, IL-8, and RANTES concentrations. These results are partly consistent with a large study in Uganda and Zimbabwe that found significant elevation in vaginal fluids of RANTES for DMPA-IM users but no effect on IL-1*β*, IL-6, and IL-8, whereas the proinflammatory cytokines increased for COC (containing LNG) users and pregnant women (159). The results of these studies are particularly interesting in the context of the results of an *ex vivo* study showing that enhanced expression of inflammatory cytokines IL-1*β*, IL-6, and CXCL10 was associated with increased transcription of HIV-1 in ectocervical tissue explants and neutralization of IL-1*β* and IL-6 decreased viral transcription (316). Looking at the effect of HC on antimicrobial peptides, a study in Nairobi, Kenya, has shown that DMPA-IM use is associated with higher concentrations of cationic polypeptides, including HNP1, HNP2, HNP3, LL-37, and lactoferrin, possibly indicating neutrophil infiltration and increased inflammation (317).

In contrast to the previous studies showing a proinflammatory environment, a cross-sectional study in Alabama showed that women using DMPA-IM display an altered immune environment within the lower FGT characterized by decreased concentration of CXCL10, MCP-1, granulocyte colony-stimulating factor, and IFN-*α* in cervicovaginal lavages compared with controls (153). Another study by Ngcapu *et al.* (167) in KwaZulu-Natal, South Africa, concluded that injectable HC (DMPA-IM or NET-EN) use is associated with an immunosuppressive FGT innate immune profile characterized by lower levels in vaginal fluids of IL-12, IL-15, and MCP-1. However, when comparing results between studies for specific mediators, it is noteworthy that DMPA-IM usage has been associated with a decreased effect (152, 164), an increased effect (162, 163), and no effect (159) on IL-6 levels; a decreased effect (152), an increased effect (163), and no effect (159, 162) on IL-1*β* levels; and an increased effect (159, 162) and no associated changes for RANTES levels (152, 163) in DMPA-IM users or injectable users (167).

The concentration of immune mediators may differ substantially owing to population differences in the prevalence of suboptimal microbiota and bacterial, viral, or sexually transmitted protozoan infections. In support of this, the levels of several inflammatory cytokines, including IL-6, were increased in HC users with gonorrhea, chlamydia, or mycoplasma infections (167). The effects of HC on cervical immunity depend on the genital tract microenvironment (as discussed later in more detail in “HC, the Microbiome, and HIV-1 Acquisition”), and a weakened mucosal barrier against HIV may be a combined result of genital tract infections and HC use (160).

The concentration of soluble mediators may also vary depending on FGT compartment. Gene expression analysis of tissue from DMPA-IM and LNG intrauterine system users indicated increased expression of inflammatory genes in human endometrial tissue but not for immune function genes in cervical transformation zone tissue (166). Smith-McCune *et al.* (152) demonstrated increased endocervical concentrations of MCP-1 and IFN-*α* but decreased concentration of IL-1*β* and IL-6 in DMPA-IM users. Differences between these and other results (159, 162) may be because soluble mediator concentrations differ between endocervical and vaginal compartments. More studies are required to determine whether this is the case. The origin of immune mediators in the FGT fluid is not clearly defined in most studies. Whereas some mediators are produced by epithelial cells, others may originate from infiltrating activated immune cells in the lower or upper FGT. The concentration of any factor in FGT fluid is the result of a complex interplay between mucosal epithelium, number, type, and activation status of infiltrating immune cells, characteristics of vaginal microbiota, invading pathogens, and other environmental factors. These factors may result in discrepancies that are often observed between the effect of a progestin on the production of a particular immune mediator by a well-characterized population of cells *in vitro* and the effect of a contraceptive regimen on its concentration in the FGT.

Taken together, the reported effects of progestins on inflammation and levels of soluble immune modulators in the FGT exhibit a large degree of inconsistency. This may be due to differences in FGT compartment–specific effects or confounding factors such as age or the presence of other STIs or the microbiota. Despite attempts to correct for many of these confounding factors, there are still many inconsistencies between studies regarding quality (size, prospective vs cross-sectional, time span between measurements), which may contribute to the inconsistent results regarding immune modulators. Collectively, the data suggest that although MPA may have direct cell-specific and gene-specific effects on genes modulating inflammation in the FGT, the inflammatory profile as measured in clinical samples from women on DMPA-IM may vary due to multiple other indirect effects. Little information is available about the effects of other methods of HC besides injectables. *Ex vivo* studies suggest that MPA has differential effects on inflammation compared with NET and progesterone. Limited clinical studies suggest that COCs containing LNG result in a broadly proinflammatory environment, unlike DMPA-IM (159). Additional studies are required to determine whether specific forms of HC result in differential effects on inflammation *in vivo*, and whether these differ from those in the luteal phase. Increased inflammation *in vivo* may also be an indirect result of suppression of host immune responses to invading pathogens and may be observed only in specific populations. An inflammatory response may be exacerbated by the suppressive effect of a progestin on adaptive immune responses. The combined effects of HC and vaginal infection may lead to increased risk of HIV-1 due to inflammatory tissue damage and recruitment and activation of HIV-1 host cells (160).

“The current formulations of COC exert both estrogenic and progestogenic effects on endometrium.”

## HC, FGT Structure, Barrier Function, and HIV-1 Acquisition

Sex steroid hormones and HCs have a profound effect on both structure and function of the FGT because every cell type in the FGT expresses receptors for these hormones. Normal physiological changes observed during the menstrual cycle are regulated by estrogen and progesterone and are previously described in “The Female Genital Tract: Susceptibility to HIV-1 and Other STIs”. This section focuses specifically on the effects of HCs.

### Effects on the structure of the endometrium, cervix, and vagina

The exact effect of the exogenous hormones on the endometrial morphology most likely depends on the dose, duration, and type of progestin and whether it is given in combination with estrogen (176). Estradiol alone (1 mg of micronized E2 or 0.625 conjugated equine estrogens) was a commonly used hormone replacement therapy in the 1970s, before clinical studies showed a link between unopposed estradiol and endometrial cancer (318). Studies reported rates of 15% to 22% incidence of endometrial hyperplasia. Abnormal cytology and glandular structure in particular were linked to progression to carcinoma. Administration of a low dose of progestins in combination with estradiol appeared to negate this risk (318). The current formulations of COC exert both estrogenic and progestogenic effects on endometrium, with the progestin effect dominating owing to downregulation of ERs after several cycles of use (175). Consequently, in the first few cycles, characteristics of both proliferative and secretory effects are seen followed by atrophic changes in both glands and stroma after several cycles. The blood vessels also become thinner and sinusoidal with longer use. The most common formulation contained in oral progestin is NET and in implants it is ETG or LNG (28, 176), whereas in Southern Africa most COCs contain LNG (16). The changes in histology are similar for all of these progestins, with long-term use leading to atrophy of the endometrium, which includes small and sparse glands that have lost their tortuosity and are lined by low columnar cells (175, 176, 183). The stroma has large ovoid fibroblasts and the blood vessels are dilated and thin-walled, which can lead to breakthrough bleeding. In early cycles following use, endogenous estrogen may still be secreted, whereas progesterone secretion from the corpus luteum is shut down more rapidly. This results in slowing down of the changes seen in the secretory phase of the cycle, with underdevelopment of glands and stroma. Use of a progestin-only formulation such as DMPA-IM alone in women leads to altered cytology, including exaggerated glandular hyperplasia and decidualized stroma in early cycles following use (176). Following long-term use, the tissue histology changes to what is seen typically with most other progestins: endometrial atrophy and decreased density of dilated, thin-walled blood vessels, which can lead to breakthrough bleeding.

A number of studies have examined the effect of HC, especially COCs and DMPA-IM, on vaginal and cervical histology, epithelial thickness, and vaginal microflora (see “HC, the Microbiome, and HIV-1 Acquisition” later) (156–158, 319). In particular, the effect of DMPA-IM on vaginal histology has been examined by a number of studies because of the reports that have associated increased HIV-1 susceptibility and transmission in women using DMPA-IM (148). In most studies DMPA-IM did not affect the thickness of epithelium or number of LCs in the vaginal tissue, regardless of the length of use. There was also no change in cervical ectopy in studies regardless of the ethnicity of women using DMPA-IM.

Most of the information available on physical effects of HC is based mainly on COCs containing NET, LNG, and DMPA-IM, especially in the lower genital tract. Overall, the use of HC leads to thinning and atrophy of the endometrium; however, the effects are less obvious on vaginal morphology. Animal models, both NHP and rodent, do not simulate the effects of HC seen in women (see “Animal Studies Addressing the Effect of HC on the Acquisition of Genital Pathogens” previously).

### Effects on epithelial cells and barrier integrity

The epithelial mucosa lining the upper and lower genital tract provide the first barrier to the entry of sexually transmitted pathogenic organisms (222). The upper genital tract mucosa, which lines the endometrium and endocervix, is comprised of a monolayer of columnar epithelium, wherease the lower genital tract is lined by stratified squamous epithelium. The structure and physiology of these different types of epithelium were discussed earlier (see “The Female Genital Tract: Susceptibility to HIV-1 and Other STIs” previously). The columnar epithelium of the upper genital tract is primarily held together by apically located tight junctions, which essentially seal adjacent cells at their apical surface, preventing intercellular passage between cells [reviewed in Kaushic *et al.* (320)]. Below the tight junctions, adherens junctions are observed to maintain adhesion of adjacent cells, and finally desmosomes are found below the adherens junctions. The superficial layers of the pseudostratified squamous epithelium, alternatively, do not show any adhesion junctions, which are found primarily in the intermediate and basal cells (321). Adherens junctions are particularly abundant in these cells. The junctional integrity decreases progressively from basal to superficial layers. This correlates with increased permeability as noted by dye exclusion assays as well as the ability of labeled HIV to penetrate superficial layers.

Not much information is available regarding the direct effect of progestins on the mucosal barrier in the genital tract. A study of vaginal biopsies following 12 weeks of DMPA-IM use found no changes in the epithelial tight junction and adherens junction proteins by immunohistochemistry (148). However, another recent study that used proteomic analysis showed that DMPA-IM use is associated with innate immune activation and reduced epithelium repair pathways in the FGT (322). Another recent study in mice showed that DMPA and LNG downregulated vaginal cell-to-cell adhesion molecules, such as desmosomal cadherins DSG1*α* and desmocollin 1, and subsequently increased genital mucosal permeability, leading to increased endogenous microbial invasion from the lumen into the submucosal tissue, which was proposed to lead to increased vaginal inflammation when compared with untreated mice (133). Interestingly, administration of estrogen was able to reverse DMPA-mediated genital barrier disruption, highlighting the role of sex hormones in the maintenance of the mucosal barrier. The same study found that women initiating DMPA-IM use displayed significant reduced DSG1*α* and increased expression of selected proinflammatory markers (IL-1*β*, CD14, and CD177) in ectocervical tissues, similar to effects seen in the luteal phase of the menstrual cycle 1 month after use (133). Whether the effects seen for DMPA-IM are similar to those of P4 has not been examined. Using an *ex vivo* explant model, it was shown that the tissues from luteal phase were associated with increased HIV-1 acquisition (323). This is supported by a recent study where endometrial epithelial cells, cultured under hormonal conditions designed to mimic the luteal phase (E2 plus P4), showed increased permeability compared with those grown in E2 or vehicle alone (324). This increased leakage was implicated in allowing HIV access to stromal fibroblasts, which were seen to enhance HIV infection of CD4^+^ T cells. However, note that the progesterone levels used in this study (1 µM) are ∼20-fold higher than luteal phase progesterone and more consistent with pregnancy levels of progesterone. An association has also been indicated from clinical studies between the luteal phase and changes in proteins involved in tissue remodeling and leukocyte infiltration (325). These results suggest that some of the mechanisms associated with the luteal phase, when progesterone levels are higher during menstrual cycle, may be similar to those seen with HC. However, more studies are needed to confirm these initial reports.

## HC, the Microbiome, and HIV-1 Acquisition

There is increasing acknowledgment that the vaginal microbiome plays a critical role in maintaining homeostasis with the reproductive tract immune system and this, in turn, controls susceptibility to STIs, including HIV-1. However, much less is known about the development and maintenance of the vaginal microbiota. The squamous epithelium of the FGT produces a layer of glycogen under the influence of estrogen that provides a critical nutritional resource to the microbiota and clearly plays a critical role in shaping microbiota composition (326). The FGT is more dynamic than other mucosal sites because of menstruation, and consequently the vaginal microbiome is more dynamic than other mucosal microbiota. Using culture-independent methods (amplification and/or direct sequencing of marker genes such as 16S ribosomal RNA), studies have successfully identified >250 microbial species in the vaginal tract (327–330). Studies have also shown that the upper genital tract is not sterile; rather, it has a smaller diversity of bacterial species (331). Although the definition of “normal” and “healthy” vaginal microbiota is still debatable, genital microbiota in women can be identified into two groups based on clinical diagnosis: (1) microbiota that are considered to be healthy, and (2) bacteria linked to the clinical diagnosis of BV. Whereas a healthy microbiota is frequently dominated by *Lactobacillus* species, BV is characterized by a polymicrobial microbiota consisting of a variety of anaerobic bacteria and *Gardenella vaginalis*. Ravel *et al.* (329) used 16S rRNA sequencing of the vaginal microbiome from 396 asymptomatic North American women to define five consistent bacterial community state type. Of these, four were dominated by lactobacilli (*Lactobacillus iners*, *Lactobacillus crispatus*, *Lactobacillus gasseri*, and *Lactobacillus jensenii*), and a fifth group was not dominated by lactobacilli, but instead had mostly anaerobes and *G. vaginalis*. The latter was correlated with a high Nugent score (>4.5) in women, typically seen in women with symptomatic BV. Dysbiosis of vaginal microbiota, as in BV, has been linked to a 1.6- to 2-fold increased risk of acquisition of HSV-2, HIV, gonorrhea, and chlamydia (241, 332–334). Conversely, women with clinical BV have increased HSV-2 and HIV-1 replication and shedding (335, 336).

“Use of combined COC and a contraceptive vaginal ring has been shown to increase lactobacilli in the vaginal milieu.”

The association between BV and HIV-1 has been clearly shown by a number of studies. BV is known to increase the risk of HIV acquisition in women, and HIV-infected BV^+^ women are also threefold more likely to transmit HIV to their male partner than HIV-infected women who do not have BV (242, 337, 338). The underlying reason is likely due to local inflammatory processes that occur during BV. A recent study reported a potential mechanism underlying the strong *in vivo* relationship between high-diversity bacterial communities lacking *Lactobacillus* dominance and genital proinflammatory cytokine levels (339). Transcriptional analysis revealed that *Prevotella-*dominant communities contribute to genital inflammation *via* TLR4-mediated activation of NF-*κ*B and TNF-*α* signaling and recruitment of lymphocytes by chemokine production. This was accompanied by increased numbers of CCR5^+^CD4^+^ T cells in the endocervical canal, which suggests that high microbial diversity increases the likelihood that HIV will establish productive infection upon exposure. The ability of high microbial diversity in the genital tract to drive the production of inflammatory mediators also suggests that it has the capacity to induce genital barrier disruption and increase the likelihood of HIV transmission across the epithelium. Another recent study compared cervicovaginal proteome of 50 Rwandan female sex workers and correlated protein abundance based on increasing bacterial diversity, including *L. crispatus* dominant (group 1), *L. iners* dominant (group 2), moderate dysbiosis (group 3), and severe dysbiosis (group 4) (340). Dysbiosis was found to correlate with increased cervicovaginal inflammation and detrimental changes to the mucosal barrier.

There is increasing evidence that the mucosal microbiota modulates immune responses. The gut microbiota has been shown to regulate immune responses locally and systemically (341, 342) and modulate immune responses in allergy (343, 344). A recent review concluded that probiotics boosted antibody responses to oral vaccines against rotavirus, *Salmonella*, poliovirus, and *Vibrio cholerae* in adults and with childhood vaccines in infants (146). The mechanisms underlying the regulation of immune responses by microbiota are largely unknown. Even less is understood about the regulation of genital tract immunity by the vaginal microbiota, although it is thought to influence innate mucosal immunity in FRT (345). Certain anaerobic bacterial short chain fatty acids (*e.g.*, succinate) and exoenzymes (sialidases and proteases) have been shown to induce proinflammatory responses through TLR activation, DC activation and maturation, and immune cell migration and phagocytosis (345, 346). A recent study found that *Atopobium vaginae*, a bacteria associated with BV, induced proinflammatory cytokines from vaginal epithelial cells, whereas *L. cristpatus* did not (347). Clinically, successful treatment of BV is associated with decreases in both HIV target T cells in the cervix and activated CD4^+^ T cells in cervical cytobrush samples (348). There is a paucity of data describing clinical links between changes in microbiota and changes in immune cells in the genital tract. Overall, this area is substantially underinvestigated and represents a research gap.

There is clinical evidence, mostly from cross-sectional studies, linking contraceptive use with changes in vaginal microbiota. Two systemic reviews and a meta-analysis reported an overall decrease in BV with the use of HC (267, 349). The summary of the systemic reviews and some of the main studies since 2013 are presented in Table 6 (267, 293, 339, 349–352). A review of these studies shows that women using COC consistently show decreased risk of BV (Table 6). Data regarding progestin- containing HC such as DMPA-IM and other implantable HCs are often unclear because studies tend to group them together, even though they contain very different progestins (349). The systemic review by van de Wijgert *et al.* (267), which did compare DMPA and COC, concludes that both COC and DMPA-IM use are associated with a decrease in BV. However, two recent studies conducted in the FRESH cohort found no association between injectable contraceptive usage (76% DMPA-IM) and changes in microbiota in women aged 18 to 23 years, although these studies did not specifically examine BV status of women (150, 339).

**Table 6.** **Effect of Different Hormonal Contraceptives on Vaginal Microbiota**

**Study Details**	**Hormonal Exposure**	**Measurements**	**Summary of results**	**Reference**	**Note**
Systematic review and reanalysis of HC-HIV study of 36 eligible studies	Compared COC and DMPA-IM	BV based on Nugent score, vaginal candidiasis, KOH wet mount, and microbiome analysis by molecular techniques	Both COC and DMPA-IM reduce BV. The HC-HIV data show that COC and DMPA-IM reduce intermediate microflora. COC, but not DMPA-IM, increased vaginal candidiasis. Molecular vaginal microbiome studies showed that high E2 enhanced domination by *Lactobacillus* sp. P4 effects were not clear.	(267)*^a^*	
Systematic review and meta-analysis to examine association between HC and BV in 55 eligible studies	Examined effect of combined HC (COC and NuvaRing) and POC. POC included DMPA-IM, implants, injections, NET-EN, Mirena	Examined BV prevalence, incidence and recurrence as outcomes in eligible studies	HC was associated with both significantly reduced odds of prevalence and relative risk of incident and recurrent BV. Both combined HC and POC reduced BV.	(349)*^a^*	Analysis did not examine effect of DMPA-IM specifically; combined all POCs in the analysis
Longitudinal study that included 32 women at 12 mo and 22 women at 24 mo after treatment	Women using DMPA-IM for up to 2 y	Examined vaginal microbiota and mucosal immune populations by immunohistochemistry	Proportion of H_2_O_2_-positive lactobacilli decreased significantly over 12 mo.	(293)	
One hundred seventy-four HIV-negative Rwandan female sex workers followed for 2 y	Oral contraceptives or injectables	Examined STI prevalence and incidence and vaginal microbiota characterization by phylogenetic microarray	Oral and injectable contraceptive use was associated with a number of STIs; particularly, injectable contraceptives were associated with HSV-2, hormonal status was not correlated with Nugent score or vaginal microbiome clustering, and oral contraceptive users had lower BV-associated bacteria	(350)	
Cohort of young, black South African women (18–23 y) enrolled in FRESH Study	Women on no contraceptive (n = 48) were compared with women on DMPA-IM/Nuristerat (n = 43) or COC/non-HC (n = 9)	16S RNA sequencing to compare vaginal bacterial microflora	No differences were seen between bacterial communities in any of the groups	(339)	Study was not primarily designed to correlate bacterial abundance and community states with immune parameters, did not specifically determine Nugent score or BV
Cohort study of 248 women presenting for LNG-IUS insertion/reinsertion were stratified based on their current contraceptive method	No contraceptive, n = 72; LNG-IUS (Mirena), n = 88; COC, n = 64; oral progesterone-only pill/desogesterel implant (implanon), n = 18; Cu-IUD, n = 6	Menstrual pattern, medical history, vaginal microflora examined by phase-contrast microscopy, aerobic cultures to detect bacterial growth and fungal colonization	COC and LNG-IUS users had same vaginal microflora as non-contraceptive users. IUD users (hormonal and nonhormonal) had higher tendency for candida colonization, Women on progesterone-only pills/implants had lower candida but increased vaginal atrophy	(351)	Study did not use commonly used criteria, such as Nugent score or 16S sequencing
Retrospective study of 682 women, examining16S rRNA gene survey data from subset of participants from a larger microbiome project	Women using single form of birth control, either condoms, COC, DMPA-IM, or LNG-IUS	16S rRNA sequencing analysis to determine bacterial species, logistic regression models to determine H_2_O_2_-producing lactobacilli, correlation of lactobacilli to contraceptive method, age, ethnicity, STI status	Women using COC and DMPA-IM, but not LNG-IUS, were less likely to be colonized with BV-associated bacteria, compared with women who used condoms. COC users were more likely to be colonized by H_2_O_2_-producing lactobacilli, whereas women on DMPA-IM and LNG-IUS were not	(352)	

Abbreviations: IUS, intrauterine system; POC, progesterone-only contraceptive.

^*a*^ For a detailed list and outcomes of studies conducted prior to 2013, please refer to systematic reviews and meta-analyses in these references.

Despite the reported association between HC and reduced BV, the mechanism underlying this positive effect is unclear. The beneficial effect of DMPA-IM on BV remains controversial, as some studies have shown increased inflammation associated with MPA use (162, 164, 167). The inflammation caused by progestins could affect the genital barrier through indirect effects. Inflammation has been associated with increased permeability and decreased barrier function of epithelium in a number of studies. Some studies have reported a decrease in H_2_O_2_-producing lactobacilli in women using DMPA-IM (155, 293, 353). DMPA-IM was also shown to induce hypoestrogenism and slight thinning of glycogen-positive vaginal epithelium, which could potentially result in changes in vaginal microbiota away from lactobacilli-dominant to polymicrobial microflora consistent with BV (155).

Use of combined COC and a contraceptive vaginal ring has been shown to increase lactobacilli in the vaginal milieu (354, 355). In another study, following 2 months of COC use, vaginal microflora remained essentially unchanged except for a small decrease in H_2_O_2_-producing lactobacilli and in women with *Ureaplasma urealyticum* (319). Van de Wijgert *et al.* (267) reported an increased incidence of vaginal candidiasis in COC users. In nonhuman primates, similar to some clinical studies, no significant changes were noted in vaginal microflora following COC or DMPA-IM treatment (173).

Overall, the understanding of vaginal microbiome and its correlation with inflammation, HC, and HIV-1 acquisition is an area of enormous current interest. There is general agreement that changes in vaginal microbiome associated with inflammation lead to increased HIV-1 infection. Depending on the specific composition of the microbiota, changes in vaginal microflora may result in various levels of inflammation. Studies addressing the effect of HC on mucosal immune mediators may generate different results in populations that differ in the characteristics of microbiota and presence of cervicovaginal infections (160). Overall, studies to date generally show a correlation of HC with decreased BV. However, the underlying mechanism on how HCs change microbiome and/or inflammation is not as well understood and is an active area of investigation.

## Additional Controversies or Research Gaps

Several controversies and research gaps have been identified and discussed in previous sections. In this section, we discuss additional unresolved issues and important areas of investigation relevant to contraception and HIV-1 acquisition.

### Contraceptive efficacy, doses, and DMPA-IM vs DMPA-SC

Minimum or threshold doses of contraceptives required to maintain contraceptive efficacy have to our knowledge not been defined for most forms of HC. This poses a particular problem regarding side effects such as risks for HIV-1 acquisition. For some forms of HC, it may be possible to decrease side effects using lower doses while maintaining contraceptive efficacy. However, this requires an understanding of the relationship between dose and biological response for contraception and a particular side effect. More research is needed to define these relationships, in particular for MPA.

Studies addressing the benefits of the lower dose injectable DMPA-SC have convincingly demonstrated its important advantages of usability due to self-administration. DMPA-IM (150 mg) contains a higher dose of MPA than that required for contraceptive efficacy, because lowering the dose of MPA by 31% (from 150 mg to 104 mg) maintains contraceptive efficacy. Importantly, however, note that there is no evidence of a substantial advantage provided by the lower dose DMPA-SC over DMPA-IM (Depo-Provera) with respect to its biological properties, effects on the immune system, and side-effect risk profile. The assumption that lowering the dose by 31% will decrease side effects (62) is not based on evidence or sound theoretical considerations. It has not been established that the *C*_max_ serum levels of DMPA-IM and DMPA-SC are different, because no comparisons of these have been performed in the same study, and studies not performed in parallel have shown a wide degree of variability in measured *C*_max_ serum levels. In the one published study that did compare serum levels in parallel, the earliest measurement was at 6 months after the first administration (61). In this study the serum concentrations for DMPA-IM and DMPA-SC were not significantly different after 6 months, 1 year, or 2 years of usage, showing that the 31% lower dose does not significantly affect serum concentrations at these time points (61). Both DMPA-IM and DMPA-SC result in similar degrees of hypoestrogenism, with typical circulation estrogen concentrations of 10 to 100 pg/mL, which fluctuate widely in obese women for DMPA-SC (60, 62, 63, 150, 356). GCs have a well-established role in decreasing bone mineral density (140). Unlike other forms of HC, both DMPA-IM and DMPA-SC result in a similar transient decrease in bone mineral density (29, 61), which is likely due to the GC-like properties of MPA, acting via the GR (25, 357), as well as their hypoestrogenic effects (225). Taken together, the previous facts suggest that an increased risk of HIV-1 acquisition with DMPA-IM would most likely be similar to that of DMPA-SC, especially the underlying mechanisms that are mediated via the GR. The use of MPA doses <104 mg used in DMPA-SC is currently being explored in clinical trials for contraception efficacy (8). Detailed dose-titration studies using primary immune cells and relevant cell lines suggest that significant detectable changes in the effect of MPA and other progestins on immune mechanisms typically occur with 10- to 100-fold changes in concentration (100, 103, 109, 112, 115, 122, 123, 126, 129, 134). Regrettably, although Sayana Press provides substantially improved usability and important public health benefits, a 31% decrease in MPA dose is not likely to provide a substantial benefit in terms of ameliorating the negative effect of MPA on biological processes in women. Similar considerations need to be taken into account when lowering doses of any HC in an attempt to decrease side effects.

### Inflammation and Cu-IUDs

Insertion of a Cu-IUD into the uterine cavity is associated with local inflammatory reaction and recruitment of large numbers of neutrophils and macrophages from the stroma (358, 359). The Cu-IUD was shown to increase the concentration of granulocyte-macrophage colony-stimulating factor, soluble IL-2 receptor (soluble CD25), IL-1*β*, TNF-*α*, IL-6, and other cytokines/chemokines in the FGT fluid (360–362). Interestingly, a recent publication shows a positive correlation between the levels of IL-6, MCP-1, and MIP-1*α* and anti-HIV activity in the cervicovaginal lavage fluid of HIV-1–infected women (363). Based on this, it is feasible that anti-HIV activity is enhanced in FGT following insertion of Cu-IUD. However, the proinflammatory effect of Cu-IUD is also of concern as it may lead to increased recruitment and activation of HIV-1 target cells to genital epithelium and disruption of epithelial integrity that may facilitate HIV-1 transmission. It is critical to perform more research on the effect of Cu-IUDs and other types of IUDs and intravaginal implants *in vivo*. The effects of inflammation on combination therapies for intravaginal delivery involving antiretrovirals and progestins need to take into account the possible combined proinflammatory effects of these treatments.

“The strongest evidence for a possible biomarker for DMPA-IM is an increase in CCR5 levels or frequency of CCR5-expressing cells in the FGT.”

### Identification of biomarkers of increased susceptibility to HIV-1

Identification of biomarkers indicating increased susceptibility to viral infection as a result of contraceptive use would be highly beneficial. Although evidence for some potential biomarkers that indicate changes in immune function for a particular contraceptive *in vivo* is emerging, whether these have a causal relationship with changes in susceptibility remains to be established. Several potential biological markers have been identified for effects of DMPA-IM and other types of HC in women on soluble mediators and antiviral factors, in both the FGT and systemically, as discussed in “Effects on soluble antimicrobial factors” and “Effects on inflammation and levels of soluble immune modulators” previously. However, few of these are consistent between clinical studies, possibly due to multiple confounding factors. There is evidence from parallel studies that DMPA-IM has differential effects on some soluble mediators compared with other forms of HC (153, 166). Although there is strong evidence for an effect of MPA on the repression of several proinflammatory cytokine genes in *ex vivo* and animal models, the levels of consistency with clinical studies is variable. A decrease in SLPI with DMPA-IM has been observed in two clinical studies (159, 161), suggesting that it may be a possible biomarker for DMPA-IM effects. Other results suggest a decrease in HBD2 may be a biomarker for DMPA-IM and oral LNG (159, 165), although these results show inconsistency with other studies. Besides SLPI and HBD2, there are no reliable soluble mediator biomarkers for effects of a particular contraceptive on soluble mediators that have been validated in more than one clinical study for any method of HC. Some studies suggest that decreased cytokine levels but increased levels of chemokines such as RANTES and IL-8 may be biomarkers for effects of DMPA-IM (130, 159), although other studies are inconsistent with these data (152, 163). The finding that increased RANTES expression is associated with both DMPA-IM use and HIV-1 acquisition and shedding is particularly compelling (159). Additionally, there is evidence that a decrease in FGT permeability is associated with decreased expression of DSG1*α*, representing a possible biomarker, although this needs to be corroborated by future studies (133).

Potential biomarkers for the effect of DMPA-IM, consistent in two clinical reports, include markers for impairment of DC function, such as CD40 and CD80 (127, 153). To date, the strongest evidence for a possible biomarker for DMPA-IM is an increase in CCR5 levels or frequency of CCR5-expressing cells in the FGT, which has been observed in several clinical studies (133, 150, 153), but not with an LNG-releasing IUD (LNG-IUD) (151). However, this increase was not seen in the long-term DMPA-IM users (293). Critical assessment of the validity of individual biological markers that are modulated by HC in terms of their predictive value of the effect on HIV-1 transmission is hampered by the fact that our understanding of the heterosexual transmission of HIV-1 is rather limited. Despite important progress, precise correlates of protection against and susceptibility to HIV-1 infection are not clearly defined. Therefore, it is currently difficult to select a single biological effect of HC that would predict the probability HIV-1 transmission and acquisition.

### Disease progression and transmission

Multiple studies have addressed whether the use of HC during chronic HIV-1 infection affects the rate of viral replication and overall disease progression. Several studies reported a significantly higher HIV-1 viral load at the set point and acquisition of more diverse viral genotypes in women using DMPA-IM or COC at the time of HIV-1 infection compared with women without contraception (364, 365), consistent with the fact that viral load is highly predictive of HIV-1 disease progression (366, 367). However, other studies failed to confirm a relationship between the use of HC and acceleration of the disease (368–370), consistent with results for SIV in monkeys (169, 229, 232, 244). Several recent systematic reviews have concluded that HC methods do not appear to accelerate HIV disease progression as measured by mortality, time to initiation of antiretroviral therapy, an increase in HIV-RNA viral load, or a decrease in CD4 count (371–373). Although DMPA-IM is likely to increase HIV-1 acquisition, the effects of dampening systemic immune function on disease progression are difficult to predict and require further investigation (374). Some studies have indicated an association between contraception (DMPA or COC) and increased shedding of HIV-1 in the lower FGT with a significant dose dependency on progestin levels (370, 375, 376). Elevated levels of HIV-1 in the vaginal fluid may be responsible for a higher probability of women-to-men transmission, as indicated in some studies for DMPA-IM (377, 378). The clinical, animal, and *ex vivo* findings demonstrating the suppressive effect of DMPA on systemic immune function (112, 115, 122, 126, 128, 131, 153) suggest that disease progression may be affected in some women on DMPA-IM. The effects of dampening systemic immune function (374) on disease progression are difficult to predict and require further investigation.

### Public health

The most recent epidemiological data, together with animal and *ex vivo* data on MPA, raise important concerns about the use of DMPA in populations at high risk for HIV-1 acquisition. Importantly, withdrawal of DMPA from family planning programs without offering an equally effective form of contraception is not warranted, as it could result in a sharp increase in unintended pregnancies, unsafe abortions, and maternal and infant mortality and morbidity (15, 379, 380). It is therefore critical to focus on the identification of safe, effective, and affordable long-term methods of contraception that could effectively replace DMPA. Sufficient data on the effects on HIV-1 acquisition are not available for other available forms of HC, such as DMPA-SC or LNG-IUD. Replacement of DMPA with condoms is not a solution, as it would result in a substantial increase of unintended pregnancies and mortality due to high failure rates and absence of negotiation power by women (380). The Evidence for Contraceptive Options and HIV Outcomes trial will hopefully elucidate whether there is a significant difference in HIV-1 acquisition risk between DMPA-IM, LNG-sub dermal implant, or Cu-IUD; however it will not provide an answer regarding the risk relative to no contraception due to the absence of a control for no contraception. Any contraceptive that increases HIV-1 acquisition by 23% to 59% (HR 1.4) could potentially have a major impact on the HIV-1 epidemic, particularly in Southern and Eastern Africa (15). Based on the available clinical, *ex vivo*, and biochemical data, NET-EN represents a promising alternative option to DMPA until more data are available. There are no data suggesting NET-EN may pose a significantly higher risk than COCs or implants for HIV-1 acquisition. Furthermore, there is substantial evidence that NET exhibits differences in its mechanisms of action compared to MPA, that are likely to be relevant to HIV-1 acquisition.

## Conclusions

Most high-quality clinical observational studies find that the injectable progestin-only contraceptive DMPA-IM increases the likelihood of HIV-1 acquisition in women, unlike NET-EN and COCs containing LNG. These observations are consistent with the fact that individual progestins used in HC have differential biological effects via specific steroid receptors and that estrogen may exert a protective antiviral effect. They are also in accord with a large body of clinical, animal, and *ex vivo* data on the biological effects of MPA. Insufficient or no information is available regarding the potential effect of other forms of HC, including DMPA-SC, or non-HCs on HIV-1 acquisition. Further investigation is needed in these areas. There is substantial evidence for plausible biological mechanisms whereby MPA may increase HIV-1 acquisition. Current clinical, *ex vivo*, and animal model evidence supports a role for MPA in increasing the permeability of the FGT and promoting HIV-1 uptake. There is strong evidence from clinical, *ex vivo*, and mouse studies that MPA suppresses pDC and T cell function and suppresses select regulators of cellular and humoral systemic immunity (Fig. 3; Tables 3–5). Similarly, there is strong clinical and experimental animal data supporting a role for MPA in increasing the frequency of HIV-1 viral targets in the FGT and clinical and *ex vivo* evidence for increasing the levels of the CCR5 coreceptor for HIV-1 entry. Many of the previous mechanisms have been shown to occur in animal and *ex vivo* experiments at concentrations in the range of peak and plateau serum concentrations of MPA in DMPA-IM users. Although inconsistent, the clinical data suggest that DMPA-IM changes the expression of selected cytokines, chemokines, and innate defense factors in the FGT. Most clinical studies suggest that no major changes are exerted by DMPA-IM and other HCs on the microbiome; however, this requires further investigation. More consistent methodology, exclusion of possible confounding factors such as the characteristics of microbiota, and a focus on likely regulators of HIV-1 infection such as HBD2, SLPI, RANTES, and IFN-*α* may enhance the consistency between studies. Some of the effects of DMPA-IM may be due to the induction of hypoestrogenism; however, *ex vivo* data indicate an important role for a direct effect of MPA. Where different progestins have been compared in clinical and *ex vivo* human cell models, immunosuppressive effects were generally not observed with equimolar NET, progesterone, and LNG. Collectively, clinical, animal, and *ex vivo* studies are broadly consistent and suggest that DMPA may increase HIV-1 acquisition by increasing the permeability of the FGT, increasing transcytosis of the virus via the FGT, dampening select adaptive immune responses, increasing the frequency and infectability of HIV-1 target cells in the FGT, and increasing the rate of HIV-1 replication in target cells. Some of these effects do not occur with other progestins lacking GR activity or with luteal phase progesterone concentrations. *Ex vivo* data and receptor theory suggest that some of the effects of MPA on HIV-1 acquisition are likely to occur at MPA plateau concentrations and are not likely to be ameliorated by administration of DMPA-SC delivering a lower dose of MPA. The relative contribution of different mechanisms for MPA is unknown and requires further investigation. Taken together, the data provide a compelling case against the continuous use of DMPA in areas of high HIV-1 prevalence provided other forms of safe, affordable, non-HC or HC methods are readily available. Combined evidence suggests that a focus on methods not based on MPA or other progestins with GR activity should become a priority.

**Figure 4.**
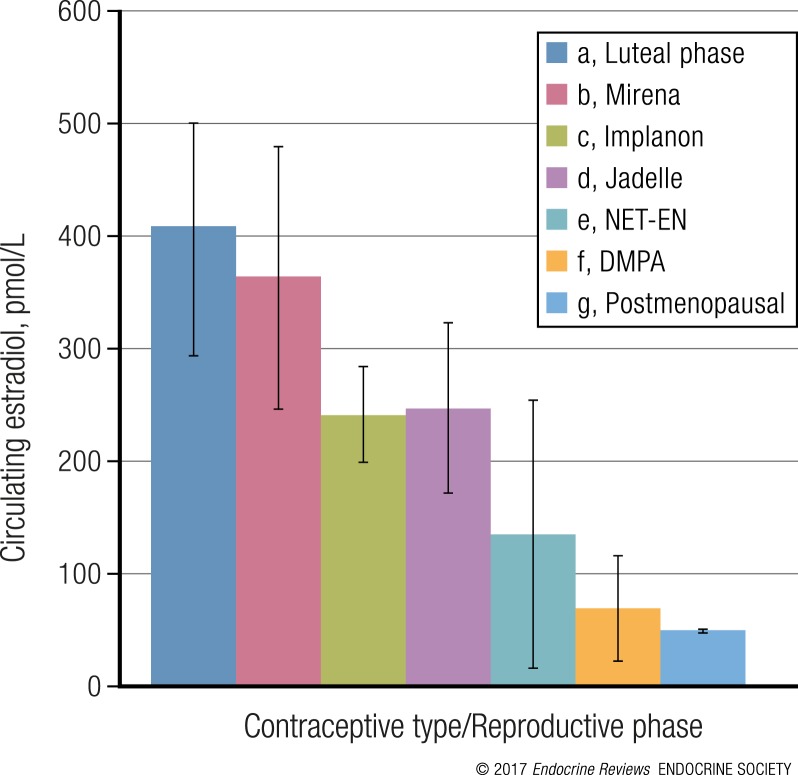
Mean circulating estradiol levels in premenopausal women on HCs. Mean circulating estradiol in (a) normal premenopausal contracepting women (N = 78); (b–f) premenopausal women using progestin-only contraceptives (b, Mirena, N = 8625; c, Implanon, N = 1126; d, Jadelle, N = 8827; e, NET-EN, N = 7323; f, DMPA, N = 31); and normal postmenopausal women (g, N = 1446). The graphs represent mean values with lines showing 95% confidence intervals (225). Reproduced with permission from Wolters Kluwer Health.

## References

[1] Sedgh G, Bearak J, Singh S, Bankole A, Popinchalk A, Ganatra B, Rossier C, Gerdts C, Tunçalp Ö, Johnson BR Jr, Johnston HB, Alkema L Abortion incidence between 1990 and 2014: global, regional, and subregional levels and trends. Lancet. 2016;388(10041):258–267. 27179755 10.1016/S0140-6736(16)30380-4 PMC5498988

[2] Sedgh G, Singh S, Hussain R Intended and unintended pregnancies worldwide in 2012 and recent trends. Stud Fam Plann. 2014;45(3):301–314. 25207494 10.1111/j.1728-4465.2014.00393.x PMC4727534

[3] United Nations Department of Economic and Social Affairs Population Division. Trends in contraceptive use worldwide 2015. Available at: http://www.un.org/en/development/desa/population/publications/pdf/family/trendsContraceptiveUse2015Report.pdf. Accessed on 31 December 2017.

[4] Polis CB, Curtis KM, Hannaford PC, Phillips SJ, Chipato T, Kiarie JN, Westreich DJ, Steyn PS An updated systematic review of epidemiological evidence on hormonal contraceptive methods and HIV acquisition in women. AIDS. 2016;30(17):2665–2683. 27500670 10.1097/QAD.0000000000001228 PMC5106090

[5] Hel Z, Stringer E, Mestecky J Sex steroid hormones, hormonal contraception, and the immunobiology of human immunodeficiency virus-1 infection. Endocr Rev. 2010;31(1):79–97. 19903932 10.1210/er.2009-0018 PMC2852204

[6] Murphy K, Irvin SC, Herold BC Research gaps in defining the biological link between HIV risk and hormonal contraception. Am J Reprod Immunol. 2014;72(2):228–235. 24548147 10.1111/aji.12209 PMC4106985

[7] Lipsitz R Women in India get a new birth control option. Available at: http://www.ourbodiesourselves.org/2016/03/women-in-india-get-a-new-birth-control-option/. Accessed on 31 December 2017.

[8] Schivone G, Dorflinger L, Halpern V Injectable contraception: updates and innovation. Curr Opin Obstet Gynecol. 2016;28(6):504–509. 27787287 10.1097/GCO.0000000000000329

[9] Pfizer. Novel agreement expands access to Pfizer’s contraceptive, Sayana® Press, for women most in need in the world’s poorest countries. Available at: http://www.familyplanning2020.org/articles/4458. Accessed on 31 December 2017.

[10] Whipkey K, Drake J, Kade K, Balderston B Advocacy pack for subcutaneous DMPA. Available at: https://www.rhsupplies.org/activities-resources/tools/advocacy-pack-for-subcutaneous-dmpa/. Accessed on 31 December 2017.

[11] Joint United Nations Programme on HIV/AIDS (UNAIDS). Fact sheet 2016. Available at: http://www.unaids.org/sites/default/files/media_asset/20150901_FactSheet_2015_en.pdf. Accessed 30 December 2017.

[12] GBD 2015 HIV Collaborators Estimates of global, regional, and national incidence, prevalence, and mortality of HIV, 1980-2015: the Global Burden of Disease Study 2015. Lancet HIV. 2016;3(8):e361–e387. 27470028 10.1016/S2352-3018(16)30087-X PMC5056319

[13] Avert. HIV around the world. Available at: http://www.avert.org/professionals/hiv-around-world. Accessed 30 December 2017.

[14] UNAIDS. South Africa: HIV and AIDS estimates. Available at: http://www.unaids.org/en/regionscountries/countries/southafrica. Accessed 30 December 2017.

[15] Butler AR, Smith JA, Polis CB, Gregson S, Stanton D, Hallett TB Modelling the global competing risks of a potential interaction between injectable hormonal contraception and HIV risk. AIDS. 2013;27(1):105–113. 23014519 10.1097/QAD.0b013e32835a5a52 PMC4862571

[16] Morrison CS, Chen PL, Kwok C, Baeten JM, Brown J, Crook AM, Van Damme L, Delany-Moretlwe S, Francis SC, Friedland BA, Hayes RJ, Heffron R, Kapiga S, Karim QA, Karpoff S, Kaul R, McClelland RS, McCormack S, McGrath N, Myer L, Rees H, van der Straten A, Watson-Jones D, van de Wijgert JH, Stalter R, Low N Hormonal contraception and the risk of HIV acquisition: an individual participant data meta-analysis. PLoS Med. 2015;12(1):e1001778. 25612136 10.1371/journal.pmed.1001778 PMC4303292

[17] Ralph LJ, Gollub EL, Jones HE Hormonal contraceptive use and women’s risk of HIV acquisition: priorities emerging from recent data. Curr Opin Obstet Gynecol. 2015;27(6):487–495. 26536211 10.1097/GCO.0000000000000228

[18] Noguchi LM, Richardson BA, Baeten JM, Hillier SL, Balkus JE, Chirenje ZM, Bunge K, Ramjee G, Nair G, Palanee-Phillips T, Selepe P, van der Straten A, Parikh UM, Gomez K, Piper JM, Watts DH, Marrazzo JM; VOICE Study Team Risk of HIV-1 acquisition among women who use different types of injectable progestin contraception in South Africa: a prospective cohort study. Lancet HIV. 2015;2(7):e279–e287. 26155597 10.1016/S2352-3018(15)00058-2 PMC4491329

[19] Heffron R, Parikh UM, Penrose KJ, Mugo N, Donnell D, Celum C, Mellors JW, Baeten JM; Partners PrEP Study Team Objective measurement of inaccurate condom use reporting among women using depot medroxyprogesterone acetate for contraception. AIDS Behav. 2017;21(7):2173–2179. 27699594 10.1007/s10461-016-1563-y PMC5378697

[20] Smith JA, Heffron R, Butler AR, Celum C, Baeten JM, Hallett TB Could misreporting of condom use explain the observed association between injectable hormonal contraceptives and HIV acquisition risk? Contraception. 2017;95(4):424–430. 28038949 10.1016/j.contraception.2016.12.003 PMC5387890

[21] McCoy SI, Ralph LJ, Padian NS, Minnis AM Are hormonal contraceptive users more likely to misreport unprotected sex? Evidence from a biomarker validation study in Zimbabwe. AIDS Behav. 2014;18(12):2259–2264. 24619603 10.1007/s10461-014-0741-z PMC4162861

[22] World Health Organization. Can women who are at high risk of acquiring HIV, safely use hormonal contraception? Available at: http://www.who.int/reproductivehealth/topics/family_planning/hormonal-contraception-hiv/en/. Accessed 30 December 2017.

[23] ECHO. The Evidence for Contraceptive Options and HIV Outcomes (ECHO) Study. Available at: http://echo-consortium.com/. Accessed 30 December 2017.

[24] Stanczyk FZ, Hapgood JP, Winer S, Mishell DR Jr Progestogens used in postmenopausal hormone therapy: differences in their pharmacological properties, intracellular actions, and clinical effects. Endocr Rev. 2013;34(2):171–208. 23238854 10.1210/er.2012-1008 PMC3610676

[25] Africander D, Verhoog N, Hapgood JP Molecular mechanisms of steroid receptor-mediated actions by synthetic progestins used in HRT and contraception. Steroids. 2011;76(7):636–652. 21414337 10.1016/j.steroids.2011.03.001

[26] Draper BH, Morroni C, Hoffman M, Smit J, Beksinska M, Hapgood J, Van der Merwe L Depot medroxyprogesterone versus norethisterone oenanthate for long-acting progestogenic contraception. Cochrane Database Syst Rev. 2006; (3):CD005214. 16856087 10.1002/14651858.CD005214.pub2 PMC11491191

[27] Sitruk-Ware R, Nath A, Mishell DR Jr Contraception technology: past, present and future. Contraception. 2013;87(3):319–330. 22995540 10.1016/j.contraception.2012.08.002 PMC3530627

[28] Jacobstein R, Polis CB Progestin-only contraception: injectables and implants. Best Pract Res Clin Obstet Gynaecol. 2014;28(6):795–806. 24996766 10.1016/j.bpobgyn.2014.05.003

[29] Dragoman MV, Gaffield ME The safety of subcutaneously administered depot medroxyprogesterone acetate (104 mg/0.65 mL): a systematic review. Contraception. 2016;94(3):202–215. 26874275 10.1016/j.contraception.2016.02.003

[30] Sitruk-Ware R, El-Etr M Progesterone and related progestins: potential new health benefits. Climacteric. 2013;16(Suppl 1):69–78. 23647429 10.3109/13697137.2013.802556

[31] Hapgood JP, Koubovec D, Louw A, Africander D Not all progestins are the same: implications for usage. Trends Pharmacol Sci. 2004;25(11):554–557. 15491776 10.1016/j.tips.2004.09.005

[32] Stanczyk FZ All progestins are not created equal. Steroids. 2003;68(10–13):879–890. 14667980 10.1016/j.steroids.2003.08.003

[33] Polis CB, Westreich D, Balkus JE, Heffron R; Participants of the 2013 HC-HIV Observational Analysis Meeting Assessing the effect of hormonal contraception on HIV acquisition in observational data: challenges and recommended analytic approaches. AIDS. 2013;27(Suppl 1):S35–S43. 24088682 10.1097/QAD.0000000000000036 PMC4153830

[34] Corsini G, Puppo F Effect of medroxyprogesterone acetate upon PHA, Con A and PWM stimulated lymphocytes and on E-rosette function. J Immunopharmacol. 1982–1983;4(3):247–253. 10.3109/08923978209026438 6223956

[35] Mallmann P, Dietrich K, Krebs D Effect of tamoxifen and high-dose medroxyprogesterone acetate (MPA) on cell-mediated immune functions in breast cancer patients. Methods Find Exp Clin Pharmacol. 1990;12(10):699–706. 2151629

[36] Gillgrass AE, Ashkar AA, Rosenthal KL, Kaushic C Prolonged exposure to progesterone prevents induction of protective mucosal responses following intravaginal immunization with attenuated herpes simplex virus type 2. J Virol. 2003;77(18):9845–9851. 12941893 10.1128/JVI.77.18.9845-9851.2003 PMC224606

[37] Trunova N, Tsai L, Tung S, Schneider E, Harouse J, Gettie A, Simon V, Blanchard J, Cheng-Mayer C Progestin-based contraceptive suppresses cellular immune responses in SHIV-infected rhesus macaques. Virology. 2006;352(1):169–177. 16730772 10.1016/j.virol.2006.04.004

[38] Kuhl H Pharmacology of progestogens. J Reproduktionsmed Endokrinol. 2011;8(1):157–176.

[39] Kuhl H Pharmacology of estrogens and progestogens: influence of different routes of administration. Climacteric. 2005;8(Suppl 1):3–63. 10.1080/13697130500148875 16112947

[40] Cherala G, Edelman A, Dorflinger L, Stanczyk FZ The elusive minimum threshold concentration of levonorgestrel for contraceptive efficacy. Contraception. 2016;94(2):104–108. 27000997 10.1016/j.contraception.2016.03.010

[41] Hidalgo MM, Hidalgo-Regina C, Bahamondes MV, Monteiro I, Petta CA, Bahamondes L Serum levonorgestrel levels and endometrial thickness during extended use of the levonorgestrel-releasing intrauterine system. Contraception. 2009;80(1):84–89. 19501221 10.1016/j.contraception.2009.01.004

[42] Thomas T, Petrie K, Shim J, Abildskov KM, Westhoff CL, Cremers S A UPLC-MS/MS method for therapeutic drug monitoring of etonogestrel. Ther Drug Monit. 2013;35(6):844–848. 24081205 10.1097/FTD.0b013e31829a10fa PMC3838448

[43] Pena MA, Sanz E, Francisco S, Alonso A, Abajo Z, Felipe I, Pascual J, Tost D, Bailac S Randomized, crossover and single-dose bioequivalence study of two oral desogestrel formulations (film-coated tablets of 75 μg) in healthy female volunteers. Sci Pharm. 2012;80(2):419–431. 22896827 10.3797/scipharm.1111-18 PMC3383212

[44] Shi YE, He CH, Gu J, Fotherby K Pharmacokinetics of norethisterone in humans. Contraception. 1987;35(5):465–475. 3621943 10.1016/0010-7824(87)90083-7

[45] Smit J, Botha J, McFadyen L, Beksinska M Serum medroxyprogesterone acetate levels in new and repeat users of depot medroxyprogesterone acetate at the end of the dosing interval. Contraception. 2004;69(1):3–7. 14720612 10.1016/j.contraception.2003.09.005

[46] U.S. Food and Drug Administration. Depo-SubQ Provera 104 (medroxyprogesterone acetate) injectable suspension. New drug application no. 021583. Center for Drug Evaluation and Research clinical pharmacology and biopharmaceutics review. Available at: https://www.accessdata.fda.gov/drugsatfda_docs/nda/2005/021584s000_depo-subQTOC.cfm. Accessed 31 December 2017.

[47] Mathrubutham M, Fotherby K Medroxyprogesterone acetate in human serum. J Steroid Biochem. 1981;14(8):783–786. 6457936 10.1016/0022-4731(81)90015-7

[48] Kirton KT, Cornette JC Return of ovulatory cyclicity following an intramuscular injection of medroxyprogesterone acetate (Provera). Contraception. 1974;10(1):39–45. 4442263 10.1016/0010-7824(74)90130-9

[49] Jeppsson S, Johansson ED Medroxyprogesterone acetate, estradiol, FSH and LH in peripheral blood after intramuscular administration of Depo-Provera to women. Contraception. 1976;14(4):461–469. 975831 10.1016/s0010-7824(76)80060-1

[50] Koetsawang S Injected long-acting medroxyprogesterone acetate. Effect on human lactation and concentrations in milk. J Med Assoc Thai. 1977;60(2):57–60. 559055

[51] Shrimanker K, Saxena BN, Fotherby K A radioimmunoassay for serum medroxyprogesterone acetate. J Steroid Biochem. 1978;9(4):359–363. 661315 10.1016/0022-4731(78)90631-3

[52] Fotherby K, Saxena BN, Shrimanker K, Hingorani V, Takker D, Diczfalusy E, Landgren BM A preliminary pharmacokinetic and pharmacodynamic evaluation of depot-medroxyprogesterone acetate and norethisterone oenanthate. Fertil Steril. 1980;34(2):131–139. 7409232 10.1016/s0015-0282(16)44895-8

[53] Fotherby K, Koetsawang S, Mathrubutham M Pharmacokinetic study of different doses of Depo Provera. Contraception. 1980;22(5):527–536. 6451351 10.1016/0010-7824(80)90105-5

[54] Jeppsson S, Gershagen S, Johansson ED, Rannevik G Plasma levels of medroxyprogesterone acetate (MPA), sex-hormone binding globulin, gonadal steroids, gonadotrophins and prolactin in women during long-term use of depo-MPA (Depo-Provera) as a contraceptive agent. Acta Endocrinol (Copenh). 1982;99(3):339–343. 6461995 10.1530/acta.0.0990339

[55] Fang SSD, Jiang H, Luo H Concentration changes if medroxyprogesterone acetate in serum and milk in lactating women who used depo geston. J Reprod Contraception. 2004;15:157–162.

[56] Bonny AE, Lange HL, Rogers LK, Gothard DM, Reed MD A pilot study of depot medroxyprogesterone acetate pharmacokinetics and weight gain in adolescent females. Contraception. 2014;89(5):357–360. 24582292 10.1016/j.contraception.2014.01.017 PMC4019679

[57] Nanda K, Callahan R, Taylor D, Wang M, Agot K, Jenkins D, Van Damme L, Dorflinger L; FEM-PrEP Study Group Medroxyprogesterone acetate levels among Kenyan women using depot medroxyprogesterone acetate in the FEM-PrEP trial. Contraception. 2016;94(1):40–47. 26972780 10.1016/j.contraception.2016.03.003 PMC4894753

[58] Ortiz A, Hirol M, Stanczyk FZ, Goebelsmann U, Mishell DR Serum medroxyprogesterone acetate (MPA) concentrations and ovarian function following intramuscular injection of depo-MPA. J Clin Endocrinol Metab. 1977;44(1):32–38. 833262 10.1210/jcem-44-1-32

[59] Virutamasen P, Leepipatpaiboon S, Kriengsinyot R, Vichaidith P, Muia PN, Sekadde-Kigondu CB, Mati JK, Forest MG, Dikkeschei LD, Wolthers BG, d’Arcangues C Pharmacodynamic effects of depot-medroxyprogesterone acetate (DMPA) administered to lactating women on their male infants. Contraception. 1996;54(3):153–157. 8899256 10.1016/s0010-7824(96)00170-9

[60] Mishell DR Jr Pharmacokinetics of depot medroxyprogesterone acetate contraception. J Reprod Med. 1996;41(5, Suppl):381–390. 8725700

[61] Kaunitz AM, Darney PD, Ross D, Wolter KD, Speroff L Subcutaneous DMPA vs. intramuscular DMPA: a 2-year randomized study of contraceptive efficacy and bone mineral density. Contraception. 2009;80(1):7–17. 19501210 10.1016/j.contraception.2009.02.005

[62] Jain J, Dutton C, Nicosia A, Wajszczuk C, Bode FR, Mishell DR Jr Pharmacokinetics, ovulation suppression and return to ovulation following a lower dose subcutaneous formulation of Depo-Provera. Contraception. 2004;70(1):11–18. 15208047 10.1016/j.contraception.2004.01.011

[63] Toh YC, Jain J, Rahnny MH, Bode FR, Ross D Suppression of ovulation by a new subcutaneous depot medroxyprogesterone acetate (104 mg/0.65 mL) contraceptive formulation in Asian women. Clin Ther. 2004;26(11):1845–1854. 15639696 10.1016/j.clinthera.2004.11.013

[64] Halpern V, Combes SL, Dorflinger LJ, Weiner DH, Archer DF Pharmacokinetics of subcutaneous depot medroxyprogesterone acetate injected in the upper arm. Contraception. 2014;89(1):31–35. 23993431 10.1016/j.contraception.2013.07.002

[65] Selim MF, Hussein AF Endothelial function in women using levonorgestrel-releasing intrauterine system (LNG-IUS). Contraception. 2013;87(4):396–403. 23332246 10.1016/j.contraception.2012.12.008

[66] Sivin I, Lähteenmäki P, Ranta S, Darney P, Klaisle C, Wan L, Mishell DR Jr, Lacarra M, Viegas OA, Bilhareus P, Koetsawang S, Piya-Anant M, Diaz S, Pavez M, Alvarez F, Brache V, LaGuardia K, Nash H, Stern J Levonorgestrel concentrations during use of levonorgestrel rod (LNG ROD) implants. Contraception. 1997;55(2):81–85. 9071516 10.1016/s0010-7824(96)00276-4

[67] Licea-Perez H, Wang S, Bowen CL, Yang E A semi-automated 96-well plate method for the simultaneous determination of oral contraceptives concentrations in human plasma using ultra performance liquid chromatography coupled with tandem mass spectrometry. J Chromatogr B Analyt Technol Biomed Life Sci. 2007;852(1–2):69–76. 10.1016/j.jchromb.2006.12.052 17258945

[68] Fotherby K, Koetsawang S Metabolism of injectable formulations of contraceptive steroids in obese and thin women. Contraception. 1982;26(1):51–58. 7128134 10.1016/0010-7824(82)90171-8

[69] Sang GW, Fotherby K, Howard G, Elder M, Bye PG Pharmacokinetics of norethisterone oenanthate in humans. Contraception. 1981;24(1):15–27. 7273765 10.1016/0010-7824(81)90065-2

[70] Goebelsmann U, Stanczyk FZ, Brenner PF, Goebelsmann AE, Gentzschein EK, Mishell DR Jr Serum norethindrone (NET) concentrations following intramuscular NET enanthate injection. Effect upon serum LH, FSH, estradiol and progesterone. Contraception. 1979;19(3):283–313. 572279 10.1016/0010-7824(79)90022-2

[71] Wenzl R, van Beek A, Schnabel P, Huber J Pharmacokinetics of etonogestrel released from the contraceptive implant Implanon. Contraception. 1998;58(5):283–288. 9883383 10.1016/s0010-7824(98)00110-3

[72] Mornar S, Chan LN, Mistretta S, Neustadt A, Martins S, Gilliam M Pharmacokinetics of the etonogestrel contraceptive implant in obese women. Am J Obstet Gynecol. 2012;207(2):110.e1-6. 22717269 10.1016/j.ajog.2012.05.002

[73] Kuhnz W, Schütt B, Power J, Back DJ Pharmacokinetics and serum protein binding of gestodene and 3-keto-desogestrel in women after single oral administration of two different contraceptive formulations. Arzneimittelforschung. 1992;42(9):1139–1141. 1445482

[74] Nanda K, Amaral E, Hays M, Viscola MA, Mehta N, Bahamondes L Pharmacokinetic interactions between depot medroxyprogesterone acetate and combination antiretroviral therapy. Fertil Steril. 2008;90(4):965–971. 17880953 10.1016/j.fertnstert.2007.07.1348

[75] Kärkkäinen J, Vesterinen E, Stenman UH, Adlercreutz H, Nieminen U, Widholm O Comparison of mass spectrometry and radioimmunoassay to measure medroxyprogesterone acetate in patients with endometrial cancer. Eur J Cancer. 1990;26(9):975–977. 2149024 10.1016/0277-5379(90)90624-3

[76] Keefe CC, Goldman MM, Zhang K, Clarke N, Reitz RE, Welt CK Simultaneous measurement of thirteen steroid hormones in women with polycystic ovary syndrome and control women using liquid chromatography-tandem mass spectrometry. PLoS One. 2014;9(4):e93805. 24713888 10.1371/journal.pone.0093805 PMC3979722

[77] Mould GP, Read J, Edwards D, Bye A A comparison of the high-performance liquid chromatography and RIA measurement of medroxyprogesterone acetate. J Pharm Biomed Anal. 1989;7(1):119–122. 2535119 10.1016/0731-7085(89)80072-x

[78] Canney PA, Dowsett M, Priestman TJ The pharmacokinetics of medroxyprogesterone acetate following two different loading dose schedules in advanced carcinoma of the breast. Br J Cancer. 1988;58(1):73–76. 2971386 10.1038/bjc.1988.165 PMC2246490

[79] Miller AA, Becher R, Schmidt CG Plasma concentrations of medroxyprogesterone acetate and megesterol acetate during long-term follow-up in patients treated for metastatic breast cancer. J Cancer Res Clin Oncol. 1988;114(2):186–190. 2965155 10.1007/BF00417835 PMC12243747

[80] Scambia G, Panici PB, Maccio A, Castelli P, Serri F, Mantovani G, Massidda B, Iacobelli S, Del Giacco S, Mancuso S Effects of antiestrogen and progestin on immune functions in breast cancer patients. Cancer. 1988;61(11):2214–2218. 2966668 10.1002/1097-0142(19880601)61:11<2214::aid-cncr2820611115>3.0.co;2-v

[81] Yamashita J, Hideshima T, Shirakusa T, Ogawa M Medroxyprogesterone acetate treatment reduces serum interleukin-6 levels in patients with metastatic breast carcinoma. Cancer. 1996;78(11):2346–2352. 8941005

[82] Vesterinen E, Backas NE, Pesonen K, Stenman UH, Laatikainen T Effect of medroxyprogesterone acetate on serum levels of LH, FSH, cortisol, and estrone in patients with endometrial carcinoma. Arch Gynecol. 1981;230(3):205–211. 6787991 10.1007/BF02111804

[83] Hedley DW, Christie M, Weatherby RP, Caterson ID Lack of correlations between plasma concentration of medroxyprogesterone acetate, hypothalamic-pituitary function, and tumour response in patients with advanced breast cancer. Cancer Chemother Pharmacol. 1985;14(2):112–115. 3156002 10.1007/BF00434347

[84] Polis CB, Phillips SJ, Hillier SL, Achilles SL Levonorgestrel in contraceptives and multipurpose prevention technologies: does this progestin increase HIV risk or interact with antiretrovirals? AIDS. 2016;30(17):2571–2576. 27525548 10.1097/QAD.0000000000001229 PMC5083202

[85] Thurman AR, Chandra N, Yousefieh N, Zalenskaya I, Kimble T, Asin S, Rollenhagen C, Anderson SM, Herold B, Mesquita PM, Richardson-Harman N, Cunningham T, Schwartz JL, Doncel GF Comparison of follicular and luteal phase mucosal markers of HIV susceptibility in healthy women. AIDS Res Hum Retroviruses. 2016;32(6):547–560. 26750085 10.1089/aid.2015.0264 PMC4892231

[86] Hafner LM, Cunningham K, Beagley KW Ovarian steroid hormones: effects on immune responses and *Chlamydia trachomatis* infections of the female genital tract. Mucosal Immunol. 2013;6(5):859–875. 23860476 10.1038/mi.2013.46

[87] Altemus M, Redwine L, Leong YM, Yoshikawa T, Yehuda R, Detera-Wadleigh S, Murphy DL Reduced sensitivity to glucocorticoid feedback and reduced glucocorticoid receptor mRNA expression in the luteal phase of the menstrual cycle. Neuropsychopharmacology. 1997;17(2):100–109. 9252985 10.1016/S0893-133X(97)00039-0

[88] Hapgood JP, Africander D, Louw R, Ray RM, Rohwer JM Potency of progestogens used in hormonal therapy: toward understanding differential actions. J Steroid Biochem Mol Biol. 2014;142:39–47. 23954501 10.1016/j.jsbmb.2013.08.001

[89] Hapgood JP, Avenant C, Moliki JM Glucocorticoid-independent modulation of GR activity: implications for immunotherapy. Pharmacol Ther. 2016;165:93–113. 27288728 10.1016/j.pharmthera.2016.06.002 PMC5195849

[90] Bennesch MA, Picard D Minireview: tipping the balance: ligand-independent activation of steroid receptors. Mol Endocrinol. 2015;29(3):349–363. 25625619 10.1210/me.2014-1315 PMC5414756

[91] Levin ER, Hammes SR Nuclear receptors outside the nucleus: extranuclear signalling by steroid receptors. Nat Rev Mol Cell Biol. 2016;17(12):783–797. 27729652 10.1038/nrm.2016.122 PMC5649368

[92] Cruz-Topete D, Cidlowski JA One hormone, two actions: anti- and pro-inflammatory effects of glucocorticoids. Neuroimmunomodulation. 2015;22(1–2):20–32. 25227506 10.1159/000362724 PMC4243162

[93] Mohammed H, Russell IA, Stark R, Rueda OM, Hickey TE, Tarulli GA, Serandour AA, Birrell SN, Bruna A, Saadi A, Menon S, Hadfield J, Pugh M, Raj GV, Brown GD, D’Santos C, Robinson JL, Silva G, Launchbury R, Perou CM, Stingl J, Caldas C, Tilley WD, Carroll JS Progesterone receptor modulates ERα action in breast cancer. Nature. 2015;523(7560):313–317. 26153859 10.1038/nature14583 PMC4650274

[94] Dahm AE, Eilertsen AL, Goeman J, Olstad OK, Ovstebø R, Kierulf P, Mowinckel MC, Skretting G, Sandset PM A microarray study on the effect of four hormone therapy regimens on gene transcription in whole blood from healthy postmenopausal women. Thromb Res. 2012;130(1):45–51. 22217510 10.1016/j.thromres.2011.12.009

[95] Kayisli UA, Basar M, Guzeloglu-Kayisli O, Semerci N, Atkinson HC, Shapiro J, Summerfield T, Huang SJ, Prelle K, Schatz F, Lockwood CJ Long-acting progestin-only contraceptives impair endometrial vasculature by inhibiting uterine vascular smooth muscle cell survival. Proc Natl Acad Sci USA. 2015;112(16):5153–5158. 25847994 10.1073/pnas.1424814112 PMC4413279

[96] Guzeloglu Kayisli O, Kayisli UA, Basar M, Semerci N, Schatz F, Lockwood CJ Progestins upregulate FKBP51 expression in human endometrial stromal cells to induce functional progesterone and glucocorticoid withdrawal: implications for contraceptive-associated abnormal uterine bleeding. PLoS One. 2015;10(10):e0137855. 26436918 10.1371/journal.pone.0137855 PMC4593551

[97] Ghatge RP, Jacobsen BM, Schittone SA, Horwitz KB The progestational and androgenic properties of medroxyprogesterone acetate: gene regulatory overlap with dihydrotestosterone in breast cancer cells. Breast Cancer Res. 2005;7(6):R1036–R1050. 16457685 10.1186/bcr1340 PMC1410743

[98] Lei K, Chen L, Georgiou EX, Sooranna SR, Khanjani S, Brosens JJ, Bennett PR, Johnson MR Progesterone acts via the nuclear glucocorticoid receptor to suppress IL-1β-induced COX-2 expression in human term myometrial cells. PLoS One. 2012;7(11):e50167. 23209664 10.1371/journal.pone.0050167 PMC3509141

[99] Tan H, Yi L, Rote NS, Hurd WW, Mesiano S Progesterone receptor-A and -B have opposite effects on proinflammatory gene expression in human myometrial cells: implications for progesterone actions in human pregnancy and parturition. J Clin Endocrinol Metab. 2012;97(5):E719–E730. 22419721 10.1210/jc.2011-3251 PMC3339884

[100] Ronacher K, Hadley K, Avenant C, Stubsrud E, Simons SS Jr, Louw A, Hapgood JP Ligand-selective transactivation and transrepression via the glucocorticoid receptor: role of cofactor interaction. Mol Cell Endocrinol. 2009;299(2):219–231. 19007848 10.1016/j.mce.2008.10.008

[101] Africander D, Louw R, Hapgood JP Investigating the anti-mineralocorticoid properties of synthetic progestins used in hormone therapy. Biochem Biophys Res Commun. 2013;433(3):305–310. 23473756 10.1016/j.bbrc.2013.02.086

[102] Africander DJ, Storbeck KH, Hapgood JP A comparative study of the androgenic properties of progesterone and the progestins, medroxyprogesterone acetate (MPA) and norethisterone acetate (NET-A). J Steroid Biochem Mol Biol. 2014;143:404–415. 24861265 10.1016/j.jsbmb.2014.05.007

[103] Koubovec D, Ronacher K, Stubsrud E, Louw A, Hapgood JP Synthetic progestins used in HRT have different glucocorticoid agonist properties. Mol Cell Endocrinol. 2005;242(1–2):23–32. 16125839 10.1016/j.mce.2005.07.001

[104] Koubovec D, Vanden Berghe W, Vermeulen L, Haegeman G, Hapgood JP Medroxyprogesterone acetate downregulates cytokine gene expression in mouse fibroblast cells. Mol Cell Endocrinol. 2004;221(1–2):75–85. 15223134 10.1016/j.mce.2004.03.006

[105] Chu MC, Zhang X, Gentzschein E, Stanczyk FZ, Lobo RA Formation of ethinyl estradiol in women during treatment with norethindrone acetate. J Clin Endocrinol Metab. 2007;92(6):2205–2207. 17341557 10.1210/jc.2007-0044

[106] Kuhl H, Wiegratz I Can 19-nortestosterone derivatives be aromatized in the liver of adult humans? Are there clinical implications? Climacteric. 2007;10(4):344–353. 17653961 10.1080/13697130701380434

[107] Chwalisz K, Surrey E, Stanczyk FZ The hormonal profile of norethindrone acetate: rationale for add-back therapy with gonadotropin-releasing hormone agonists in women with endometriosis. Reprod Sci. 2012;19(6):563–571. 22457429 10.1177/1933719112438061

[108] Kuhnz W, Heuner A, Hümpel M, Seifert W, Michaelis K In vivo conversion of norethisterone and norethisterone acetate to ethinyl etradiol in postmenopausal women. Contraception. 1997;56(6):379–385. 9494772 10.1016/s0010-7824(97)00174-1

[109] Louw-du Toit R, Hapgood JP, Africander D Medroxyprogesterone acetate differentially regulates interleukin (IL)-12 and IL-10 in a human ectocervical epithelial cell line in a glucocorticoid receptor (GR)-dependent manner. J Biol Chem. 2014;289(45):31136–31149. 25202013 10.1074/jbc.M114.587311 PMC4223317

[110] Giannoukos G, Szapary D, Smith CL, Meeker JE, Simons SS Jr New antiprogestins with partial agonist activity: potential selective progesterone receptor modulators (SPRMs) and probes for receptor- and coregulator-induced changes in progesterone receptor induction properties. Mol Endocrinol. 2001;15(2):255–270. 11158332 10.1210/mend.15.2.0596

[111] Sasagawa S, Shimizu Y, Kami H, Takeuchi T, Mita S, Imada K, Kato S, Mizuguchi K Dienogest is a selective progesterone receptor agonist in transactivation analysis with potent oral endometrial activity due to its efficient pharmacokinetic profile. Steroids. 2008;73(2):222–231. 18061638 10.1016/j.steroids.2007.10.003

[112] Kleynhans L, Du Plessis N, Black GF, Loxton AG, Kidd M, van Helden PD, Walzl G, Ronacher K Medroxyprogesterone acetate alters *Mycobacterium bovis* BCG-induced cytokine production in peripheral blood mononuclear cells of contraceptive users. PLoS One. 2011;6(9):e24639. 21931790 10.1371/journal.pone.0024639 PMC3169620

[113] Chow CC, Ong KM, Dougherty EJ, Simons SS Jr Inferring mechanisms from dose-response curves. Methods Enzymol. 2011;487:465–483. 21187235 10.1016/B978-0-12-381270-4.00016-0 PMC3177954

[114] Bamberger CM, Else T, Bamberger AM, Beil FU, Schulte HM Dissociative glucocorticoid activity of medroxyprogesterone acetate in normal human lymphocytes. J Clin Endocrinol Metab. 1999;84(11):4055–4061. 10566649 10.1210/jcem.84.11.6091

[115] Govender Y, Avenant C, Verhoog NJ, Ray RM, Grantham NJ, Africander D, Hapgood JP The injectable-only contraceptive medroxyprogesterone acetate, unlike norethisterone acetate and progesterone, regulates inflammatory genes in endocervical cells via the glucocorticoid receptor. PLoS One. 2014;9(5):e96497. 24840644 10.1371/journal.pone.0096497 PMC4026143

[116] Zhao Q, Pang J, Favata MF, Trzaskos JM Receptor density dictates the behavior of a subset of steroid ligands in glucocorticoid receptor-mediated transrepression. Int Immunopharmacol. 2003;3(13–14):1803–1817. 14636830 10.1016/j.intimp.2003.08.005

[117] Robertson S, Rohwer JM, Hapgood JP, Louw A Impact of glucocorticoid receptor density on ligand-independent dimerization, cooperative ligand-binding and basal priming of transactivation: a cell culture model. PLoS One. 2013;8(5):e64831. 23717665 10.1371/journal.pone.0064831 PMC3661511

[118] Bray JD, Jelinsky S, Ghatge R, Bray JA, Tunkey C, Saraf K, Jacobsen BM, Richer JK, Brown EL, Winneker RC, Horwitz KB, Lyttle CR Quantitative analysis of gene regulation by seven clinically relevant progestins suggests a highly similar mechanism of action through progesterone receptors in T47D breast cancer cells. J Steroid Biochem Mol Biol. 2005;97(4):328–341. 16157482 10.1016/j.jsbmb.2005.06.032

[119] Beasley A, White KO, Cremers S, Westhoff C Randomized clinical trial of self versus clinical administration of subcutaneous depot medroxyprogesterone acetate. Contraception. 2014;89(5):352–356. 24656555 10.1016/j.contraception.2014.01.026 PMC4086940

[120] Fuhrmann U, Slater EP, Fritzemeier KH Characterization of the novel progestin gestodene by receptor binding studies and transactivation assays. Contraception. 1995;51(1):45–52. 7750284 10.1016/0010-7824(94)00003-f

[121] Kontula K, Paavonen T, Luukkainen T, Andersson LC Binding of progestins to the glucocorticoid receptor. Correlation to their glucocorticoid-like effects on in vitro functions of human mononuclear leukocytes. Biochem Pharmacol. 1983;32(9):1511–1518. 6222739 10.1016/0006-2952(83)90474-4

[122] Huijbregts RP, Helton ES, Michel KG, Sabbaj S, Richter HE, Goepfert PA, Hel Z Hormonal contraception and HIV-1 infection: medroxyprogesterone acetate suppresses innate and adaptive immune mechanisms. Endocrinology. 2013;154(3):1282–1295. 23354099 10.1210/en.2012-1850 PMC3578997

[123] Hapgood JP, Ray RM, Govender Y, Avenant C, Tomasicchio M Differential glucocorticoid receptor-mediated effects on immunomodulatory gene expression by progestin contraceptives: implications for HIV-1 pathogenesis. Am J Reprod Immunol. 2014;71(6):505–512. 24547700 10.1111/aji.12214

[124] Irvin SC, Herold BC Molecular mechanisms linking high dose medroxyprogesterone with HIV-1 risk. PLoS One. 2015;10(3):e0121135. 25798593 10.1371/journal.pone.0121135 PMC4370479

[125] Hapgood JP Immunosuppressive biological mechanisms support reassessment of use of the injectable contraceptive medroxyprogesterone acetate. Endocrinology. 2013;154(3):985–988. 23429710 10.1210/en.2013-1066

[126] Huijbregts RP, Michel KG, Hel Z Effect of progestins on immunity: medroxyprogesterone but not norethisterone or levonorgestrel suppresses the function of T cells and pDCs. Contraception. 2014;90(2):123–129. 24674041 10.1016/j.contraception.2014.02.006 PMC4874781

[127] Quispe Calla NE, Ghonime MG, Cherpes TL, Vicetti Miguel RD Medroxyprogesterone acetate impairs human dendritic cell activation and function. Hum Reprod. 2015;30(5):1169–1177. 25740884 10.1093/humrep/dev035 PMC4481667

[128] Tomasicchio M, Avenant C, Du Toit A, Ray RM, Hapgood JP The progestin-only contraceptive medroxyprogesterone acetate, but not norethisterone acetate, enhances HIV-1 Vpr-mediated apoptosis in human CD4^+^ T cells through the glucocorticoid receptor. PLoS One. 2013;8(5):e62895. 23658782 10.1371/journal.pone.0062895 PMC3643923

[129] Sampah ME, Laird GM, Blankson JN, Siliciano RF, Coleman JS Medroxyprogesterone acetate increases HIV-1 infection of unstimulated peripheral blood mononuclear cells in vitro. AIDS. 2015;29(10):1137–1146. 26035316 10.1097/QAD.0000000000000681 PMC4453018

[130] Ferreira VH, Dizzell S, Nazli A, Kafka JK, Mueller K, Nguyen PV, Tremblay MJ, Cochrane A, Kaushic C Medroxyprogesterone acetate regulates HIV-1 uptake and transcytosis but not replication in primary genital epithelial cells, resulting in enhanced T-cell infection. J Infect Dis. 2015;211(11):1745–1756. 25538276 10.1093/infdis/jiu832

[131] Kleynhans L, Du Plessis N, Allie N, Jacobs M, Kidd M, van Helden PD, Walzl G, Ronacher K The contraceptive depot medroxyprogesterone acetate impairs mycobacterial control and inhibits cytokine secretion in mice infected with *Mycobacterium tuberculosis*. Infect Immun. 2013;81(4):1234–1244. 23381991 10.1128/IAI.01189-12 PMC3639598

[132] Vicetti Miguel RD, Hendricks RL, Aguirre AJ, Melan MA, Harvey SA, Terry-Allison T, St Leger AJ, Thomson AW, Cherpes TL Dendritic cell activation and memory cell development are impaired among mice administered medroxyprogesterone acetate prior to mucosal herpes simplex virus type 1 infection. J Immunol. 2012;189(7):3449–3461. 22942424 10.4049/jimmunol.1103054 PMC3448807

[133] Quispe Calla NE, Vicetti Miguel RD, Boyaka PN, Hall-Stoodley L, Kaur B, Trout W, Pavelko SD, Cherpes TL Medroxyprogesterone acetate and levonorgestrel increase genital mucosal permeability and enhance susceptibility to genital herpes simplex virus type 2 infection. Mucosal Immunol. 2016;9(6):1571–1583. 27007679 10.1038/mi.2016.22 PMC5035233

[134] Radzio J, Hanley K, Mitchell J, Ellis S, Deyounks F, Jenkins LT, Hanson D, Heneine W, García-Lerma JG Physiologic doses of depot-medroxyprogesterone acetate do not increase acute plasma simian HIV viremia or mucosal virus shedding in pigtail macaques. AIDS. 2014;28(10):1431–1439. 24759208 10.1097/QAD.0000000000000294

[135] Butler K, Ritter JM, Ellis S, Morris MR, Hanson DL, McNicholl JM, Kersh EN A depot medroxyprogesterone acetate dose that models human use and its effect on vaginal SHIV acquisition risk. J Acquir Immune Defic Syndr. 2016;72(4):363–371. 27355414 10.1097/QAI.0000000000000975 PMC4930010

[136] Baschant U, Tuckermann J The role of the glucocorticoid receptor in inflammation and immunity. J Steroid Biochem Mol Biol. 2010;120(2–3):69–75. 20346397 10.1016/j.jsbmb.2010.03.058

[137] Chinenov Y, Coppo M, Gupte R, Sacta MA, Rogatsky I Glucocorticoid receptor coordinates transcription factor-dominated regulatory network in macrophages. BMC Genomics. 2014;15(1):656. 25099603 10.1186/1471-2164-15-656 PMC4133603

[138] Chinenov Y, Gupte R, Rogatsky I Nuclear receptors in inflammation control: repression by GR and beyond. Mol Cell Endocrinol. 2013;380(1–2):55–64. 23623868 10.1016/j.mce.2013.04.006 PMC3787948

[139] Busillo JM, Cidlowski JA The five Rs of glucocorticoid action during inflammation: ready, reinforce, repress, resolve, and restore. Trends Endocrinol Metab. 2013;24(3):109–119. 23312823 10.1016/j.tem.2012.11.005 PMC3667973

[140] Frenkel B, White W, Tuckermann J Glucocorticoid-induced osteoporosis. Adv Exp Med Biol. 2015;872:179–215. 26215995 10.1007/978-1-4939-2895-8_8 PMC5905346

[141] Padgett DA, Glaser R How stress influences the immune response. Trends Immunol. 2003;24(8):444–448. 12909458 10.1016/s1471-4906(03)00173-x

[142] Ng SS, Li A, Pavlakis GN, Ozato K, Kino T Viral infection increases glucocorticoid-induced interleukin-10 production through ERK-mediated phosphorylation of the glucocorticoid receptor in dendritic cells: potential clinical implications. PLoS One. 2013;8(5):e63587. 23667643 10.1371/journal.pone.0063587 PMC3648469

[143] Hapgood JP, Tomasicchio M Modulation of HIV-1 virulence via the host glucocorticoid receptor: towards further understanding the molecular mechanisms of HIV-1 pathogenesis. Arch Virol. 2010;155(7):1009–1019. 20446002 10.1007/s00705-010-0678-0

[144] Quispe Calla NE, Vicetti Miguel RD, Mei A, Fan S, Gilmore JR, Cherpes TL Dendritic cell function and pathogen-specific T cell immunity are inhibited in mice administered levonorgestrel prior to intranasal *Chlamydia trachomatis* infection. Sci Rep. 2016;6(1):37723. 27892938 10.1038/srep37723 PMC5125275

[145] Wira C, Crane-Godreau A, Grant KS Endocrine regulation of the mucosal immune system in the female reproductive tract In: Mestecky J, Lamm ME, Strober W, Bienenstock J, McGhee JR, Mayer L, eds. *Mucosal Immunol.* 3rd ed. New York, NY: Elsevier Academic Press; 2005:1661–1676.

[146] Ferreira RB, Antunes LC, Finlay BB Should the human microbiome be considered when developing vaccines? PLoS Pathog. 2010;6(11):e1001190. 21124987 10.1371/journal.ppat.1001190 PMC2987818

[147] Sciaranghella G, Wang C, Hu H, Anastos K, Merhi Z, Nowicki M, Stanczyk FZ, Greenblatt RM, Cohen M, Golub ET, Watts DH, Alter G, Young MA, Tsibris AM CCR5 expression levels in HIV-uninfected women receiving hormonal contraception. J Infect Dis. 2015;212(9):1397–1401. 25895986 10.1093/infdis/jiv233 PMC4601918

[148] Chandra N, Thurman AR, Anderson S, Cunningham TD, Yousefieh N, Mauck C, Doncel GF Depot medroxyprogesterone acetate increases immune cell numbers and activation markers in human vaginal mucosal tissues. AIDS Res Hum Retroviruses. 2013;29(3):592–601. 23189932 10.1089/aid.2012.0271 PMC3581024

[149] Prakash M, Kapembwa MS, Gotch F, Patterson S Oral contraceptive use induces upregulation of the CCR5 chemokine receptor on CD4^+^ T cells in the cervical epithelium of healthy women. J Reprod Immunol. 2002;54(1–2):117–131. 11839399 10.1016/s0165-0378(01)00125-5

[150] Byrne EH, Anahtar MN, Cohen KE, Moodley A, Padavattan N, Ismail N, Bowman BA, Olson GS, Mabhula A, Leslie A, Ndung’u T, Walker BD, Ghebremichael MS, Dong KL, Kwon DS Association between injectable progestin-only contraceptives and HIV acquisition and HIV target cell frequency in the female genital tract in South African women: a prospective cohort study. Lancet Infect Dis. 2016;16(4):441–448. 26723758 10.1016/S1473-3099(15)00429-6 PMC5294917

[151] Achilles SL, Creinin MD, Stoner KA, Chen BA, Meyn L, Hillier SL Changes in genital tract immune cell populations after initiation of intrauterine contraception. Am J Obstet Gynecol. 2014;211(5):489.e1-9. 24834865 10.1016/j.ajog.2014.05.016 PMC4231025

[152] Smith-McCune KK, Hilton JF, Shanmugasundaram U, Critchfield JW, Greenblatt RM, Seidman D, Averbach S, Giudice LC, Shacklett BL Effects of depot-medroxyprogesterone acetate on the immune microenvironment of the human cervix and endometrium: implications for HIV susceptibility. Mucosal Immunol. 2017;10(5):1270–1278. 28051087 10.1038/mi.2016.121 PMC5496803

[153] Michel KG, Huijbregts RP, Gleason JL, Richter HE, Hel Z Effect of hormonal contraception on the function of plasmacytoid dendritic cells and distribution of immune cell populations in the female reproductive tract. J Acquir Immune Defic Syndr. 2015;68(5):511–518. 25763784 10.1097/QAI.0000000000000531 PMC4874780

[154] Hughes GC, Thomas S, Li C, Kaja MK, Clark EA Cutting edge: progesterone regulates IFN-α production by plasmacytoid dendritic cells. J Immunol. 2008;180(4):2029–2033. 18250406 10.4049/jimmunol.180.4.2029

[155] Miller L, Patton DL, Meier A, Thwin SS, Hooton TM, Eschenbach DA Depomedroxyprogesterone-induced hypoestrogenism and changes in vaginal flora and epithelium. Obstet Gynecol. 2000;96(3):431–439. 10960638 10.1016/s0029-7844(00)00906-6

[156] Bahamondes MV, Castro S, Marchi NM, Marcovici M, Andrade LA, Fernandes A, Bahamondes L Human vaginal histology in long-term users of the injectable contraceptive depot-medroxyprogesterone acetate. Contraception. 2014;90(2):117–122. 24613369 10.1016/j.contraception.2014.01.024

[157] Mauck CK, Callahan MM, Baker J, Arbogast K, Veazey R, Stock R, Pan Z, Morrison CS, Chen-Mok M, Archer DF, Gabelnick HL The effect of one injection of Depo-Provera on the human vaginal epithelium and cervical ectopy. Contraception. 1999;60(1):15–24. 10549448 10.1016/s0010-7824(99)00058-x

[158] Bahamondes L, Trevisan M, Andrade L, Marchi NM, Castro S, Díaz J, Faúndes A The effect upon the human vaginal histology of the long-term use of the injectable contraceptive Depo-Provera. Contraception. 2000;62(1):23–27. 11024225 10.1016/s0010-7824(00)00132-3

[159] Morrison C, Fichorova RN, Mauck C, Chen PL, Kwok C, Chipato T, Salata R, Doncel GF Cervical inflammation and immunity associated with hormonal contraception, pregnancy, and HIV-1 seroconversion. J Acquir Immune Defic Syndr. 2014;66(2):109–117. 24413042 10.1097/QAI.0000000000000103

[160] Fichorova RN, Chen PL, Morrison CS, Doncel GF, Mendonca K, Kwok C, Chipato T, Salata R, Mauck C The contribution of cervicovaginal infections to the immunomodulatory effects of hormonal contraception. MBio. 2015;6(5):e00221-15. 26330510 10.1128/mBio.00221-15 PMC4556810

[161] Li A, Felix JC, Yang W, Jain JK Effect of mifepristone on the expression of endometrial secretory leukocyte protease inhibitor in new medroxyprogesterone acetate users. Fertil Steril. 2008;90(3):872–875. 18155704 10.1016/j.fertnstert.2007.01.046

[162] Deese J, Masson L, Miller W, Cohen M, Morrison C, Wang M, Ahmed K, Agot K, Crucitti T, Abdellati S, Van Damme L Injectable progestin-only contraception is associated with increased levels of pro-inflammatory cytokines in the female genital tract. Am J Reprod Immunol. 2015;74(4):357–367. 26202107 10.1111/aji.12415

[163] Francis SC, Hou Y, Baisley K, van de Wijgert J, Watson-Jones D, Ao TT, Herrera C, Maganja K, Andreasen A, Kapiga S, Coulton GR, Hayes RJ, Shattock RJ Immune activation in the female genital tract: expression profiles of soluble proteins in women at high risk for HIV infection. PLoS One. 2016;11(1):e0143109. 26814891 10.1371/journal.pone.0143109 PMC4729472

[164] Roxby AC, Fredricks DN, Odem-Davis K, Ásbjörnsdóttir K, Masese L, Fiedler TL, De Rosa S, Jaoko W, Kiarie JN, Overbaugh J, McClelland RS Changes in vaginal microbiota and immune mediators in HIV-1-seronegative Kenyan women initiating depot medroxyprogesterone acetate. J Acquir Immune Defic Syndr. 2016;71(4):359–366. 26914908 10.1097/QAI.0000000000000866 PMC4770856

[165] Fleming DC, King AE, Williams AR, Critchley HO, Kelly RW Hormonal contraception can suppress natural antimicrobial gene transcription in human endometrium. Fertil Steril. 2003;79(4):856–863. 12749421 10.1016/s0015-0282(02)04930-0

[166] Goldfien GA, Barragan F, Chen J, Takeda M, Irwin JC, Perry J, Greenblatt RM, Smith-McCune KK, Giudice LC Progestin-containing contraceptives alter expression of host defense-related genes of the endometrium and cervix. Reprod Sci. 2015;22(7):814–828. 25634912 10.1177/1933719114565035 PMC4565478

[167] Ngcapu S, Masson L, Sibeko S, Werner L, McKinnon LR, Mlisana K, Shey M, Samsunder N, Karim SA, Karim QA, Passmore JA Lower concentrations of chemotactic cytokines and soluble innate factors in the lower female genital tract associated with the use of injectable hormonal contraceptive. J Reprod Immunol. 2015;110:14–21. 25956139 10.1016/j.jri.2015.03.007 PMC4779161

[168] Abel K, Rourke T, Lu D, Bost K, McChesney MB, Miller CJ Abrogation of attenuated lentivirus-induced protection in rhesus macaques by administration of Depo-Provera before intravaginal challenge with simian immunodeficiency virus mac239. J Infect Dis. 2004;190(9):1697–1705. 15478078 10.1086/424600 PMC3401018

[169] Genescà M, Li J, Fritts L, Chohan P, Bost K, Rourke T, Blozis SA, McChesney MB, Miller CJ Depo-Provera abrogates attenuated lentivirus-induced protection in male rhesus macaques challenged intravenously with pathogenic SIVmac239. J Med Primatol. 2007;36(4–5):266–275. 17669215 10.1111/j.1600-0684.2007.00244.x PMC3401015

[170] Hild-Petito S, Veazey RS, Larner JM, Reel JR, Blye RP Effects of two progestin-only contraceptives, Depo-Provera and Norplant-II, on the vaginal epithelium of rhesus monkeys. AIDS Res Hum Retroviruses. 1998;14(Suppl 1):S125–S130. 9581896

[171] Butler K, Ritter J, Ellis S, Henning TR, Montague J, Zaki S, Garber D, McNicholl JM, Kersh EN Analysis of putative mucosal SHIV susceptibility factors during repeated DMPA treatments in pigtail macaques. J Med Primatol. 2015;44(5):286–295. 26238265 10.1111/jmp.12188

[172] Kaushic C, Ashkar AA, Reid LA, Rosenthal KL Progesterone increases susceptibility and decreases immune responses to genital herpes infection. J Virol. 2003;77(8):4558–4565. 12663762 10.1128/JVI.77.8.4558-4565.2003 PMC152159

[173] Dietz Ostergaard S, Butler K, Ritter JM, Johnson R, Sanders J, Powell N, Lathrop G, Zaki SR, McNicholl JM, Kersh EN A combined oral contraceptive affects mucosal SHIV susceptibility factors in a pigtail macaque (*Macaca nemestrina*) model. J Med Primatol. 2015;44(2):97–107. 25536296 10.1111/jmp.12157 PMC4355188

[174] Goode D, Aravantinou M, Jarl S, Truong R, Derby N, Guerra-Perez N, Kenney J, Blanchard J, Gettie A, Robbiani M, Martinelli E Sex hormones selectively impact the endocervical mucosal microenvironment: implications for HIV transmission. PLoS One. 2014;9(5):e97767. 24830732 10.1371/journal.pone.0097767 PMC4022654

[175] Deligdisch L Hormonal pathology of the endometrium. Mod Pathol. 2000;13(3):285–294. 10757339 10.1038/modpathol.3880050

[176] Dinh A, Sriprasert I, Williams AR, Archer DF A review of the endometrial histologic effects of progestins and progesterone receptor modulators in reproductive age women. Contraception. 2015;91(5):360–367. 25596512 10.1016/j.contraception.2015.01.008

[177] Farage M, Maibach H Lifetime changes in the vulva and vagina. Arch Gynecol Obstet. 2006;273(4):195–202. 16208476 10.1007/s00404-005-0079-x

[178] Ferenczy A, Wright TC Anatomy and histology of the cervix In: Kurman R, ed. Blaustein’s Pathology of the Female Genital Tract. New York, NY: Springer-Verlag; 1994:185–201.

[179] Jacobson DL, Peralta L, Graham NM, Zenilman J Histologic development of cervical ectopy: relationship to reproductive hormones. Sex Transm Dis. 2000;27(5):252–258. 10821596 10.1097/00007435-200005000-00003

[180] Noyes RW Normal phases of the endometrium In: Norris JH, Hertig AT, Abell MR, eds. The Uterus. Baltimore, MD: Williams & Wilkins; 1973:110–135.

[181] Brenner RM, West NB Hormonal regulation of the reproductive tract in female mammals. Annu Rev Physiol. 1975;37(1):273–302. 164819 10.1146/annurev.ph.37.030175.001421

[182] Ferenczy A, Bertrand G, Gelfand MM Proliferation kinetics of human endometrium during the normal menstrual cycle. Am J Obstet Gynecol. 1979;133(8):859–867. 434029 10.1016/0002-9378(79)90302-8

[183] Moyer DL, Felix JC The effects of progesterone and progestins on endometrial proliferation. Contraception. 1998;57(6):399–403. 9693400 10.1016/s0010-7824(98)00047-x

[184] Bergeron C Morphological changes and protein secretion induced by progesterone in the endometrium during the luteal phase in preparation for nidation. Hum Reprod. 2000;15(Suppl 1):119–128. 10928424 10.1093/humrep/15.suppl_1.119

[185] Nonogaki H, Fujii S, Konishi I, Nanbu Y, Ozaki S, Ishikawa Y, Mori T Estrogen receptor localization in normal and neoplastic epithelium of the uterine cervix. Cancer. 1990;66(12):2620–2627. 2249202 10.1002/1097-0142(19901215)66:12<2620::aid-cncr2820661226>3.0.co;2-s

[186] Konishi I, Fujii S, Nonogaki H, Nanbu Y, Iwai T, Mori T Immunohistochemical analysis of estrogen receptors, progesterone receptors, Ki-67 antigen, and human papillomavirus DNA in normal and neoplastic epithelium of the uterine cervix. Cancer. 1991;68(6):1340–1350. 1651807 10.1002/1097-0142(19910915)68:6<1340::aid-cncr2820680626>3.0.co;2-q

[187] Sanborn BM, Held B, Kuo HS Hormonal action in human cervix—II. Specific progestogen binding proteins in human cervix. J Steroid Biochem. 1976;7(9):665–672. 979266 10.1016/0022-4731(76)90063-7

[188] Singer A The uterine cervix from adolescence to the menopause. Br J Obstet Gynaecol. 1975;82(2):81–99. 1125147 10.1111/j.1471-0528.1975.tb02204.x

[189] Jacobson DL, Peralta L, Farmer M, Graham NM, Wright TC, Zenilman J Cervical ectopy and the transformation zone measured by computerized planimetry in adolescents. Int J Gynaecol Obstet. 1999;66(1):7–17. 10458544 10.1016/s0020-7292(99)00037-5

[190] Mostad SB Prevalence and correlates of HIV type 1 shedding in the female genital tract. AIDS Res Hum Retroviruses. 1999;14(Suppl 1):S11–S15. 9581878

[191] Kyongo JK, Crucitti T, Menten J, Hardy L, Cools P, Michiels J, Delany-Moretlwe S, Mwaura M, Ndayisaba G, Joseph S, Fichorova R, van de Wijgert J, Vanham G, Ariën KK, Jespers V Cross-sectional analysis of selected genital tract immunological markers and molecular vaginal microbiota in sub-Saharan African women, with relevance to HIV risk and prevention. Clin Vaccine Immunol. 2015;22(5):526–538. 25761460 10.1128/CVI.00762-14 PMC4412937

[192] Myer L, Wright TC Jr, Denny L, Kuhn L Nested case-control study of cervical mucosal lesions, ectopy, and incident HIV infection among women in Cape Town, South Africa. Sex Transm Dis. 2006;33(11):683–687. 16614588 10.1097/01.olq.0000216026.67352.f9

[193] Tanton C, Weiss HA, Le Goff J, Changalucha J, Rusizoka M, Baisley K, Everett D, Ross DA, Belec L, Hayes RJ, Watson-Jones D Correlates of HIV-1 genital shedding in Tanzanian women. PLoS One. 2011;6(3):e17480. 21390251 10.1371/journal.pone.0017480 PMC3046975

[194] Buchanan DL, Kurita T, Taylor JA, Lubahn DB, Cunha GR, Cooke PS Role of stromal and epithelial estrogen receptors in vaginal epithelial proliferation, stratification, and cornification. Endocrinology. 1998;139(10):4345–4352. 9751518 10.1210/endo.139.10.6241

[195] Patton DL, Thwin SS, Meier A, Hooton TM, Stapleton AE, Eschenbach DA Epithelial cell layer thickness and immune cell populations in the normal human vagina at different stages of the menstrual cycle. Am J Obstet Gynecol. 2000;183(4):967–973. 11035348 10.1067/mob.2000.108857

[196] Gebhart JB, Rickard DJ, Barrett TJ, Lesnick TG, Webb MJ, Podratz KC, Spelsberg TC Expression of estrogen receptor isoforms alpha and beta messenger RNA in vaginal tissue of premenopausal and postmenopausal women. Am J Obstet Gynecol. 2001;185(6):1325–1330. 11744904 10.1067/mob.2001.119627

[197] Anderson DJ, Marathe J, Pudney J The structure of the human vaginal stratum corneum and its role in immune defense. Am J Reprod Immunol. 2014;71(6):618–623. 24661416 10.1111/aji.12230 PMC4024347

[198] Nilsson K, Risberg B, Heimer G The vaginal epithelium in the postmenopause—cytology, histology and pH as methods of assessment. Maturitas. 1995;21(1):51–56. 7731384 10.1016/0378-5122(94)00863-3

[199] Hickey DK, Patel MV, Fahey JV, Wira CR Innate and adaptive immunity at mucosal surfaces of the female reproductive tract: stratification and integration of immune protection against the transmission of sexually transmitted infections. J Reprod Immunol. 2011;88(2):185–194. 21353708 10.1016/j.jri.2011.01.005 PMC3094911

[200] Wira CR, Fahey JV A new strategy to understand how HIV infects women: identification of a window of vulnerability during the menstrual cycle. AIDS. 2008;22(15):1909–1917. 18784454 10.1097/QAD.0b013e3283060ea4 PMC2647143

[201] Wira CR, Fahey JV, Sentman CL, Pioli PA, Shen L Innate and adaptive immunity in female genital tract: cellular responses and interactions. Immunol Rev. 2005;206(1):306–335. 16048557 10.1111/j.0105-2896.2005.00287.x

[202] Kaushic C, Roth KL, Anipindi V, Xiu F Increased prevalence of sexually transmitted viral infections in women: the role of female sex hormones in regulating susceptibility and immune responses. J Reprod Immunol. 2011;88(2):204–209. 21296427 10.1016/j.jri.2010.12.004

[203] Quinn TC, Overbaugh J HIV/AIDS in women: an expanding epidemic. Science. 2005;308(5728):1582–1583. 15947174 10.1126/science.1112489

[204] Gray RH, Li X, Kigozi G, Serwadda D, Brahmbhatt H, Wabwire-Mangen F, Nalugoda F, Kiddugavu M, Sewankambo N, Quinn TC, Reynolds SJ, Wawer MJ Increased risk of incident HIV during pregnancy in Rakai, Uganda: a prospective study. Lancet. 2005;366(9492):1182–1188. 16198767 10.1016/S0140-6736(05)67481-8

[205] Mugo NR, Heffron R, Donnell D, Wald A, Were EO, Rees H, Celum C, Kiarie JN, Cohen CR, Kayintekore K, Baeten JM; Partners in Prevention HSV/HIV Transmission Study Team Increased risk of HIV-1 transmission in pregnancy: a prospective study among African HIV-1-serodiscordant couples. AIDS. 2011;25(15):1887–1895. 21785321 10.1097/QAD.0b013e32834a9338 PMC3173565

[206] Berger EA, Doms RW, Fenyö EM, Korber BT, Littman DR, Moore JP, Sattentau QJ, Schuitemaker H, Sodroski J, Weiss RA A new classification for HIV-1. Nature. 1998;391(6664):240. 9440686 10.1038/34571

[207] Coakley E, Petropoulos CJ, Whitcomb JM Assessing chemokine co-receptor usage in HIV. Curr Opin Infect Dis. 2005;18(1):9–15. 15647694 10.1097/00001432-200502000-00003

[208] Keele BF, Giorgi EE, Salazar-Gonzalez JF, Decker JM, Pham KT, Salazar MG, Sun C, Grayson T, Wang S, Li H, Wei X, Jiang C, Kirchherr JL, Gao F, Anderson JA, Ping LH, Swanstrom R, Tomaras GD, Blattner WA, Goepfert PA, Kilby JM, Saag MS, Delwart EL, Busch MP, Cohen MS, Montefiori DC, Haynes BF, Gaschen B, Athreya GS, Lee HY, Wood N, Seoighe C, Perelson AS, Bhattacharya T, Korber BT, Hahn BH, Shaw GM Identification and characterization of transmitted and early founder virus envelopes in primary HIV-1 infection. Proc Natl Acad Sci USA. 2008;105(21):7552–7557. 18490657 10.1073/pnas.0802203105 PMC2387184

[209] Selhorst P, Masson L, Ismail SD, Samsunder N, Garrett N, Mansoor LE, Abdool Karim Q, Abdool Karim SS, Passmore JS, Williamson C Cervicovaginal inflammation facilitates acquisition of less infectious HIV variants. Clin Infect Dis. 2017;64(1):79–82. 27694480 10.1093/cid/ciw663 PMC5159604

[210] Haynes BF, Shaw GM, Korber B, Kelsoe G, Sodroski J, Hahn BH, Borrow P, McMichael AJ HIV-host interactions: implications for vaccine design. Cell Host Microbe. 2016;19(3):292–303. 26922989 10.1016/j.chom.2016.02.002 PMC4823811

[211] Douek DC, Roederer M, Koup RA Emerging concepts in the immunopathogenesis of AIDS. Annu Rev Med. 2009;60(1):471–484. 18947296 10.1146/annurev.med.60.041807.123549 PMC2716400

[212] Meditz AL, Haas MK, Folkvord JM, Melander K, Young R, McCarter M, Mawhinney S, Campbell TB, Lie Y, Coakley E, Levy DN, Connick E HLA-DR^+^ CD38^+^ CD4^+^ T lymphocytes have elevated CCR5 expression and produce the majority of R5-tropic HIV-1 RNA in vivo. J Virol. 2011;85(19):10189–10200. 21813616 10.1128/JVI.02529-10 PMC3196402

[213] Micci L, Alvarez X, Iriele RI, Ortiz AM, Ryan ES, McGary CS, Deleage C, McAtee BB, He T, Apetrei C, Easley K, Pahwa S, Collman RG, Derdeyn CA, Davenport MP, Estes JD, Silvestri G, Lackner AA, Paiardini M CD4 depletion in SIV-infected macaques results in macrophage and microglia infection with rapid turnover of infected cells. PLoS Pathog. 2014;10(10):e1004467. 25356757 10.1371/journal.ppat.1004467 PMC4214815

[214] Hladik F, McElrath MJ Setting the stage: host invasion by HIV. Nat Rev Immunol. 2008;8(6):447–457. 18469831 10.1038/nri2302 PMC2587276

[215] Xu H, Wang X, Veazey RS Mucosal immunology of HIV infection. Immunol Rev. 2013;254(1):10–33. 23772612 10.1111/imr.12072 PMC3693769

[216] Hladik F, Sakchalathorn P, Ballweber L, Lentz G, Fialkow M, Eschenbach D, McElrath MJ Initial events in establishing vaginal entry and infection by human immunodeficiency virus type-1. Immunity. 2007;26(2):257–270. 17306567 10.1016/j.immuni.2007.01.007 PMC1885958

[217] Sagar M Origin of the transmitted virus in HIV infection: infected cells versus cell-free virus. J Infect Dis. 2014;210(Suppl 3):S667–S673. 25414422 10.1093/infdis/jiu369 PMC4303076

[218] Hel Z, McGhee JR, Mestecky J HIV infection: first battle decides the war. Trends Immunol. 2006;27(6):274–281. 16679064 10.1016/j.it.2006.04.007

[219] Anderson DJ, Le Grand R Cell-associated HIV mucosal transmission: the neglected pathway. J Infect Dis. 2014;210(Suppl 3):S606–S608. 25414413 10.1093/infdis/jiu538 PMC4303077

[220] Kell PD, Barton SE, Edmonds DK, Boag FC HIV infection in a patient with Meyer-Rokitansky-Küster-Hauser syndrome. J R Soc Med. 1992;85(11):706–707. 1474562 10.1177/014107689208501119 PMC1293736

[221] Miller CJ, Alexander NJ, Vogel P, Anderson J, Marx PA Mechanism of genital transmission of SIV: a hypothesis based on transmission studies and the location of SIV in the genital tract of chronically infected female rhesus macaques. J Med Primatol. 1992;21(2–3):64–68. 1433268

[222] Ferreira VH, Kafka JK, Kaushic C Influence of common mucosal co-factors on HIV infection in the female genital tract. Am J Reprod Immunol. 2014;71(6):543–554. 24617528 10.1111/aji.12221

[223] Stieh DJ, Maric D, Kelley ZL, Anderson MR, Hattaway HZ, Beilfuss BA, Rothwangl KB, Veazey RS, Hope TJ Vaginal challenge with an SIV-based dual reporter system reveals that infection can occur throughout the upper and lower female reproductive tract. PLoS Pathog. 2014;10(10):e1004440. 25299616 10.1371/journal.ppat.1004440 PMC4192600

[224] Mishell DR Jr, Kharma KM, Thorneycroft IH, Nakamura RM Estrogenic activity in women receiving an injectable progestogen for contraception. Am J Obstet Gynecol. 1972;113(3):372–376. 4637029 10.1016/0002-9378(72)90687-4

[225] Hickey M, Marino JL, Tachedjian G Critical review: mechanisms of HIV transmission in Depo-Provera users: the likely role of hypoestrogenism. J Acquir Immune Defic Syndr. 2016;71(1):1–7. 26761267 10.1097/QAI.0000000000000805

[226] Anipindi VC, Bagri P, Roth K, Dizzell SE, Nguyen PV, Shaler CR, Chu DK, Jiménez-Saiz R, Liang H, Swift S, Nazli A, Kafka JK, Bramson J, Xing Z, Jordana M, Wan Y, Snider DP, Stampfli MR, Kaushic C Estradiol enhances CD4^+^ T-cell anti-viral immunity by priming vaginal DCs to induce Th17 responses via an IL-1-dependent pathway. PLoS Pathog. 2016;12(5):e1005589. 27148737 10.1371/journal.ppat.1005589 PMC4858291

[227] Wira CR, Fahey JV, Rodriguez-Garcia M, Shen Z, Patel MV Regulation of mucosal immunity in the female reproductive tract: the role of sex hormones in immune protection against sexually transmitted pathogens. Am J Reprod Immunol. 2014;72(2):236–258. 24734774 10.1111/aji.12252 PMC4351777

[228] Marx PA, Spira AI, Gettie A, Dailey PJ, Veazey RS, Lackner AA, Mahoney CJ, Miller CJ, Claypool LE, Ho DD, Alexander NJ Progesterone implants enhance SIV vaginal transmission and early virus load. Nat Med. 1996;2(10):1084–1089. 8837605 10.1038/nm1096-1084

[229] Smith SM, Baskin GB, Marx PA Estrogen protects against vaginal transmission of simian immunodeficiency virus. J Infect Dis. 2000;182(3):708–715. 10950763 10.1086/315776

[230] Hummelen R, Macklaim JM, Bisanz JE, Hammond JA, McMillan A, Vongsa R, Koenig D, Gloor GB, Reid G Vaginal microbiome and epithelial gene array in post-menopausal women with moderate to severe dryness. PLoS One. 2011;6(11):e26602. 22073175 10.1371/journal.pone.0026602 PMC3206802

[231] Cotreau MM, Chennathukuzhi VM, Harris HA, Han L, Dorner AJ, Apseloff G, Varadarajan U, Hatstat E, Zakaria M, Strahs AL, Crabtree JS, Winneker RC, Jelinsky SA A study of 17β-estradiol-regulated genes in the vagina of postmenopausal women with vaginal atrophy. Maturitas. 2007;58(4):366–376. 17997058 10.1016/j.maturitas.2007.09.009

[232] Smith SM, Mefford M, Sodora D, Klase Z, Singh M, Alexander N, Hess D, Marx PA Topical estrogen protects against SIV vaginal transmission without evidence of systemic effect. AIDS. 2004;18(12):1637–1643. 15280774 10.1097/01.aids.0000131393.76221.cc

[233] Gillgrass AE, Fernandez SA, Rosenthal KL, Kaushic C Estradiol regulates susceptibility following primary exposure to genital herpes simplex virus type 2, while progesterone induces inflammation. J Virol. 2005;79(5):3107–3116. 15709030 10.1128/JVI.79.5.3107-3116.2005 PMC548484

[234] Bhavanam S, Snider DP, Kaushic C Intranasal and subcutaneous immunization under the effect of estradiol leads to better protection against genital HSV-2 challenge compared to progesterone. Vaccine. 2008;26(48):6165–6172. 18804503 10.1016/j.vaccine.2008.08.045

[235] Mestecky J, Moldoveanu Z, Smith PD, Hel Z, Alexander RC Mucosal immunology of the genital and gastrointestinal tracts and HIV-1 infection. J Reprod Immunol. 2009;83(1–2):196–200. 19853927 10.1016/j.jri.2009.07.005 PMC2802574

[236] Molander U, Milsom I, Ekelund P, Mellström D, Eriksson O Effect of oral oestriol on vaginal flora and cytology and urogenital symptoms in the post-menopause. Maturitas. 1990;12(2):113–120. 2255263 10.1016/0378-5122(90)90089-o

[237] Smith P Estrogens and the urogenital tract. Studies on steroid hormone receptors and a clinical study on a new estradiol-releasing vaginal ring. Acta Obstet Gynecol Scand Suppl. 1993;157:1–26. 8393609

[238] Castelo-Branco C, Cancelo MJ, Villero J, Nohales F, Juliá MD Management of post-menopausal vaginal atrophy and atrophic vaginitis. Maturitas. 2005;52(Suppl 1):S46–S52. 16139449 10.1016/j.maturitas.2005.06.014

[239] Wieser F, Hosmann J, Tschugguel W, Czerwenka K, Sedivy R, Huber JC Progesterone increases the number of Langerhans cells in human vaginal epithelium. Fertil Steril. 2001;75(6):1234–1235. 11384659 10.1016/s0015-0282(01)01796-4

[240] Klebanoff SJ, Coombs RW Viricidal effect of *Lactobacillus acidophilus* on human immunodeficiency virus type 1: possible role in heterosexual transmission. J Exp Med. 1991;174(1):289–292. 1647436 10.1084/jem.174.1.289 PMC2118880

[241] Martin HL, Richardson BA, Nyange PM, Lavreys L, Hillier SL, Chohan B, Mandaliya K, Ndinya-Achola JO, Bwayo J, Kreiss J Vaginal lactobacilli, microbial flora, and risk of human immunodeficiency virus type 1 and sexually transmitted disease acquisition. J Infect Dis. 1999;180(6):1863–1868. 10558942 10.1086/315127

[242] Taha TE, Hoover DR, Dallabetta GA, Kumwenda NI, Mtimavalye LA, Yang LP, Liomba GN, Broadhead RL, Chiphangwi JD, Miotti PG Bacterial vaginosis and disturbances of vaginal flora: association with increased acquisition of HIV. AIDS. 1998;12(13):1699–1706. 9764791 10.1097/00002030-199813000-00019

[243] Atashili J, Poole C, Ndumbe PM, Adimora AA, Smith JS Bacterial vaginosis and HIV acquisition: a meta-analysis of published studies. AIDS. 2008;22(12):1493–1501. 18614873 10.1097/QAD.0b013e3283021a37 PMC2788489

[244] Sanders-Beer B, Babas T, Mansfield K, Golightly D, Kramer J, Bowlsbey A, Sites D, Nieves-Duran L, Lin S, Rippeon S, Donnelly G, Rhodes L, Spano YE Depo-Provera does not alter disease progression in SIVmac-infected female Chinese rhesus macaques. AIDS Res Hum Retroviruses. 2010;26(4):433–443. 20377424 10.1089/aid.2009.0185 PMC2864058

[245] Sodora DL, Gettie A, Miller CJ, Marx PA Vaginal transmission of SIV: assessing infectivity and hormonal influences in macaques inoculated with cell-free and cell-associated viral stocks. AIDS Res Hum Retroviruses. 1998;14(Suppl 1):S119–S123. 9581895

[246] Vishwanathan SA, Guenthner PC, Lin CY, Dobard C, Sharma S, Adams DR, Otten RA, Heneine W, Hendry RM, McNicholl JM, Kersh EN High susceptibility to repeated, low-dose, vaginal SHIV exposure late in the luteal phase of the menstrual cycle of pigtail macaques. J Acquir Immune Defic Syndr. 2011;57(4):261–264. 21546848 10.1097/QAI.0b013e318220ebd3

[247] Poonia B, Walter L, Dufour J, Harrison R, Marx PA, Veazey RS Cyclic changes in the vaginal epithelium of normal rhesus macaques. J Endocrinol. 2006;190(3):829–835. 17003283 10.1677/joe.1.06873

[248] Mora G, Johansson ED Plasma levels of medroxyprogesterone acetate (MPA), estradiol and progesterone in the rhesus monkey after intramuscular adminstration of Depo-Provera. Contraception. 1976;14(3):343–350. 824097 10.1016/0010-7824(76)90101-3

[249] Kersh EN, Ritter J, Butler K, Ostergaard SD, Hanson D, Ellis S, Zaki S, McNicholl JM Relationship of estimated SHIV acquisition time points during the menstrual cycle and thinning of vaginal epithelial layers in pigtail macaques. Sex Transm Dis. 2015;42(12):694–701. 26562699 10.1097/OLQ.0000000000000367 PMC4646715

[250] Parr MB, Kepple L, McDermott MR, Drew MD, Bozzola JJ, Parr EL A mouse model for studies of mucosal immunity to vaginal infection by herpes simplex virus type 2. Lab Invest. 1994;70(3):369–380. 8145530

[251] World Health Organization Global incidence and prevalence of selected curable sexually transmitted infections—2008. Available at: http://apps.who.int/iris/handle/10665/75181. Accessed 31 December 2017.

[252] World Health Organization Sexually transmitted infections (STIs). Available at: http://www.who.int/mediacentre/factsheets/fs110/en/. Accessed 31 December 2017.

[253] Looker KJ, Garnett GP, Schmid GP An estimate of the global prevalence and incidence of herpes simplex virus type 2 infection. Bull World Health Organ. 2008;86(10):805–812. 18949218 10.2471/BLT.07.046128 PMC2649511

[254] Sexton J, Garnett G, Røttingen JA Metaanalysis and metaregression in interpreting study variability in the impact of sexually transmitted diseases on susceptibility to HIV infection. Sex Transm Dis. 2005;32(6):351–357. 15912081 10.1097/01.olq.0000154504.54686.d1

[255] Ward H, Rönn M Contribution of sexually transmitted infections to the sexual transmission of HIV. Curr Opin HIV AIDS. 2010;5(4):305–310. 20543605 10.1097/COH.0b013e32833a8844 PMC2923028

[256] Alkema L, Kantorova V, Menozzi C, Biddlecom A National, regional, and global rates and trends in contraceptive prevalence and unmet need for family planning between 1990 and 2015: a systematic and comprehensive analysis. Lancet. 2013;381(9878):1642–1652. 23489750 10.1016/S0140-6736(12)62204-1

[257] Darroch JE, Singh S Trends in contraceptive need and use in developing countries in 2003, 2008, and 2012: an analysis of national surveys. Lancet. 2013;381(9879):1756–1762. 23683642 10.1016/S0140-6736(13)60597-8

[258] Morrison CS, Turner AN, Jones LB Highly effective contraception and acquisition of HIV and other sexually transmitted infections. Best Pract Res Clin Obstet Gynaecol. 2009;23(2):263–284. 19211309 10.1016/j.bpobgyn.2008.11.004

[259] Mohllajee AP, Curtis KM, Martins SL, Peterson HB Hormonal contraceptive use and risk of sexually transmitted infections: a systematic review. Contraception. 2006;73(2):154–165. 16413846 10.1016/j.contraception.2005.08.012

[260] Venkatesh KK, Cu-Uvin S Assessing the relationship between cervical ectopy and HIV susceptibility: implications for HIV prevention in women. Am J Reprod Immunol. 2013;69(Suppl 1):68–73. 23057756 10.1111/aji.12029

[261] Roura E, Travier N, Waterboer T, de Sanjosé S, Bosch FX, Pawlita M, Pala V, Weiderpass E, Margall N, Dillner J, Gram IT, Tjønneland A, Munk C, Palli D, Khaw KT, Overvad K, Clavel-Chapelon F, Mesrine S, Fournier A, Fortner RT, Ose J, Steffen A, Trichopoulou A, Lagiou P, Orfanos P, Masala G, Tumino R, Sacerdote C, Polidoro S, Mattiello A, Lund E, Peeters PH, Bueno-de-Mesquita HB, Quirós JR, Sánchez MJ, Navarro C, Barricarte A, Larrañaga N, Ekström J, Lindquist D, Idahl A, Travis RC, Merritt MA, Gunter MJ, Rinaldi S, Tommasino M, Franceschi S, Riboli E, Castellsagué X The influence of hormonal factors on the risk of developing cervical cancer and pre-cancer: results from the EPIC cohort. PLoS One. 2016;11(1):e0147029. 26808155 10.1371/journal.pone.0147029 PMC4726518

[262] Grabowski MK, Gray RH, Makumbi F, Kagaayi J, Redd AD, Kigozi G, Reynolds SJ, Nalugoda F, Lutalo T, Wawer MJ, Serwadda D, Quinn TC, Tobian AAR Use of injectable hormonal contraception and women’s risk of herpes simplex virus type 2 acquisition: a prospective study of couples in Rakai, Uganda. Lancet Glob Health. 2015;3(8):e478–e486. 26094162 10.1016/S2214-109X(15)00086-8 PMC5537725

[263] Kaushic C HIV-1 infection in the female reproductive tract: role of interactions between HIV-1 and genital epithelial cells. Am J Reprod Immunol. 2011;65(3):253–260. 21223427 10.1111/j.1600-0897.2010.00965.x

[264] Morris MR, Byrareddy SN, Villinger F, Henning TC, Butler K, Ansari AA, McNicholl JM, Kersh EN Relationship of menstrual cycle and vaginal infection in female rhesus macaques challenged with repeated, low doses of SIVmac251. J Med Primatol. 2015;44(5):301–305. 26054016 10.1111/jmp.12177 PMC4626076

[265] McNicholl JM, Henning TC, Vishwanathan SA, Kersh EN Non-human primate models of hormonal contraception and HIV. Am J Reprod Immunol. 2014;71(6):513–522. 24716832 10.1111/aji.12246

[266] Jerse AE, Wu H, Packiam M, Vonck RA, Begum AA, Garvin LE Estradiol-treated female mice as surrogate hosts for *Neisseria gonorrhoeae* genital tract infections. Front Microbiol. 2011;2:107. 21747807 10.3389/fmicb.2011.00107 PMC3129519

[267] van de Wijgert JH, Verwijs MC, Turner AN, Morrison CS Hormonal contraception decreases bacterial vaginosis but oral contraception may increase candidiasis: implications for HIV transmission. AIDS. 2013;27(13):2141–2153. 23660575 10.1097/QAD.0b013e32836290b6

[268] Gilliet M, Cao W, Liu YJ Plasmacytoid dendritic cells: sensing nucleic acids in viral infection and autoimmune diseases. Nat Rev Immunol. 2008;8(8):594–606. 18641647 10.1038/nri2358

[269] Siegal FP, Kadowaki N, Shodell M, Fitzgerald-Bocarsly PA, Shah K, Ho S, Antonenko S, Liu YJ The nature of the principal type 1 interferon-producing cells in human blood. Science. 1999;284(5421):1835–1837. 10364556 10.1126/science.284.5421.1835

[270] Li Q, Estes JD, Schlievert PM, Duan L, Brosnahan AJ, Southern PJ, Reilly CS, Peterson ML, Schultz-Darken N, Brunner KG, Nephew KR, Pambuccian S, Lifson JD, Carlis JV, Haase AT Glycerol monolaurate prevents mucosal SIV transmission. Nature. 2009;458(7241):1034–1038. 19262509 10.1038/nature07831 PMC2785041

[271] Ballweber L, Robinson B, Kreger A, Fialkow M, Lentz G, McElrath MJ, Hladik F Vaginal Langerhans cells nonproductively transporting HIV-1 mediate infection of T cells. J Virol. 2011;85(24):13443–13447. 21976645 10.1128/JVI.05615-11 PMC3233146

[272] van den Berg LM, Ribeiro CM, Zijlstra-Willems EM, de Witte L, Fluitsma D, Tigchelaar W, Everts V, Geijtenbeek TB Caveolin-1 mediated uptake via langerin restricts HIV-1 infection in human Langerhans cells. Retrovirology. 2014;11(1):123. 25551286 10.1186/s12977-014-0123-7 PMC4301922

[273] Mayr L, Su B, Moog C Langerhans cells: the “yin and yang” of HIV restriction and transmission. Trends Microbiol. 2017;25(3):170–172. 28190635 10.1016/j.tim.2017.01.009

[274] Ildgruben AK, Sjöberg IM, Hammarström ML Influence of hormonal contraceptives on the immune cells and thickness of human vaginal epithelium. Obstet Gynecol. 2003;102(3):571–582. 12962945 10.1016/s0029-7844(03)00618-5

[275] Cherpes TL, Busch JL, Sheridan BS, Harvey SA, Hendricks RL Medroxyprogesterone acetate inhibits CD8^+^ T cell viral-specific effector function and induces herpes simplex virus type 1 reactivation. J Immunol. 2008;181(2):969–975. 18606648 10.4049/jimmunol.181.2.969 PMC2553693

[276] Schindler AE, Campagnoli C, Druckmann R, Huber J, Pasqualini JR, Schweppe KW, Thijssen JH Classification and pharmacology of progestins. Maturitas. 2003;46(Suppl 1):S7–S16. 14670641 10.1016/j.maturitas.2003.09.014

[277] Herold MJ, McPherson KG, Reichardt HM Glucocorticoids in T cell apoptosis and function. Cell Mol Life Sci. 2006;63(1):60–72. 16314919 10.1007/s00018-005-5390-y PMC2792342

[278] Zhou J, Cidlowski JA The human glucocorticoid receptor: one gene, multiple proteins and diverse responses. Steroids. 2005;70(5–7):407–417. 15862824 10.1016/j.steroids.2005.02.006

[279] Kino T, De Martino MU, Charmandari E, Mirani M, Chrousos GP Tissue glucocorticoid resistance/hypersensitivity syndromes. J Steroid Biochem Mol Biol. 2003;85(2-5):457–467. 12943736 10.1016/s0960-0760(03)00218-8

[280] Mirani M, Elenkov I, Volpi S, Hiroi N, Chrousos GP, Kino T HIV-1 protein Vpr suppresses IL-12 production from human monocytes by enhancing glucocorticoid action: potential implications of Vpr coactivator activity for the innate and cellular immunity deficits observed in HIV-1 infection. J Immunol. 2002;169(11):6361–6368. 12444143 10.4049/jimmunol.169.11.6361

[281] Ghosh D Glucocorticoid receptor-binding site in the human immunodeficiency virus long terminal repeat. J Virol. 1992;66(1):586–590. 1727502 10.1128/jvi.66.1.586-590.1992 PMC238321

[282] Kolesnitchenko V, Snart RS Regulatory elements in the human immunodeficiency virus type 1 long terminal repeat LTR (HIV-1) responsive to steroid hormone stimulation. AIDS Res Hum Retroviruses. 1992;8(12):1977–1980. 1493048 10.1089/aid.1992.8.1977

[283] Mitra D, Sikder SK, Laurence J Role of glucocorticoid receptor binding sites in the human immunodeficiency virus type 1 long terminal repeat in steroid-mediated suppression of HIV gene expression. Virology. 1995;214(2):512–521. 8553553 10.1006/viro.1995.0062

[284] Kino T, Kopp JB, Chrousos GP Glucocorticoids suppress human immunodeficiency virus type-1 long terminal repeat activity in a cell type-specific, glucocorticoid receptor-mediated fashion: direct protective effects at variance with clinical phenomenology. J Steroid Biochem Mol Biol. 2000;75(4–5):283–290. 11282284 10.1016/s0960-0760(00)00187-4

[285] Markham PD, Salahuddin SZ, Popovic M, Patel A, Veren K, Fladager A, Orndorff S, Gallo RC Advances in the isolation of HTLV-III from patients with AIDS and AIDS-related complex and from donors at risk. Cancer Res. 1985;45(9, Suppl):4588s–4591s. 2990690

[286] Markham PD, Salahuddin SZ, Veren K, Orndorff S, Gallo RC Hydrocortisone and some other hormones enhance the expression of HTLV-III. Int J Cancer. 1986;37(1):67–72. 3000956 10.1002/ijc.2910370112

[287] Soudeyns H, Geleziunas R, Shyamala G, Hiscott J, Wainberg MA Identification of a novel glucocorticoid response element within the genome of the human immunodeficiency virus type 1. Virology. 1993;194(2):758–768. 7684876 10.1006/viro.1993.1317

[288] Soudeyns H, Wainberg MA Effects of RU486 on HIV-1 replication. Nat Med. 1997;3(12):1302–1303. 10.1038/nm1297-1302b 9396582

[289] Wiegers K, Schwarck D, Reimer R, Bohn W Activation of the glucocorticoid receptor releases unstimulated PBMCs from an early block in HIV-1 replication. Virology. 2008;375(1):73–84. 18295813 10.1016/j.virol.2008.01.037

[290] Sheffield JS, Wendel GD Jr, McIntire DD, Norgard MV The effect of progesterone levels and pregnancy on HIV-1 coreceptor expression. Reprod Sci. 2009;16(1):20–31. 19144888 10.1177/1933719108325510

[291] Cabrera-Muñoz E, Fuentes-Romero LL, Zamora-Chávez J, Camacho-Arroyo I, Soto-Ramírez LE Effects of progesterone on the content of CCR5 and CXCR4 coreceptors in PBMCs of seropositive and exposed but uninfected Mexican women to HIV-1. J Steroid Biochem Mol Biol. 2012;132(1–2):66–72. 22342838 10.1016/j.jsbmb.2012.02.001

[292] Straub RH The complex role of estrogens in inflammation. Endocr Rev. 2007;28(5):521–574. 17640948 10.1210/er.2007-0001

[293] Mitchell CM, McLemore L, Westerberg K, Astronomo R, Smythe K, Gardella C, Mack M, Magaret A, Patton D, Agnew K, McElrath MJ, Hladik F, Eschenbach D Long-term effect of depot medroxyprogesterone acetate on vaginal microbiota, epithelial thickness and HIV target cells. J Infect Dis. 2014;210(4):651–655. 24652495 10.1093/infdis/jiu176 PMC4172039

[294] Byrareddy SN, Kallam B, Arthos J, Cicala C, Nawaz F, Hiatt J, Kersh EN, McNicholl JM, Hanson D, Reimann KA, Brameier M, Walter L, Rogers K, Mayne AE, Dunbar P, Villinger T, Little D, Parslow TG, Santangelo PJ, Villinger F, Fauci AS, Ansari AA Targeting α_4_β_7_ integrin reduces mucosal transmission of simian immunodeficiency virus and protects gut-associated lymphoid tissue from infection. Nat Med. 2014;20(12):1397–1400. 25419708 10.1038/nm.3715 PMC4257865

[295] Stieh DJ, Matias E, Xu H, Fought AJ, Blanchard JL, Marx PA, Veazey RS, Hope TJ Th17 cells are preferentially infected very early after vaginal transmission of SIV in macaques. Cell Host Microbe. 2016;19(4):529–540. 27078070 10.1016/j.chom.2016.03.005 PMC4841252

[296] Cecchinato V, Trindade CJ, Laurence A, Heraud JM, Brenchley JM, Ferrari MG, Zaffiri L, Tryniszewska E, Tsai WP, Vaccari M, Parks RW, Venzon D, Douek DC, O’Shea JJ, Franchini G Altered balance between Th17 and Th1 cells at mucosal sites predicts AIDS progression in simian immunodeficiency virus-infected macaques. Mucosal Immunol. 2008;1(4):279–288. 19079189 10.1038/mi.2008.14 PMC2997489

[297] Xu L, Dong B, Wang H, Zeng Z, Liu W, Chen N, Chen J, Yang J, Li D, Duan Y Progesterone suppresses Th17 cell responses, and enhances the development of regulatory T cells, through thymic stromal lymphopoietin-dependent mechanisms in experimental gonococcal genital tract infection. Microbes Infect. 2013;15(12):796–805. 23835188 10.1016/j.micinf.2013.06.012

[298] Semple F, Dorin JR β-Defensins: multifunctional modulators of infection, inflammation and more? J Innate Immun. 2012;4(4):337–348. 22441423 10.1159/000336619 PMC6784047

[299] McCormick TS, Weinberg A Epithelial cell-derived antimicrobial peptides are multifunctional agents that bridge innate and adaptive immunity. Periodontol 2000. 2010;54(1):195–206. 20712640 10.1111/j.1600-0757.2010.00373.x PMC3816379

[300] Quiñones-Mateu ME, Lederman MM, Feng Z, Chakraborty B, Weber J, Rangel HR, Marotta ML, Mirza M, Jiang B, Kiser P, Medvik K, Sieg SF, Weinberg A Human epithelial β-defensins 2 and 3 inhibit HIV-1 replication. AIDS. 2003;17(16):F39–F48. 14571200 10.1097/00002030-200311070-00001

[301] Weinberg A, Quiñones-Mateu ME, Lederman MM Role of human β-defensins in HIV infection. Adv Dent Res. 2006;19(1):42–48. 16672548 10.1177/154407370601900109

[302] Tugizov SM, Herrera R, Veluppillai P, Greenspan D, Soros V, Greene WC, Levy JA, Palefsky JM HIV is inactivated after transepithelial migration via adult oral epithelial cells but not fetal epithelial cells. Virology. 2011;409(2):211–222. 21056450 10.1016/j.virol.2010.10.004 PMC3034249

[303] Zapata W, Rodriguez B, Weber J, Estrada H, Quiñones-Mateu ME, Zimermman PA, Lederman MM, Rugeles MT Increased levels of human β-defensins mRNA in sexually HIV-1 exposed but uninfected individuals. Curr HIV Res. 2008;6(6):531–538. 18991618 10.2174/157016208786501463 PMC4126611

[304] Niyonsaba F, Ushio H, Nakano N, Ng W, Sayama K, Hashimoto K, Nagaoka I, Okumura K, Ogawa H Antimicrobial peptides human β-defensins stimulate epidermal keratinocyte migration, proliferation and production of proinflammatory cytokines and chemokines. J Invest Dermatol. 2007;127(3):594–604. 17068477 10.1038/sj.jid.5700599

[305] Varoga D, Pufe T, Harder J, Schröder JM, Mentlein R, Meyer-Hoffert U, Goldring MB, Tillmann B, Hassenpflug J, Paulsen F Human β-defensin 3 mediates tissue remodeling processes in articular cartilage by increasing levels of metalloproteinases and reducing levels of their endogenous inhibitors. Arthritis Rheum. 2005;52(6):1736–1745. 15934078 10.1002/art.21090

[306] Sørensen OE, Thapa DR, Rosenthal A, Liu L, Roberts AA, Ganz T Differential regulation of β-defensin expression in human skin by microbial stimuli. J Immunol. 2005;174(8):4870–4879. 15814714 10.4049/jimmunol.174.8.4870

[307] Kutteh WH, Mestecky J, Wira C Mucosal immunity in the human female reproductive tract In: Mestecky J, Lamm ME, Strober W, Bienenstock J, McGhee JR, Mayer L, eds. Mucosal Immunol. 3rd ed New York, NY: Elsevier Academic Press; 2005:1631–1646.

[308] Kutteh WH, Prince SJ, Hammond KR, Kutteh CC, Mestecky J Variations in immunoglobulins and IgA subclasses of human uterine cervical secretions around the time of ovulation. Clin Exp Immunol. 1996;104(3):538–542. 9099941 10.1046/j.1365-2249.1996.36742.x PMC2200440

[309] Bouman A, Heineman MJ, Faas MM Sex hormones and the immune response in humans. Hum Reprod Update. 2005;11(4):411–423. 15817524 10.1093/humupd/dmi008

[310] Kaushic C, Murdin AD, Underdown BJ, Wira CR *Chlamydia trachomatis* infection in the female reproductive tract of the rat: influence of progesterone on infectivity and immune response. Infect Immun. 1998;66(3):893–898. 9488372 10.1128/iai.66.3.893-898.1998 PMC107992

[311] Franklin RD, Kutteh WH Characterization of immunoglobulins and cytokines in human cervical mucus: influence of exogenous and endogenous hormones. J Reprod Immunol. 1999;42(2):93–106. 10221733 10.1016/s0165-0378(98)00086-2

[312] Klinger G, Gräser T, Mellinger U, Moore C, Vogelsang H, Groh A, Latterman C, Klinger G A comparative study of the effects of two oral contraceptives containing dienogest or desogestrel on the human immune system. Gynecol Endocrinol. 2000;14(1):15–24. 10813102 10.3109/09513590009167655

[313] Masson L, Passmore JA, Liebenberg LJ, Werner L, Baxter C, Arnold KB, Williamson C, Little F, Mansoor LE, Naranbhai V, Lauffenburger DA, Ronacher K, Walzl G, Garrett NJ, Williams BL, Couto-Rodriguez M, Hornig M, Lipkin WI, Grobler A, Abdool Karim Q, Abdool Karim SS Genital inflammation and the risk of HIV acquisition in women. Clin Infect Dis. 2015;61(2):260–269. 25900168 10.1093/cid/civ298 PMC4565995

[314] Passmore JA, Jaspan HB, Masson L Genital inflammation, immune activation and risk of sexual HIV acquisition. Curr Opin HIV AIDS. 2016;11(2):156–162. 26628324 10.1097/COH.0000000000000232 PMC6194860

[315] Africander D, Louw R, Verhoog N, Noeth D, Hapgood JP Differential regulation of endogenous pro-inflammatory cytokine genes by medroxyprogesterone acetate and norethisterone acetate in cell lines of the female genital tract. Contraception. 2011;84(4):423–435. 21920200 10.1016/j.contraception.2011.06.006

[316] Rollenhagen C, Asin SN Enhanced HIV-1 replication in ex vivo ectocervical tissues from post-menopausal women correlates with increased inflammatory responses. Mucosal Immunol. 2011;4(6):671–681. 21881573 10.1038/mi.2011.34

[317] Guthrie BL, Introini A, Roxby AC, Choi RY, Bosire R, Lohman-Payne B, Hirbod T, Farquhar C, Broliden K Depot medroxyprogesterone acetate use is associated with elevated innate immune effector molecules in cervicovaginal secretions of HIV-1-uninfected women. J Acquir Immune Defic Syndr. 2015;69(1):1–10. 25622059 10.1097/QAI.0000000000000533 PMC4424097

[318] Kurman RJ, Félix JC, Archer DF, Nanavati N, Arce J, Moyer DL Norethindrone acetate and estradiol-induced endometrial hyperplasia. Obstet Gynecol. 2000;96(3):373–379. 10960628 10.1016/s0029-7844(00)00944-3

[319] Eschenbach DA, Patton DL, Meier A, Thwin SS, Aura J, Stapleton A, Hooton TM Effects of oral contraceptive pill use on vaginal flora and vaginal epithelium. Contraception. 2000;62(3):107–112. 11124356 10.1016/s0010-7824(00)00155-4

[320] Kaushic C, Ferreira VH, Kafka JK, Nazli A HIV infection in the female genital tract: discrete influence of the local mucosal microenvironment. Am J Reprod Immunol. 2010;63(6):566–575. 20384619 10.1111/j.1600-0897.2010.00843.x

[321] Blaskewicz CD, Pudney J, Anderson DJ Structure and function of intercellular junctions in human cervical and vaginal mucosal epithelia. Biol Reprod. 2011;85(1):97–104. 21471299 10.1095/biolreprod.110.090423 PMC3123383

[322] Birse KD, Romas LM, Guthrie BL, Nilsson P, Bosire R, Kiarie J, Farquhar C, Broliden K, Burgener AD Genital injury signatures and microbiome alterations associated with depot medroxyprogesterone acetate usage and intravaginal drying practices. J Infect Dis. 2017;215(4):590–598. 28011908 10.1093/infdis/jiw590 PMC5388302

[323] Saba E, Origoni M, Taccagni G, Ferrari D, Doglioni C, Nava A, Lisco A, Grivel JC, Margolis L, Poli G Productive HIV-1 infection of human cervical tissue ex vivo is associated with the secretory phase of the menstrual cycle. Mucosal Immunol. 2013;6(6):1081–1090. 23385427 10.1038/mi.2013.2 PMC4153411

[324] Neidleman JA, Chen JC, Kohgadai N, Müller JA, Laustsen A, Thavachelvam K, Jang KS, Stürzel CM, Jones JJ, Ochsenbauer C, Chitre A, Somsouk M, Garcia MM, Smith JF, Greenblatt RM, Münch J, Jakobsen MR, Giudice LC, Greene WC, Roan NR Mucosal stromal fibroblasts markedly enhance HIV infection of CD4^+^ T cells. PLoS Pathog. 2017;13(2):e1006163. 28207890 10.1371/journal.ppat.1006163 PMC5312882

[325] Birse K, Arnold KB, Novak RM, McCorrister S, Shaw S, Westmacott GR, Ball TB, Lauffenburger DA, Burgener A Molecular signatures of immune activation and epithelial barrier remodeling are enhanced during the luteal phase of the menstrual cycle: implications for HIV susceptibility. J Virol. 2015;89(17):8793–8805. 26085144 10.1128/JVI.00756-15 PMC4524071

[326] Mirmonsef P, Hotton AL, Gilbert D, Burgad D, Landay A, Weber KM, Cohen M, Ravel J, Spear GT Free glycogen in vaginal fluids is associated with *Lactobacillus* colonization and low vaginal pH. PLoS One. 2014;9(7):e102467. 25033265 10.1371/journal.pone.0102467 PMC4102502

[327] Hummelen R, Fernandes AD, Macklaim JM, Dickson RJ, Changalucha J, Gloor GB, Reid G Deep sequencing of the vaginal microbiota of women with HIV. PLoS One. 2010;5(8):e12078. 20711427 10.1371/journal.pone.0012078 PMC2920804

[328] Anukam KC, Osazuwa E, Osemene GI, Ehigiagbe F, Bruce AW, Reid G Clinical study comparing probiotic *Lactobacillus* GR-1 and RC-14 with metronidazole vaginal gel to treat symptomatic bacterial vaginosis. Microbes Infect. 2006;8(12–13):2772–2776. 17045832 10.1016/j.micinf.2006.08.008

[329] Ravel J, Gajer P, Abdo Z, Schneider GM, Koenig SS, McCulle SL, Karlebach S, Gorle R, Russell J, Tacket CO, Brotman RM, Davis CC, Ault K, Peralta L, Forney LJ Vaginal microbiome of reproductive-age women. Proc Natl Acad Sci USA. 2011;108(Suppl 1):4680–4687. 20534435 10.1073/pnas.1002611107 PMC3063603

[330] Spear GT, Sikaroodi M, Zariffard MR, Landay AL, French AL, Gillevet PM Comparison of the diversity of the vaginal microbiota in HIV-infected and HIV-uninfected women with or without bacterial vaginosis. J Infect Dis. 2008;198(8):1131–1140. 18717638 10.1086/591942 PMC2800037

[331] Rampersaud R, Randis TM, Ratner AJ Microbiota of the upper and lower genital tract. Semin Fetal Neonatal Med. 2012;17(1):51–57. 21920833 10.1016/j.siny.2011.08.006 PMC3242913

[332] Watts DH, Fazzari M, Minkoff H, Hillier SL, Sha B, Glesby M, Levine AM, Burk R, Palefsky JM, Moxley M, Ahdieh-Grant L, Strickler HD Effects of bacterial vaginosis and other genital infections on the natural history of human papillomavirus infection in HIV-1-infected and high-risk HIV-1-uninfected women. J Infect Dis. 2005;191(7):1129–1139. 15747249 10.1086/427777

[333] Cherpes TL, Meyn LA, Krohn MA, Lurie JG, Hillier SL Association between acquisition of herpes simplex virus type 2 in women and bacterial vaginosis. Clin Infect Dis. 2003;37(3):319–325. 12884154 10.1086/375819

[334] Myer L, Denny L, Telerant R, Souza M, Wright TC Jr, Kuhn L Bacterial vaginosis and susceptibility to HIV infection in South African women: a nested case-control study. J Infect Dis. 2005;192(8):1372–1380. 16170754 10.1086/462427

[335] Sha BE, Zariffard MR, Wang QJ, Chen HY, Bremer J, Cohen MH, Spear GT Female genital-tract HIV load correlates inversely with *Lactobacillus* species but positively with bacterial vaginosis and *Mycoplasma hominis*. J Infect Dis. 2005;191(1):25–32. 15592999 10.1086/426394

[336] Cherpes TL, Melan MA, Kant JA, Cosentino LA, Meyn LA, Hillier SL Genital tract shedding of herpes simplex virus type 2 in women: effects of hormonal contraception, bacterial vaginosis, and vaginal group B *Streptococcus* colonization. Clin Infect Dis. 2005;40(10):1422–1428. 15844064 10.1086/429622

[337] Achilles SL, Hillier SL The complexity of contraceptives: understanding their impact on genital immune cells and vaginal microbiota. AIDS. 2013;27(Suppl 1):S5–S15. 24088684 10.1097/QAD.0000000000000058 PMC4012023

[338] Cohen CR, Lingappa JR, Baeten JM, Ngayo MO, Spiegel CA, Hong T, Donnell D, Celum C, Kapiga S, Delany S, Bukusi EA Bacterial vaginosis associated with increased risk of female-to-male HIV-1 transmission: a prospective cohort analysis among African couples. PLoS Med. 2012;9(6):e1001251. 22745608 10.1371/journal.pmed.1001251 PMC3383741

[339] Anahtar MN, Byrne EH, Doherty KE, Bowman BA, Yamamoto HS, Soumillon M, Padavattan N, Ismail N, Moodley A, Sabatini ME, Ghebremichael MS, Nusbaum C, Huttenhower C, Virgin HW, Ndung’u T, Dong KL, Walker BD, Fichorova RN, Kwon DS Cervicovaginal bacteria are a major modulator of host inflammatory responses in the female genital tract. Immunity. 2015;42(5):965–976. 25992865 10.1016/j.immuni.2015.04.019 PMC4461369

[340] Borgdorff H, Gautam R, Armstrong SD, Xia D, Ndayisaba GF, van Teijlingen NH, Geijtenbeek TB, Wastling JM, van de Wijgert JH Cervicovaginal microbiome dysbiosis is associated with proteome changes related to alterations of the cervicovaginal mucosal barrier. Mucosal Immunol. 2016;9(3):621–633. 26349657 10.1038/mi.2015.86

[341] Gareau MG, Sherman PM, Walker WA Probiotics and the gut microbiota in intestinal health and disease. Nat Rev Gastroenterol Hepatol. 2010;7(9):503–514. 20664519 10.1038/nrgastro.2010.117 PMC4748966

[342] Gill N, Finlay BB The gut microbiota: challenging immunology. Nat Rev Immunol. 2011;11(10):636–637. 21869815 10.1038/nri3061

[343] Karimi K, Inman MD, Bienenstock J, Forsythe P *Lactobacillus reuteri–*induced regulatory T cells protect against an allergic airway response in mice. Am J Respir Crit Care Med. 2009;179(3):186–193. 19029003 10.1164/rccm.200806-951OC

[344] Karimi K, Kandiah N, Chau J, Bienenstock J, Forsythe P A *Lactobacillus rhamnosus* strain induces a heme oxygenase dependent increase in Foxp3^+^ regulatory T cells. PLoS One. 2012;7(10):e47556. 23077634 10.1371/journal.pone.0047556 PMC3471882

[345] Mirmonsef P, Gilbert D, Zariffard MR, Hamaker BR, Kaur A, Landay AL, Spear GT The effects of commensal bacteria on innate immune responses in the female genital tract. Am J Reprod Immunol. 2011;65(3):190–195. 21143335 10.1111/j.1600-0897.2010.00943.x PMC3581076

[346] Mirmonsef P, Zariffard MR, Gilbert D, Makinde H, Landay AL, Spear GT Short-chain fatty acids induce pro-inflammatory cytokine production alone and in combination with Toll-like receptor ligands. Am J Reprod Immunol. 2012;67(5):391–400. 22059850 10.1111/j.1600-0897.2011.01089.x PMC3288536

[347] Doerflinger SY, Throop AL, Herbst-Kralovetz MM Bacteria in the vaginal microbiome alter the innate immune response and barrier properties of the human vaginal epithelia in a species-specific manner. J Infect Dis. 2014;209(12):1989–1999. 24403560 10.1093/infdis/jiu004

[348] Rebbapragada A, Howe K, Wachihi C, Pettengell C, Sunderji S, Huibner S, Ball TB, Plummer FA, Jaoko W, Kaul R Bacterial vaginosis in HIV-infected women induces reversible alterations in the cervical immune environment. J Acquir Immune Defic Syndr. 2008;49(5):520–522. 18989228 10.1097/QAI.0b013e318189a7ca

[349] Vodstrcil LA, Hocking JS, Law M, Walker S, Tabrizi SN, Fairley CK, Bradshaw CS Hormonal contraception is associated with a reduced risk of bacterial vaginosis: a systematic review and meta-analysis. PLoS One. 2013;8(9):e73055. 24023807 10.1371/journal.pone.0073055 PMC3762860

[350] Borgdorff H, Verwijs MC, Wit FW, Tsivtsivadze E, Ndayisaba GF, Verhelst R, Schuren FH, van de Wijgert JH The impact of hormonal contraception and pregnancy on sexually transmitted infections and on cervicovaginal microbiota in african sex workers. Sex Transm Dis. 2015;42(3):143–152. 25668647 10.1097/OLQ.0000000000000245

[351] Donders G, Bellen G, Janssens D, Van Bulck B, Hinoul P, Verguts J Influence of contraceptive choice on vaginal bacterial and fungal microflora. Eur J Clin Microbiol Infect Dis. 2017;36(1):43–48. 27638008 10.1007/s10096-016-2768-8

[352] Brooks JP, Buck GA, Chen G, Diao L, Edwards DJ, Fettweis JM, Huzurbazar S, Rakitin A, Satten GA, Smirnova E, Waks Z, Wright ML, Yanover C, Zhou YH Changes in vaginal community state types reflect major shifts in the microbiome. Microb Ecol Health Dis. 2017;28(1):1303265. 28572753 10.1080/16512235.2017.1303265 PMC5443090

[353] Brooks JP, Edwards DJ, Blithe DL, Fettweis JM, Serrano MG, Sheth NU, Strauss JF III, Buck GA, Jefferson KK Effects of combined oral contraceptives, depot medroxyprogesterone acetate and the levonorgestrel-releasing intrauterine system on the vaginal microbiome. Contraception. 2017;95(4):405–413. 27913230 10.1016/j.contraception.2016.11.006 PMC5376524

[354] De Seta F, Restaino S, De Santo D, Stabile G, Banco R, Busetti M, Barbati G, Guaschino S Effects of hormonal contraception on vaginal flora. Contraception. 2012;86(5):526–529. 22520642 10.1016/j.contraception.2012.02.012

[355] van de Wijgert JH, Verwijs MC, Turner AN, Morrison CS P3.221 Oral and injectable hormonal contraception decrease risk of bacterial vaginosis but oral contraception may increase risk of vaginal candidiasis: a systematic review of published and unpublished data. Sex Transm Infect. 2013;89(Suppl 1):A217.

[356] Segall-Gutierrez P, Du J, Niu C, Ge M, Tilley I, Mizraji K, Stanczyk FZ Effect of subcutaneous depot-medroxyprogesterone acetate (DMPA-SC) on serum androgen markers in normal-weight, obese, and extremely obese women. Contraception. 2012;86(6):739–745. 22959905 10.1016/j.contraception.2012.05.148

[357] Ishida Y, Mine T, Taguchi T Effect of progestins with different glucocorticoid activity on bone metabolism. Clin Endocrinol (Oxf). 2008;68(3):423–428. 17973947 10.1111/j.1365-2265.2007.03059.x

[358] Chi I What we have learned from recent IUD studies: a researcher’s perspective. Contraception. 1993;48(2):81–108. 8403915 10.1016/0010-7824(93)90001-n

[359] Sheppard BL Endometrial morphological changes in IUD users: a review. Contraception. 1987;36(1):1–10. 10.1016/0010-7824(87)90057-6 3117492

[360] Shobokshi A, Shaarawy M Cervical mucus granulocyte macrophage colony stimulating factor and interleukin-2 soluble receptor in women using copper intrauterine contraceptive devices. Contraception. 2002;66(2):129–132. 12204787 10.1016/s0010-7824(02)00331-1

[361] Ammälä M, Nyman T, Strengell L, Rutanen EM Effect of intrauterine contraceptive devices on cytokine messenger ribonucleic acid expression in the human endometrium. Fertil Steril. 1995;63(4):773–778. 7890061 10.1016/s0015-0282(16)57480-9

[362] Woolley JA, Seleem S, Hills FA, Salem H, el-Nashar E, Chard T Raised circulating levels of interleukin-6 in women with an intrauterine contraceptive device. Gynecol Obstet Invest. 1996;42(4):241–243. 8979095 10.1159/000291972

[363] Lahey T, Ghosh M, Fahey JV, Shen Z, Mukura LR, Song Y, Cu-Uvin S, Mayer KH, Wright PF, Kappes JC, Ochsenbauer C, Wira CR Selective impact of HIV disease progression on the innate immune system in the human female reproductive tract. PLoS One. 2012;7(6):e38100. 22675510 10.1371/journal.pone.0038100 PMC3366961

[364] Baeten JM, Lavreys L, Sagar M, Kreiss JK, Richardson BA, Chohan B, Panteleeff D, Mandaliya K, Ndinya-Achola JO, Overbaugh J, Farley T, Mwachari C, Cohen C, Chipato T, Jaisamrarn U, Kiriwat O, Duerr A Effect of contraceptive methods on natural history of HIV: studies from the Mombasa cohort. J Acquir Immune Defic Syndr. 2005;38(Suppl 1):S18–S21. 15867603 10.1097/01.qai.0000167030.18278.0e

[365] Stringer EM, Kaseba C, Levy J, Sinkala M, Goldenberg RL, Chi BH, Matongo I, Vermund SH, Mwanahamuntu M, Stringer JS A randomized trial of the intrauterine contraceptive device vs hormonal contraception in women who are infected with the human immunodeficiency virus. Am J Obstet Gynecol. 2007;197(2):144.e1–144.e8. 17689627 10.1016/j.ajog.2007.03.031 PMC2730754

[366] Mellors JW, Rinaldo CR Jr, Gupta P, White RM, Todd JA, Kingsley LA Prognosis in HIV-1 infection predicted by the quantity of virus in plasma. Science. 1996;272(5265):1167–1170. 8638160 10.1126/science.272.5265.1167

[367] Lavreys L, Baeten JM, Chohan V, McClelland RS, Hassan WM, Richardson BA, Mandaliya K, Ndinya-Achola JO, Overbaugh J Higher set point plasma viral load and more-severe acute HIV type 1 (HIV-1) illness predict mortality among high-risk HIV-1-infected African women. Clin Infect Dis. 2006;42(9):1333–1339. 16586394 10.1086/503258

[368] Stringer EM, Levy J, Sinkala M, Chi BH, Matongo I, Chintu N, Stringer JS HIV disease progression by hormonal contraceptive method: secondary analysis of a randomized trial. AIDS. 2009;23(11):1377–1382. 19448528 10.1097/QAD.0b013e32832cbca8 PMC4217202

[369] Cejtin HE, Jacobson L, Springer G, Watts DH, Levine A, Greenblatt R, Anastos K, Minkoff HL, Massad LS, Schmidt JB Effect of hormonal contraceptive use on plasma HIV-1-RNA levels among HIV-infected women. AIDS. 2003;17(11):1702–1704. 12853757 10.1097/00002030-200307250-00019

[370] Mostad SB, Overbaugh J, DeVange DM, Welch MJ, Chohan B, Mandaliya K, Nyange P, Martin HL Jr, Ndinya-Achola J, Bwayo JJ, Kreiss JK Hormonal contraception, vitamin A deficiency, and other risk factors for shedding of HIV-1 infected cells from the cervix and vagina. Lancet. 1997;350(9082):922–927. 9314871 10.1016/S0140-6736(97)04240-2

[371] Phillips SJ, Polis CB, Curtis KM The safety of hormonal contraceptives for women living with HIV and their sexual partners. Contraception. 2016;93(1):11–16. 26515194 10.1016/j.contraception.2015.10.002

[372] Polis CB, Phillips SJ, Curtis KM Hormonal contraceptive use and female-to-male HIV transmission: a systematic review of the epidemiologic evidence. AIDS. 2013;27(4):493–505. 23079808 10.1097/QAD.0b013e32835ad539

[373] Phillips SJ, Curtis KM, Polis CB Effect of hormonal contraceptive methods on HIV disease progression: a systematic review. AIDS. 2013;27(5):787–794. 23135169 10.1097/QAD.0b013e32835bb672

[374] Lawn SD, Butera ST, Folks TM Contribution of immune activation to the pathogenesis and transmission of human immunodeficiency virus type 1 infection. Clin Microbiol Rev. 2001;14(4):753–777. 11585784 10.1128/CMR.14.4.753-777.2001 PMC89002

[375] Wang CC, McClelland RS, Overbaugh J, Reilly M, Panteleeff DD, Mandaliya K, Chohan B, Lavreys L, Ndinya-Achola J, Kreiss JK The effect of hormonal contraception on genital tract shedding of HIV-1. AIDS. 2004;18(2):205–209. 15075537 10.1097/00002030-200401230-00009

[376] Clemetson DB, Moss GB, Willerford DM, Hensel M, Emonyi W, Holmes KK, Plummer F, Ndinya-Achola J, Roberts PL, Hillier S, et al Detection of HIV DNA in cervical and vaginal secretions. Prevalence and correlates among women in Nairobi, Kenya. JAMA. 1993;269(22):2860–2864. 8497089

[377] Heffron R, Donnell D, Rees H, Celum C, Mugo N, Were E, de Bruyn G, Nakku-Joloba E, Ngure K, Kiarie J, Coombs RW, Baeten JM; Partners in Prevention HSV/HIV Transmission Study Team Use of hormonal contraceptives and risk of HIV-1 transmission: a prospective cohort study. Lancet Infect Dis. 2012;12(1):19–26. 21975269 10.1016/S1473-3099(11)70247-X PMC3266951

[378] Gisselquist D, Rees H, Mugo N, Baeten JM Use of hormonal contraceptives and risk of HIV-1 transmission. Lancet Infect Dis. 2012;12(7):510–511, author reply 510–511. 22742633 10.1016/S1473-3099(12)70115-9

[379] Jain AK Hormonal contraception and HIV acquisition risk: implications for individual users and public policies. Contraception. 2012;86(6):645–652. 22541635 10.1016/j.contraception.2012.03.008

[380] Rodriguez MI, Reeves MF, Caughey AB Evaluating the competing risks of HIV acquisition and maternal mortality in Africa: a decision analysis. BJOG. 2012;119(9):1067–1073. 22676150 10.1111/j.1471-0528.2012.03402.x

